# An Android-Based Mobile App (ARVPredictor) for the Detection of HIV Drug-Resistance Mutations and Treatment at the Point of Care: Development Study

**DOI:** 10.2196/26891

**Published:** 2022-02-02

**Authors:** Beatrice Ongadi, Raphael Lihana, John Kiiru, Musa Ngayo, George Obiero

**Affiliations:** 1 Centre for Biotechnology and Bioinformatics University of Nairobi Nairobi Kenya; 2 Centre for Virus Research Kenya Medical Research Institute Nairobi Kenya; 3 Centre for Microbiology Research Kenya Medical Research Institute Nairobi Kenya

**Keywords:** database, mobile Android app, HIV/AIDS, mutation, pol gene, protease, reverse transcriptase, integrase, ARVPredictor, mobile app, mHealth, HIV, Android, digital health

## Abstract

**Background:**

HIV/AIDS remains one of the major global human health challenges, especially in resource-limited environments. By 2017, over 77.3 million people were infected with the disease, and approximately 35.4 million individuals had already died from AIDS-related illnesses. Approximately 21.7 million people were accessing ART with significant clinical outcomes. However, numerous challenges are experienced in the delivery and accurate interpretation of data on patients with HIV data by various health care providers at different care levels. Mobile health (mHealth) technology is progressively making inroads into the health sector as well as medical research. Different mobile devices have become common in health care settings, leading to rapid growth in the development of downloadable software specifically designed to fulfill particular health-related purposes.

**Objective:**

We developed a mobile-based app called ARVPredictor and demonstrated that it can accurately define HIV-1 drug-resistance mutations in the HIV pol gene for use at the point of care.

**Methods:**

ARVPredictor was designed using Android Studio with Java as the programming language and is compatible with both Android and iOS. The app system is hosted on Nginx Server, and network calls are built on PHP’s Laravel framework handled by the Retrofit Library. The DigitalOcean offers a high-performance and stable cloud computing platform for ARVPredictor. This mobile app is enlisted in the Google Play Store as an “ARVPredictor” and the source code is available under MIT permissive license at a GitHub repository. To test for agreement between the ARVPredictor and Stanford HIV Database in detecting HIV subtype and NNRT and NRTI mutations, a total of 100 known HIV sequences were evaluated.

**Results:**

The mobile-based app (ARVPredictor) takes in a set of sequences or known mutations (protease, reverse transcriptase and integrase). It then returns inferred levels of resistance to selected nucleoside, nonnucleoside protease, and integrase inhibitors for accurate HIV/AIDS management at the point of care. The ARVPredictor identified similar HIV subtypes in 98/100 sequences compared with the Stanford HIV Database (κ=0.98, indicating near perfect agreement). There were 89/100 major NNRTI and NRTI mutations identified by ARVPredictor, similar to the Stanford HIV Database (κ=0.89, indicating near perfect agreement). Eight mutations classified as major by the Stanford HIV Database were classified as others by ARVPredictor.

**Conclusions:**

The ARVPredictor largely agrees with the Stanford HIV Database in identifying both major and minor proteases, reverse transcriptase, and integrase mutations. The app can be conveniently used robustly at the point of care by HIV/AIDS care providers to improve the management of HIV infection.

## Introduction

The dynamic and exponential growth of economical mobile phones with sufficient built-in resources has been experienced worldwide today [1]. Business systems can currently leverage the strength of smartphones to affect long-distance transactions and undertake business consultations off-site robustly and accurately at low operating costs [2]. Mobile technology has progressively found vital utility in various health care platforms globally [3]. After the introduction of the first cellular phone by Motorola in the 1990s [4], faster processors, improved storage spaces, smaller longer life batteries, and superefficient mobile operating systems have been invented, paving avenues for wide useful application developments [5]. Mobile phones have become a common device in health care settings. This has in effect led to the proliferation of small self-contained pieces of downloadable software. Numerous medical software apps are now available to assist different health care professionals with many tasks, such as health record maintenance, time management, patient management and monitoring, clinical decision making, medical education and training, and online diagnosis [6]. In the recent past, mobile health (mHealth) has been used instrumentally to address various mental conditions [7], such as substance abuse disorders [8], depression [9], psychosis [10], and suicide. This and other related mHealth-based systematic reviews give an indication of the very positive future of human health care via smartphones [11,12].

Globally, by 2017, HIV/AIDS had infected over 77.3 million people, among which 35.4 million people or more have so far died from AIDS-related illness [13]. Kenya is among the worst hit countries, recording high prevalence and mortality rates alongside Mozambique and Uganda [14]. The advent of antiretroviral (ARV) therapy has led to a significant reduction in morbidity and mortality among different populations [15]. By 2017, over 21.7 million people were accessing antiretroviral therapy with significant clinical outcomes. Unfortunately, the development of HIV drug-resistance (HIVDR) mutations is currently jeopardizing these clinical benefits [16]. The United Nations Programme on HIV/AIDS (UNAIDS) 90-90-90 initiative [17] is also likely to be affected by increased HIV drug-resistant mutations, especially in developing countries. Therefore, drug-resistance testing is vital for accurate patient management [18]. Additionally, with the rolling out of “Treat all” in the HIV world, the occurrence of drug-resistant HIV is likely to become the key public health threat, thereby hindering treatment options available to people with HIV/AIDS [14]. The global prevalence of HIVDR is rising, mainly due to resistance to non-nucleoside reverse transcriptase inhibitor (NNRTI) drugs, which make up the backbone of World Health Organization’s (WHO) first-line antiretroviral treatment procedures [14]. Hence, there is an increase in the need for available and easy-to-access viral load testing avenues and other patient monitoring methods to alleviate the rise in HIVDR at the point of care [19].

ARVPredictor, which was designed and developed in this study, is a mobile app that bridges the glaring gap by providing point-of-care results to clinicians, most of whom in Kenya are now trained on HIVDR result interpretation. It comes with the advantages of mobile telephony and the capability to serialize evolutionary and drug-related sequence variation in HIV reverse transcriptase and protease, which are targeted by different antiretroviral drugs. It is enlisted in the Google Play Store as “ARVPredictor” [20]. The app is designed using Android Studio with Java as the programming language and is compatible for both Android and iOS with user-friendly graphical interfaces. This app accurately predicts HIVDR mutations in the *pol* region similar to those obtained from the web-based Stanford HIV Drug Resistance Database (HIVdb) [21,22].

## Methods

### Design and Setting

We obtained a replicated worksheet for developing ARVPredictor from different drug-resistance databases. The list included mutations existing in the ANRS (the French National Agency for Research on AIDS), HIVdb, IAS–USA (International Antiviral Society–USA), Los Alamos, and Rega algorithm lists [23]. We summarized the final register to a total of 19 normalized core tables, 10 lookup tables, and up to 20 derived tables. This was in line with other simulated databases mostly implemented using MySQL on Linux platforms. We ended up with ordered relationships linking main entities within, including antiretroviral therapy history, isolated drug-susceptibility outcome, and patient plasma HIV-1 RNA levels. The final database allows users to retrieve and analyze different sets of sequences that meet particular criteria. Common queries envisioned included retrieval of sequences of HIV-1 isolates containing mutations at specific positions, patients receiving a specific drug regimen, and drug-susceptibility data on HIV-1 isolates containing combinations of mutations. Every designed query provided the following category of data: hyperlinks to MEDLINE and GenBank records, a list of mutations in the sequence, a classification of the sequence, drug-susceptibility results, and some technical data. We availed options for downloading or viewing raw sequence data at the back end; each predesigned table returned 8 or more columns of data.

### Participants

To test the app’s usability, a random population comprising 100 health practitioners actively involved in HIV/AIDS management, app developers, and information and communications technology (ICT) students was recruited. The health practitioners enrolled in the usability survey included 10 HIV experts from the Kenya HIVDR Technical Working Group on drug-resistance approval and regimen guidance, 25 HIV scientists based at the Kenya Medical Research Institute and various universities in Kenya. Others included 20 medical and pharmacy students from the University of Nairobi, Kenya, 20 graduates of the National Advanced HIV Clinical Course (NAHCC) class of 2015, 10 app developers, and 15 ICT students from Jomo Kenyatta University of Agriculture and Technology, Kenya. All the enrolled health practitioners were required to own or have access to Android-based smartphones; those who needed intensive hands-on training on downloading and using their smartphones were automatically excluded in the survey. The health practitioners were invited to participate in this survey using open invites through email to selected human health research institutions, universities (especially ICT and virology departments), and HIV comprehensive care centers. The survey also invited other participants directly via phone calls. Participants willing to be engaged recorded their interest through replying to our email by filling out a short acceptance/consenting online form and were enrolled on a first-come-first-enrolled basis. Those selected received a link on how to download and use our ARVPredictor Android app for test purposes.

### App Development Process

**Figure 1:**
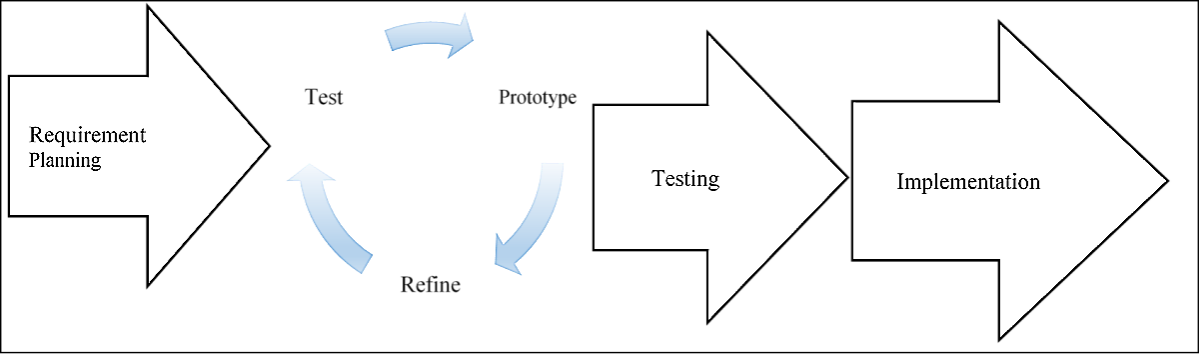
The ARVPredictor rapid application development model.

We designed and developed ARVPredictor from models of a combination of software development life cycle methodology [24] and rapid application development (RAD) [25]. RAD is an agile strategy for developing software that has proven to be fast and helps complete a project within a shorter timeframe. It achieves this by reducing the time spent in planning and maximizing prototyping development. The RAD enables faster communication between a developer and the end user; hence, its high efficiency is achieved by following 4 main phases at a reasonably lower cost [26]. The initial working prototype is developed and is improved gradually through discussions until a satisfying output is reached (Figure 1).

### Gap Identification and Requirements Gathering

A combination of 3 main activities was carried out at the initial stages of the development to meet both functional and nonfunctional requirements of the app. A brief questionnaire was randomly administered to consented selected HIV/AIDS doctors/clinicians and health care providers to determine and understand the current treatment process. This was followed by a face-to-face chat with a few health care providers who were very keen in using their smartphones to support their daily services to their patients. By performing a quick analysis of the results and with reference to relevant literature, a glaring gap was identified in the turnaround time and availability of point-of-care resources for interpreting the genetically modified HIV strains for appropriate antiretroviral drug choices. It was also understood that with the current mobile telephony systems, different forms of data can now be shared easily among various devices [27]. To create the initial prototype of the app, the output identified during this stage was translated into modules for better understanding and ownership of the whole app [28].

### Use Case Modeling

**Figure 2:**
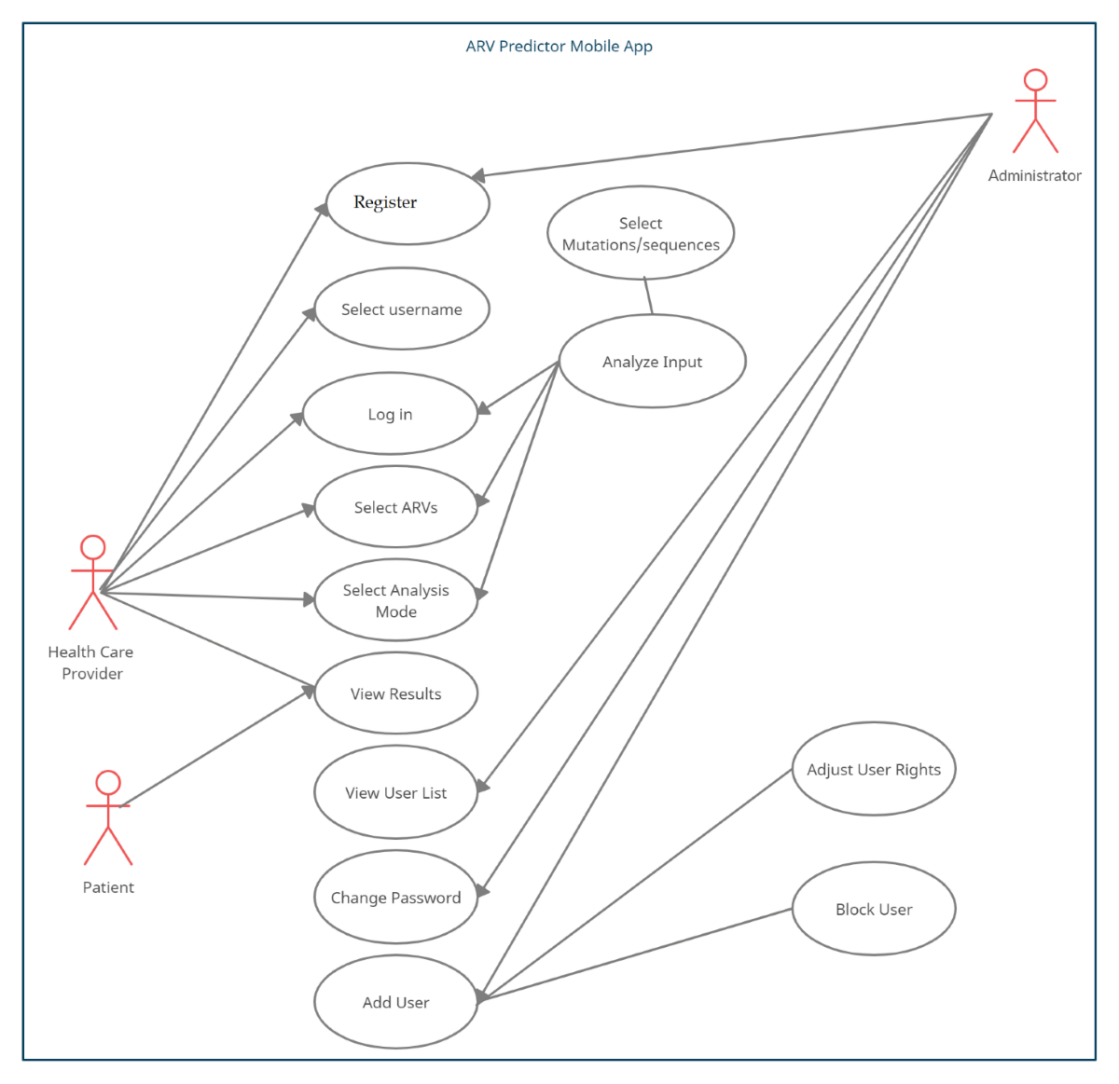
ARVPredictor use case diagram illustrating graphical interaction between users and the app. ARV: antiretroviral.

Use case diagrams show the interaction between users and the system [29]. In this app, we first identified the main actors and their respective interactions with the app. Each identified role was assigned relevant access rights and hence classified as *patient, health care givers,* and *administrators* of the system (Figure 2). Administrators have the capability to manage the critical functions of the app, whereas a normal user has very limited access to the back end functionality. By signing in using an existing email address and an active phone number, a normal user can query the database using mutations/sequences. A willing patient also has the capability to view the analysis output with predefined limitations.

### Operational Process of the App

A simple plain text prototype identified several app components, including mobile clients, web application clients, real-time servers, and databases. Following that, the development environment was set up for full development of the mobile app based on the various hardware and software specifications listed in the following subsections.

### Android Studio 4.1 and Java 10

The team developed this Android app using Android Studio 4.1 [30] and Java version 10 [31] as the programming language. Android Studio is the official Integrated Development Environment (IDE) for Android development and usually includes several aspects required for building various Android apps. It is based on IntelliJ IDEA [32] with powerful code editors and other developer-preferred tools. Integrated aspects of IntelliJ IDEA are essential in maximizing productivity with intelligent coding support. This aspect of the tool proved very handy in code management and provided coding hints during the development of ARVPredictor.

The Java programming language used in this app was developed in early 1910 by Sun Microsystems [33]. It is simple and efficient and can be used for various programming purposes. The combined and ultimate strength of these tools provided a suitable development platform for this app.

### Nginx Server Version 1.17.0

The development of this app involved Nginx Server [34] written in C programming language. This is an open source and a high-performance development platform. It is characterized by reasonable resource consumption, ease of configuration, stability, and a comprehensive feature set. Nginx supports both a high-performance HTTP server and a reverse proxy together with POP3/IMAP proxy servers. The developed app (ARVPredictor) is hosted on the Nginx server with DigitalOcean Droplets [35] offering the cloud hosting platform.

### MySQL

**Figure 3:**
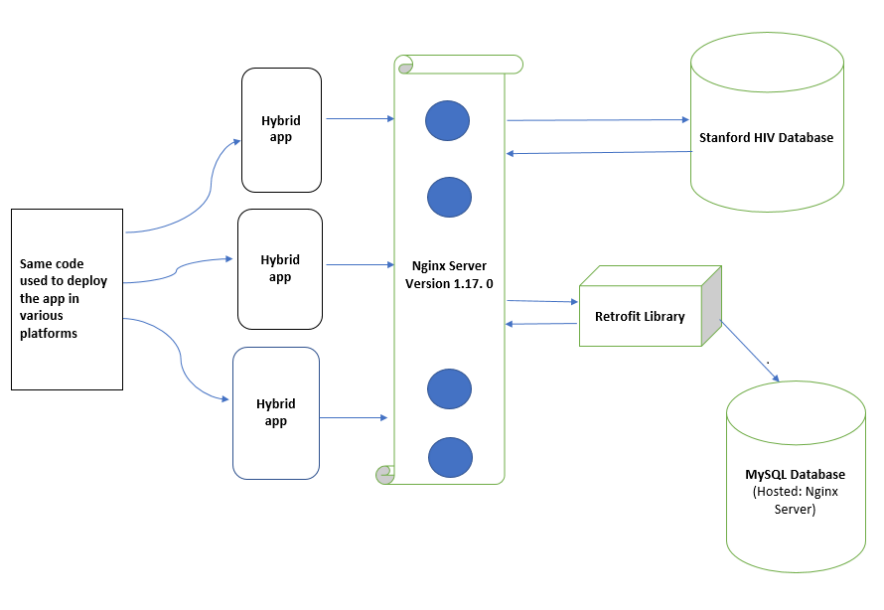
App development stack: back end arrangement of numerous connecting devices, main server, and active connection between the app (ARVPredictor) and Stanford HIVdb [21,22].

We projected massive growth of data in ARVPredictor and used MySQL [36], which is an open source document database written in C++. It is capable of creating and deploying a highly scalable database with high performance capability. It was selected due to its ability to work across platforms, high querying capability, availability, and predictable online professional support. The MySQL database management system has the capability to store data from a single record to a large amount of information. User data in ARVPredictor are stored in the MySQL database hosted on the same Nginx Server version 1.17.0 [34]. The actual arrangement of the development heap comprising mobile phones, servers, and databases is presented in Figure 3.

### Apollo Android

The ARVPredictor app was developed to connect to the Stanford Database [21] query language (GraphQL) through the Apollo Android library [37]. This platform converts and transfers data between the HIV Stanford Database and the ARVPredictor user interface.

### Retrofit Android Library

This is a rest client library [38]; in this app, retrofitting was used to handle all network calls from the app’s back end built on PHP’s Laravel framework.

### Mobile Client Environment

To ensure proper functionality and accurate returning of results, the app requires phone-free space of not less than 50 MB, touch screen display of 3.0 inches or higher, Android OS version 3.2 or later, and adequate data bundles.

### App System Design

**Figure 4:**
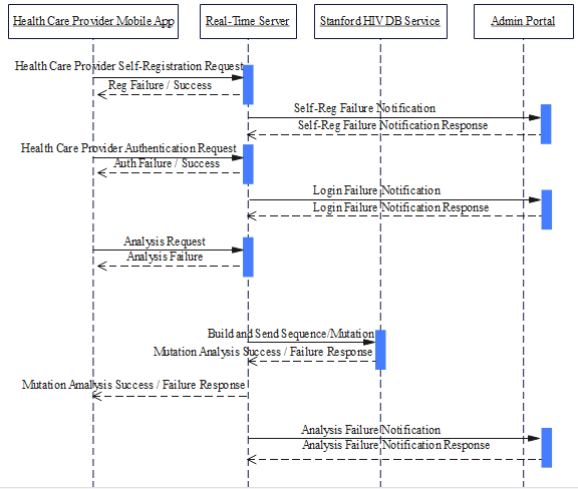
Application system design and preliminary testing procedure: Model indicating all probable activities by the user of ARVPredictor and predictable respective responses by both real-time server and Stanford HIVdb.

The design of the system was created and subjected to a simple objective versus output evaluation to ensure that all aspects of the app requirement were captured (Figure 4). The health care provider signs up using a valid and authenticated email address and uses a phone number and a password to access the app. A real-time server is set to build and send sequence or mutation files to the Stanford Database and return the expected analysis outcome. Different variables are captured immediately into an Excel sheet (Microsoft) and available for statistical analysis of the app.

### ARVPredictor Deployment and Testing

ARVPredictor is hosted on the Nginx server with DigitalOcean Linux Droplets offering the cloud hosting platform. For security, public access rights were set to be limited for the MySQL database. The Android app is freely distributed through Google Play (Google Play Store). To use ARVPredictor, users first need to create an account with very limited personal details (eg, valid email addresses, passwords, and preferred active phone numbers; see Multimedia Appendix 1). We deemed data gathered during the sign-in process as important for future growth of the project and would be kept under controlled access. The fully developed mobile app was then subjected to 2 levels of evaluation and testing, first through random entry of data to ensure system component interactivity and proper functionality. The second is to guarantee accurate analysis and result output. Black-box testing was used where the functionality of each simple app was subjected to testing without minding its internal structures or workings. This preliminary testing helped to reduce the cost and time spent in the final testing stages of the app development.

### ARVPredictor Maintenance

Regular and administrative monitoring steps were set up to assist in the continuous review of the app’s system logs. We considered this information useful in understanding and maintaining the app’s service health status. The output of this process informs maintenance-related needs such as any faults, downtimes, and any unauthorized activities. It helps in debugging and resolving any issues that arise as well as provides a platform for future upgrades.

### Input of Actual Data for Analysis

Two types of data, namely, sequences and point mutations, are usable for analysis in ARVPredictor. The said sequences can be keyed in or pasted directly onto the screen of the phone or alternatively uploaded from a separate file. HIV point mutations are preconfigured and are selectable based on the WHO 2009 listing [23]; both can be analyzed and confirmed according to the latest available version of the Stanford Database [21].

### Test Performance

The test performance of ARVPredictor against the Stanford HIV Database to determine HIV subtypes and both major and minor proteases, reverse transcriptase, and integrase mutations was determined using kappa statistics [39]. Accuracy of the ARVPredictor was then determined. A set of 100 sequences was blasted using both platforms, and the similarity was identified (Multimedia Appendix 2). The subtypes and minor and major mutations were noted for both platforms. To test for method accuracy, each sequence was blasted 3 times, and in each case, any variation (if any) in the subtype, base pairs, and mutations was recorded.

## Results

### Overview

The mobile app ARVPredictor was designed to take in data on protease, reverse transcriptase, and integrase mutations or sequences. It returns inferred levels of resistance to selected proteases, nucleosides, non-nucleosides, and integrase inhibitors for accurate HIV/AIDS management at the point of care.

### Sequence Analysis

We demonstrate the value and usability of ARVPredictor by analyzing different sets of reference sequences from the National Center for Biotechnology Information (NCBI) and other related studies. From numerous test results, we diagrammatically present sampled comparison results for the ARVPredictor against the gold-standard Stanford HIV Database [21]. The test sequence data set is presented in Textbox 1.

**Test sequence data.**

Accession No: KX505361.1: HIV-1 isolate 5873 from Kenya pol protein (pol)gene, partial cds [40].

AATGGCCATTGACRGAAGAAAAAATAAAGGCATTGATAGAAATTTGTACAGAGATGGAAAAGGAAGGAAA

AATTTCAAGAATTGGGCCTGAGAATCCATACAATACTCCAGTATTTGCCATAAAAARGAAAGACAGTACT

AAGTGGAGAAAATTAGTAGATTTCAGGGAACTCAATAAAAGAACCCAAGACTTTTGGGAAGTTCAATTAG

GRATACCACACCCAGCAGGGTTAAAARAGAAAAAATCAGYGACAGTACTAGATGTGGGGGATGCRTATTT

TTCAGTWCCTTTAGATGAAAGCTTCAGGAAATATACTGCATTYACCATACCRAGTRTAAACAATGAGACA

CCAGGAATCAGRTATCAGTACAATGTGCTTCCACAAGGATGGAAAGGATCACCRGCAATATTCCAAGCTA

GCATGACAAAAATYCTGGAACCTTTTAGGAAACAAAATCCAGAAATGATTATCTATCAATACATGGATGA

TTTGTATGTAGGATCTGACTTAGAAATAGGGCAACATAGAGCAAAAATAGAGRAATTAAGGGAACACCTG

TTAAAGTGGGGGTTTACTACACCAGACAAAAAGCATCAGAAAGAACCTCCAYTCCTTTGGATTGGTTAT

### Sequence Comparative Output

**Figure 5:**
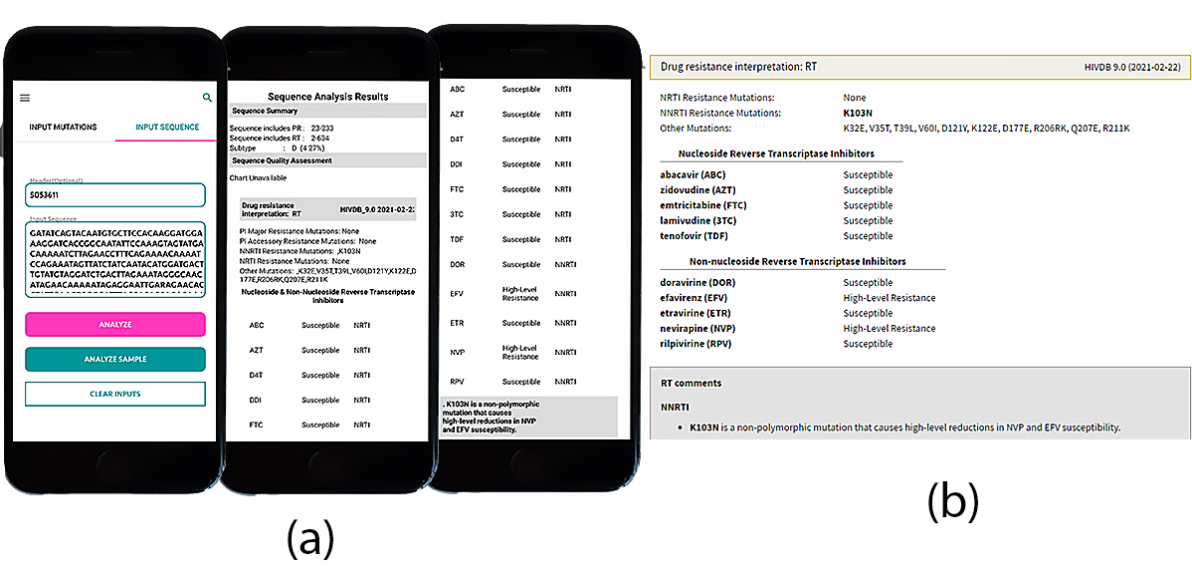
Sequence analysis results: Demonstrating similarity between sequence analysis results output for both (a) ARVPredictor and (b) Stanford HIV Database.

Figure 5 shows comparable sequence analysis results for both the Stanford Database [21] and ARVPredictor.

### Mutation Analysis

**Figure 6:**
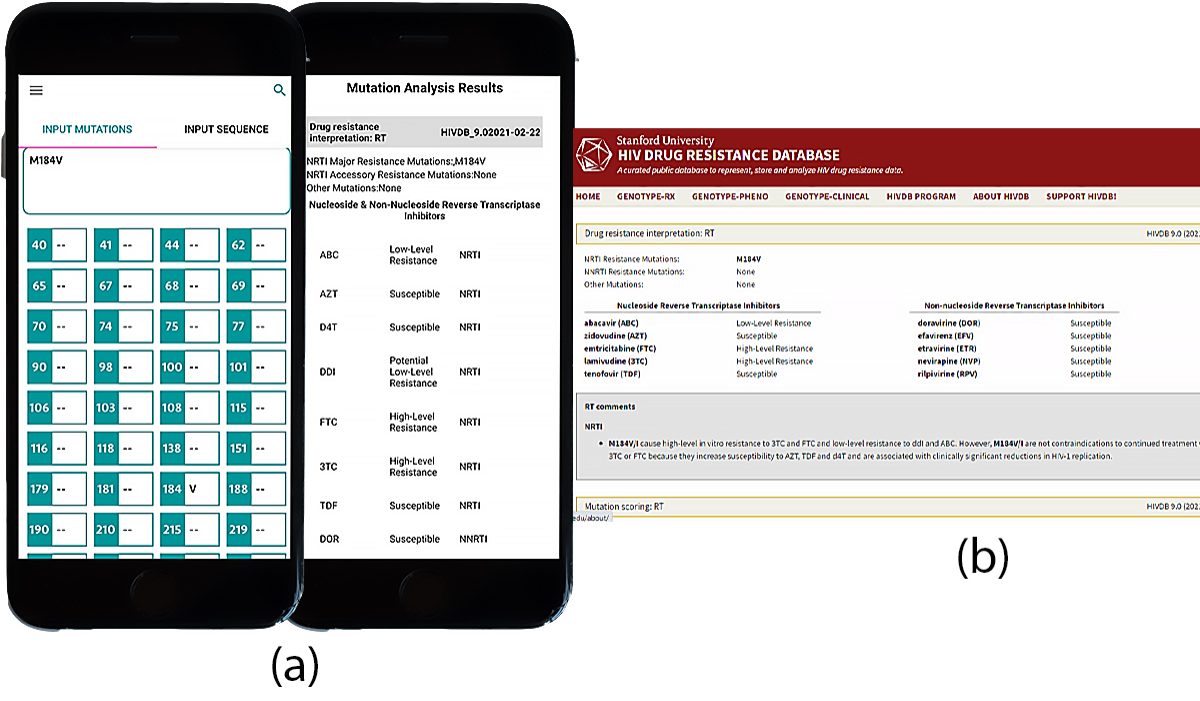
Mutation Analysis Results. Demonstrating similarity between mutation analysis results for (a) ARVPredictor and (b) Stanford HIV Database.

From the WHO Major HIV-1 Drug Resistance Mutations list [23], we tabulated the analysis results of the predefined HIV mutation M184V. The most common resistance mutations occur in nucleoside reverse transcriptase inhibitors (NRTIs) in vitro for both the ARVPredictor and Stanford Database. There is a high level of resistance to emtricitabine (FTC) and lamivudine (3TC) and potentially low level of resistance to didanosine (DDI) while displaying susceptibility to both zidovudine (ZDV) and tenofovir (TDV). The results shown in Figure 6 are comparable.

### Test Performance and Agreement

Multimedia Appendix 3 summarizes sequences with variation in subtype and mutations as determined by both the ARVPredictor app and the Stanford Database. The ARVPredictor app identified similar HIV subtypes in 98/100 sequences compared with the Stanford HIV Database (κ=0.98, indicating near perfect agreement). There were 89/100 major NNRTI and NRTI mutations identified by ARVPredictor, similar to the Stanford HIV Database (κ=0.89, indicating near perfect agreement). Seven mutations classified as major mutations by the Stanford HIV Database were classified as other mutations by ARVPredictor. This further indicates that the Stanford-confirmed GraphQL web service works fairly well, and all the results are in sync with most parts of the web version. Both tools found several minor/other mutations, but depending on small phone display window, some may be hidden from view.

### ARVPredictor Availability

**Figure 7:**
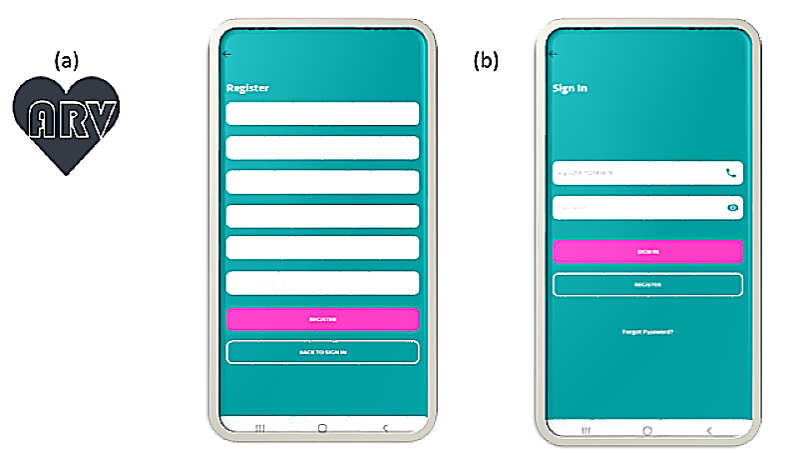
ARVPredictor Preliminary User Interfaces. (a) The pictorial icon shows how ARVPredictor appears ready for download from the app/play store. (b) Potential users must register in order to use the application, registered users are required to sign-in.

**Figure 8:**
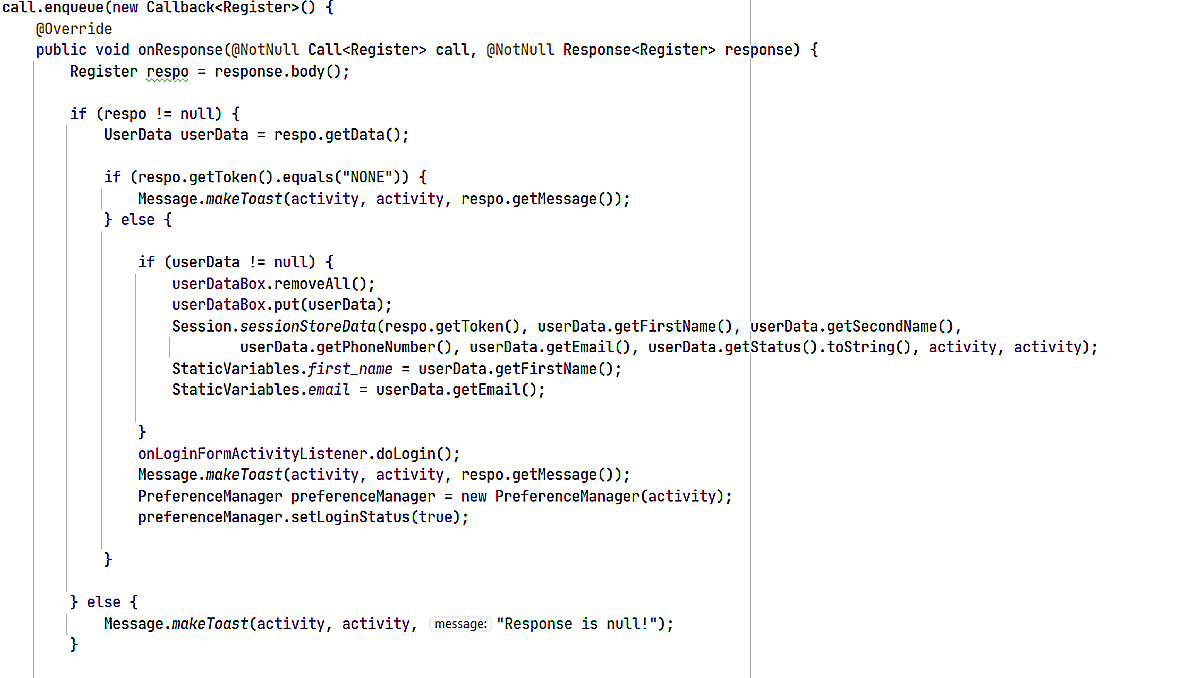
User Registration and Sign in source code: Set of computer logical instructions aiding registration and sign in process before using the app.

**Figure 9:**
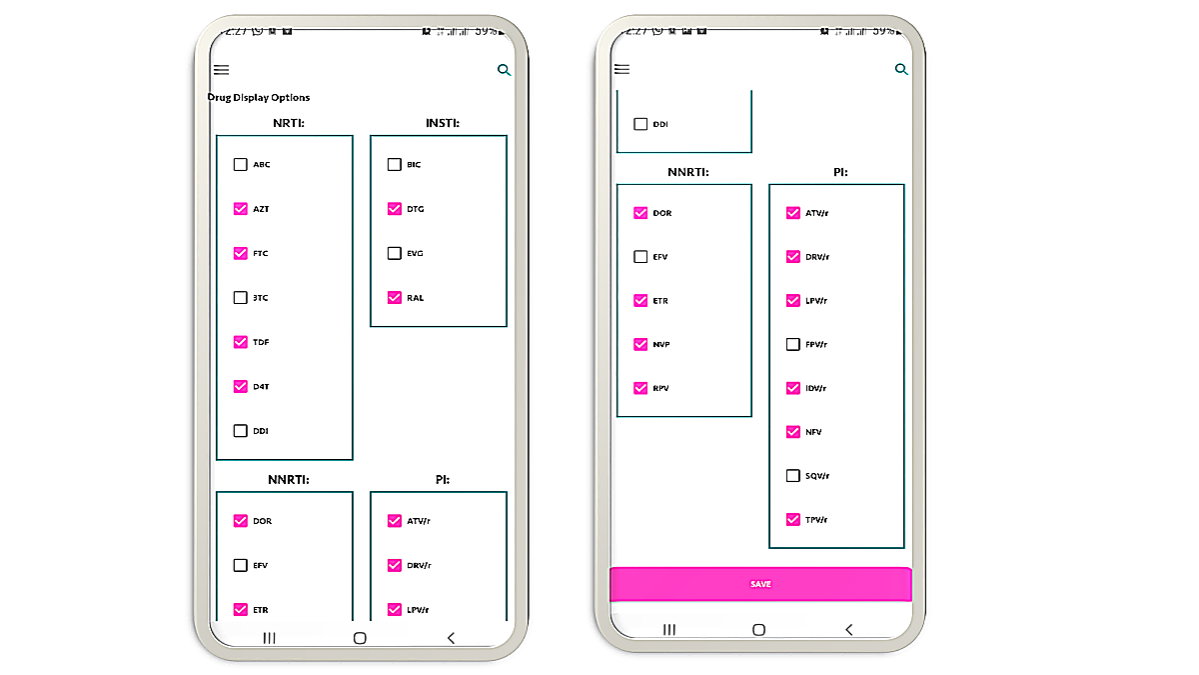
Antiretroviral Display Options: Displaying all current ARVs and categorizes them as Nucleoside reverse transcriptase inhibitors (NRTIs), Non-nucleoside reverse transcriptase inhibitors (NNRTIs), Integrase Strand Transfer Inhibitor (INSTIs) and Protease Inhibitor (PI).

**Figure 10:**
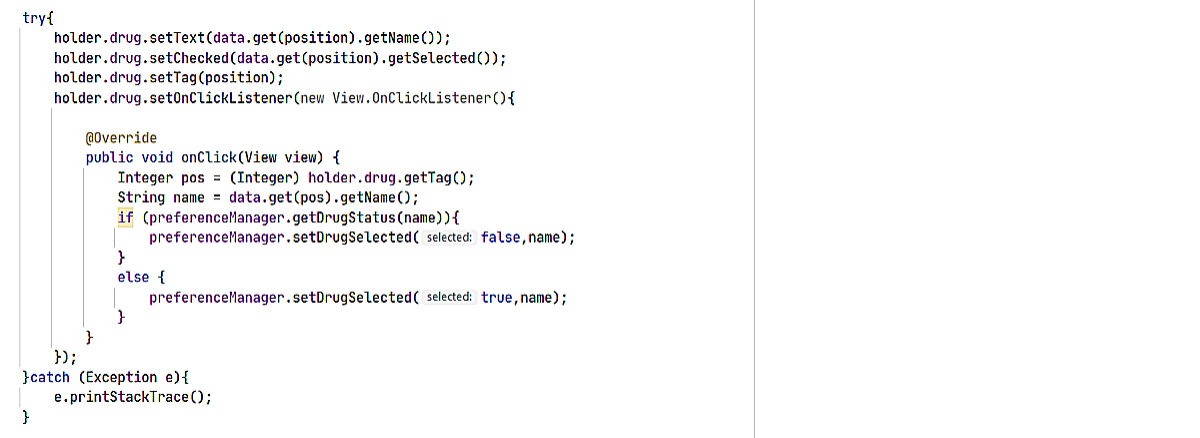
ARV Drug Selection code by category. Set of back-end computer related logical instructions of selection of different ARV drugs available for use by various HIV Patients.

**Figure 11:**
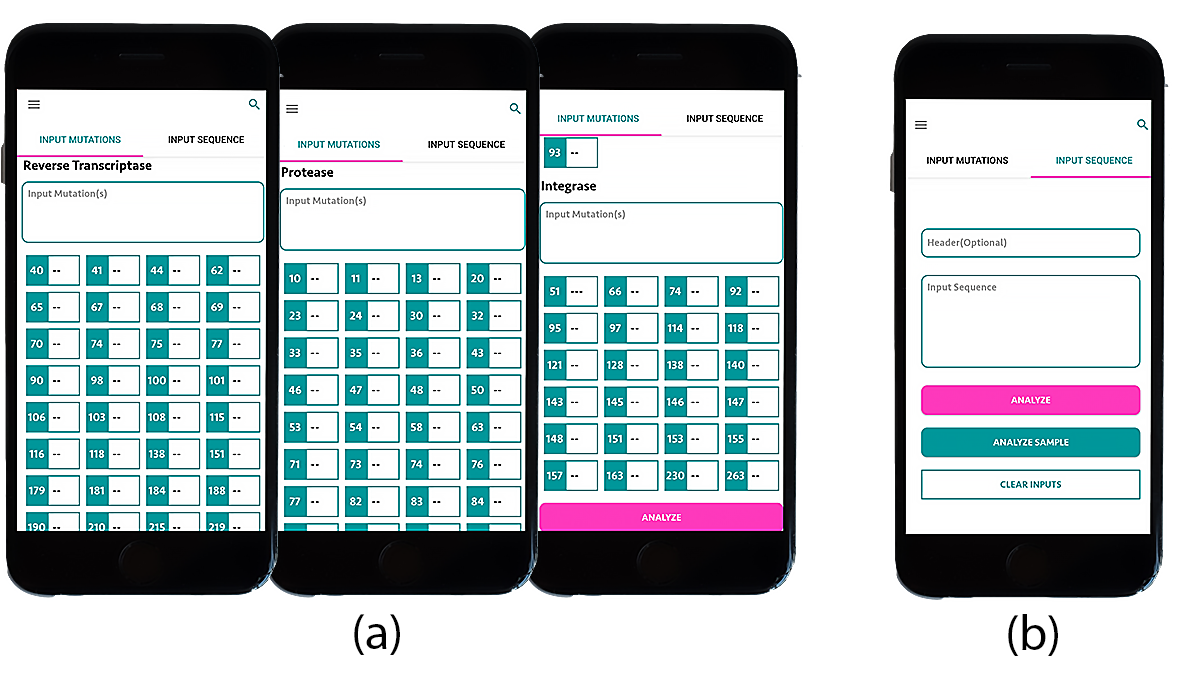
ARVPredictor. (a) Major HIV-1 drug resistance mutation list. (b) Sequence entry option window.

ARVPredictor is currently distributed freely through the Google Play Store and App Store (Apple Inc.), with basic rules requiring users to create an account and provide very limited personal details such as email addresses, passwords, and preferred telephone numbers. Different user-friendly interfaces viewable through the usage of the app are shown in Figures 7-11. The source code for ARVPredictor is available under the MIT permissive license in a GitHub repository [41].

### User Friendly Interfaces

#### User Registration and User login

First-time users of the app require registration into the system. This process helps prevent unauthorized access and use of the app. An SMS text message alert is then sent through the given phone number with an activation code. System registration can occur in 2 different ways: through the app itself (Figure 7) or by the administrator remotely on request. Figure 8 shows the respective back end code used in developing this registration process. Only authenticated and valid email addresses/phone numbers are allowed for registration.

#### Antiretroviral Drug Options/Input Window

Upon successful registration and verification, the next button opens the known and common antiretroviral drug display window (Figure 9). Some antiretroviral drugs will be premarked by default, but checkboxes can be used to add or remove any drug. A “save” button at the bottom of the phone screen allows one to save and use his/her final choice. Figure 10 illustrates the back end code used in the development of this app’s preferred drug selection process.

#### HIV Mutation Selection Window

The next screen displays 2 options of the input type for analysis: point mutations or sets of sequences (Figure 11). The first group is a list of all the registered major HIV-1 drug-resistance mutations as per WHO 2009 data [23]. Three mutation categories, namely, reverse transcriptase, protease, and integrase, can be selected from a scroll down window and analyzed at the bottom of the screen. The second part of this figure is a sequence input window. It provides an option to either upload a set of sequences from a separate source or manually key them in.

#### ARVPredictor Mutation Analysis Results Window

**Figure 12:**
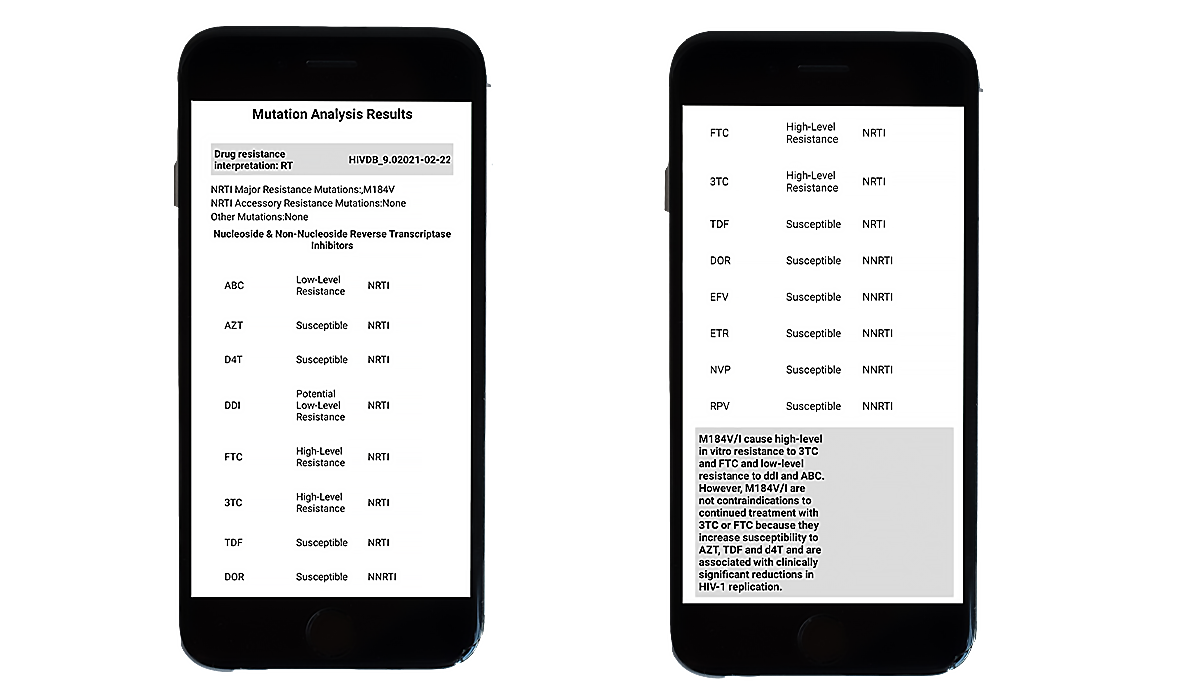
ARVPredictor: Mutation Analysis Results.

**Figure 13:**
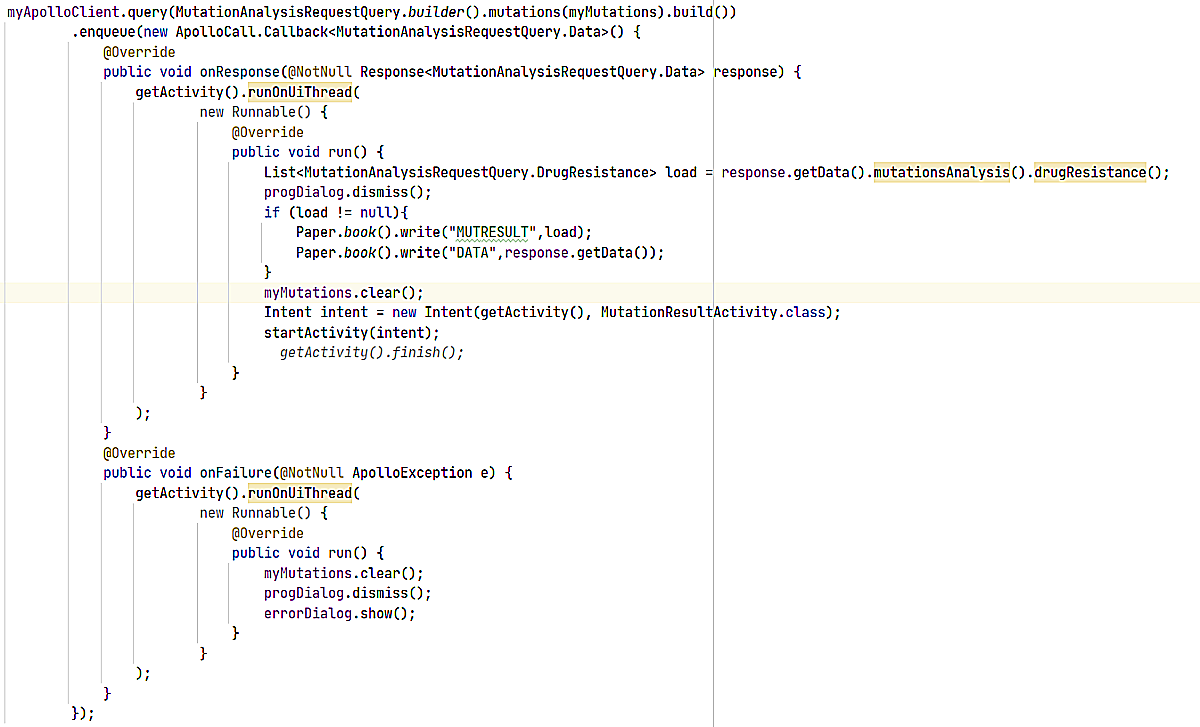
ARVPredictor Mutation Analysis Code.

**Figure 14:**
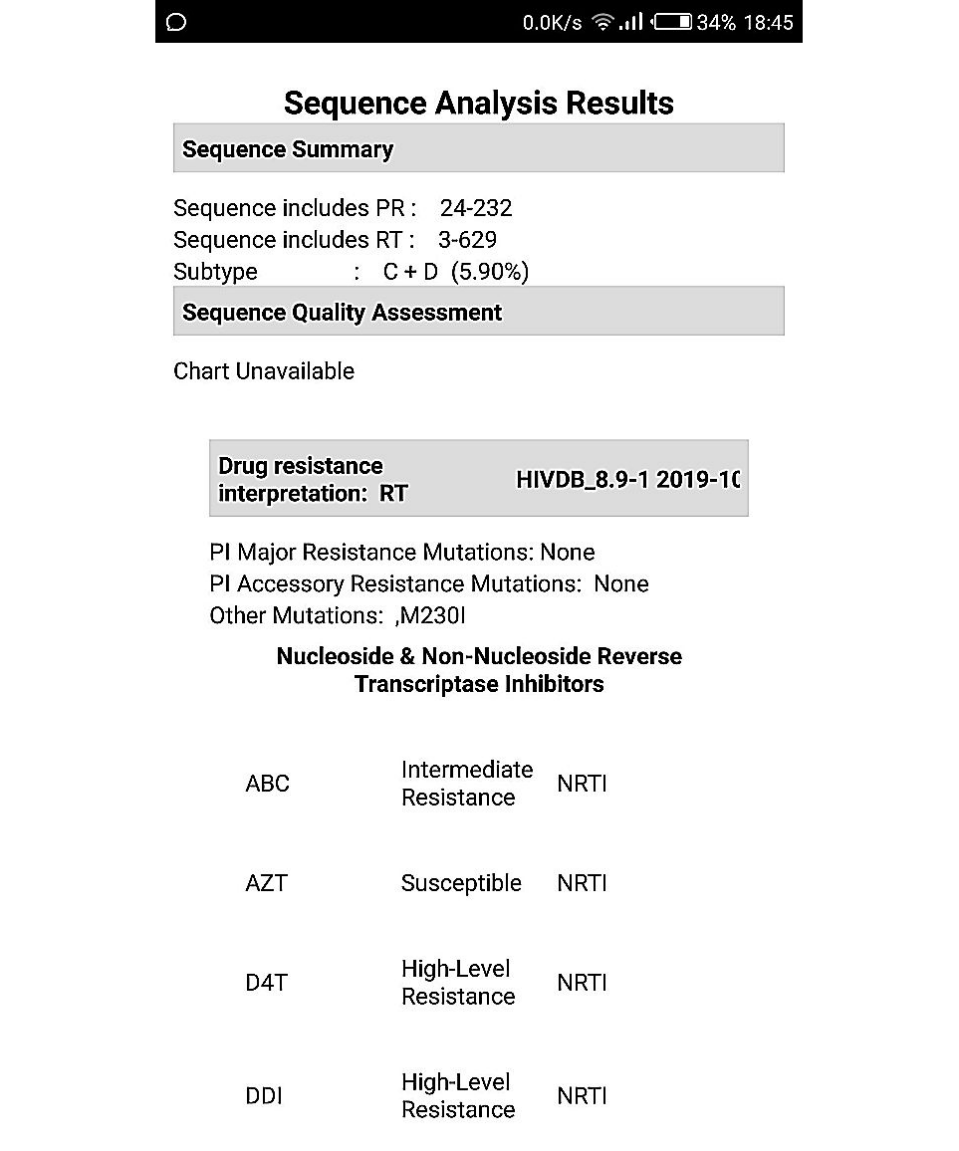
ARVPredictor: Sequence Analysis Results.

**Figure 15:**
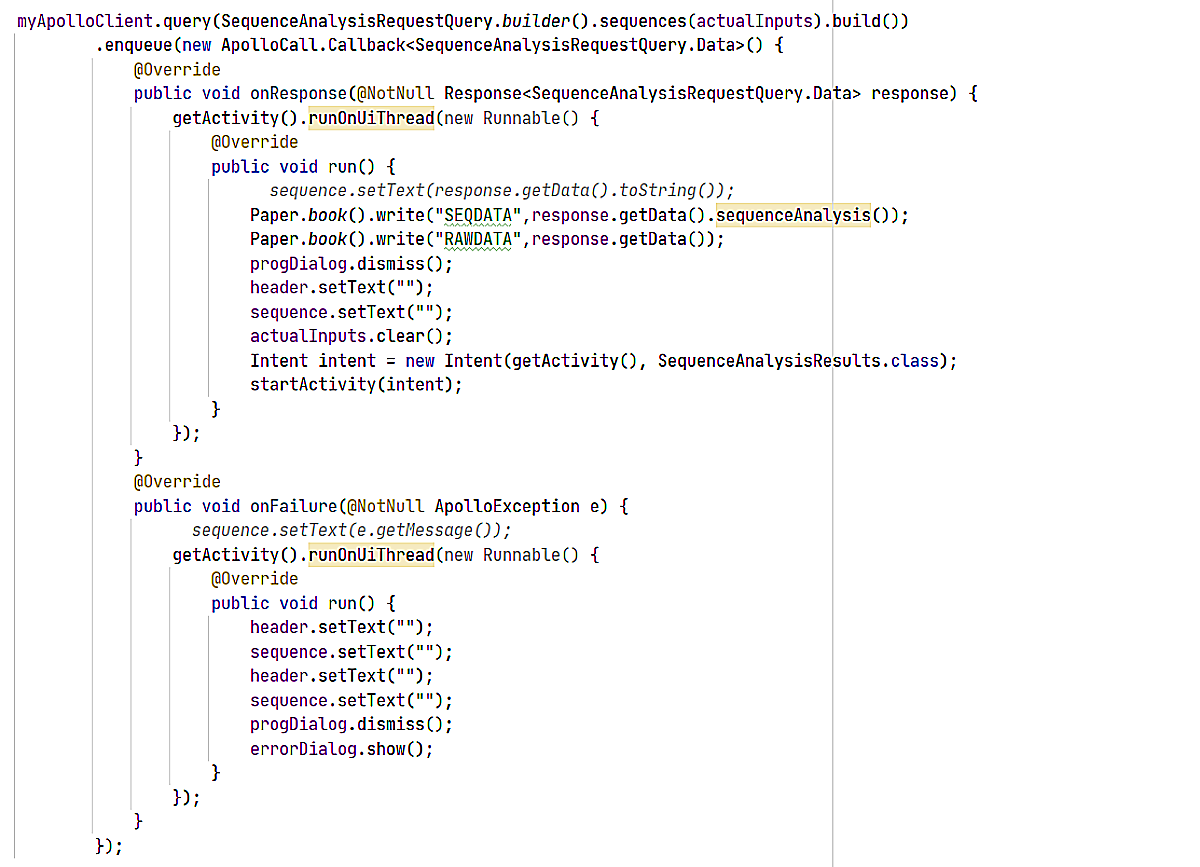
ARVPredictor sequence analysis code.

The results are analyzed and sent back in comparison to the latest version of the Stanford HIV Database. The output is packaged in terms of the region targeted, for example, protease, reverse transcriptase, and integrase, and preferred antiretrovirals are listed alongside. Output of a mutation analysis is shown in Figure 12. The development code for displaying the mutation analysis output in this app is shown in Figure 13. Figures 14 and 15 display the analyzed sequences and respective back end development codes, respectively.

## Discussion

### Principal Findings

Timely and accurate assistance to health care providers in the management of patients with HIV has been a challenge, especially in resource-limited environments. We presume that with the introduction of ARVPredictor, the much needed solution to health care providers at the point of care will be achieved. The planning and choice of tools used in the development of this app were done with the expected work environment and data output in mind. The choice supported heavy and quick analysis of the mutations or sequences to be uploaded by the health care provider. The overall aim is to provide a solution that offers accurate and easily accessible management strategy to HIV health care providers at their fingertips with a short turnaround time. A great effort was made to minimize the expected input variables for accuracy and time management. To achieve this, the mutation screen presents a dropdown and choice-enabled entries, which include a complete list of all currently documented mutations. The sequence analysis window allows keying in, pasting, and uploading from a remote file.

The app, which is now freely downloadable from Google Play Store or App Store and enlisted as ARVPredictor, is expected to be free for use and technically supported by developers. Its security is enhanced through controlled and authenticated log-in process, while critical and back end data are only accessible to authorized individuals.

### Conclusions

In evaluating the functionality of the ARVPredictor in comparison to the replicated Stanford HIV Database in this study, there is strong evidence that several benefits, including but not limited to concurrence, convenience, and simplicity, are realized. ARVPredictor can therefore be used to determine HIV-1 drug-resistance mutations in the HIV *pol* gene with ease and convenience in mobile devices. The app equally has the advantages of high-speed data networks and smartphone accessibility. It therefore adds to other available and upcoming mHealth interventions in the area of HIV and antiretroviral use among different health care providers. Mobile technology has enabled faster and efficient communication among health care service providers and their patients. However, additional benefits, such as improved diagnosis, accuracy, and better coordination, will still contribute highly to the biomedical field.

## Appendix

Multimedia Appendix 1 ARVPredictor User Guide.

Multimedia Appendix 2 ARVPredictor Test Performance Sequences (Accession Numbers: KX505314-KX505372 and MK588680-MK588752) using Stanford HIV database as the gold standard.

Multimedia Appendix 3 Test Performance Output: Performance results of ARVPredictor using the Stanford HIV database as the gold standard in identification of HIV subtypes and mutations.

## Abbreviations

HIVdb: HIV Drug Resistance Database
HIVDR: HIV drug resistance
ICT: information and communications technology
IDE: Integrated Development Environment
NAHCC: National Advanced HIV Clinical Course
NCBI: National Center for Biotechnology Information
NNRTI: non-nucleoside reverse transcriptase inhibitors
NRTI: nucleoside reverse transcriptase inhibitor
RAD: rapid application development
UNAIDS: United Nations Programme on HIV/AIDS

## References

[1] Griffin H (2013). Has Mobile Phone Technology Had an Impact on the Quality of Life in the Developing World?. IJARBSS.

[2] Quade M, Leimstoll U (2015). Mobile business with smartphones and tablets: effects of mobile devices in SMEs. BLED 2015 Proceedings.

[3] Mosa Abu Saleh Mohammad, Yoo Illhoi, Sheets Lincoln (2012). A systematic review of healthcare applications for smartphones. BMC Med Inform Decis Mak.

[4] Lippi G, Plebani M (2011). Laboratory applications for smartphones: risk or opportunity?. Clin Biochem.

[5] mobiThinking (2014). Global mobile statistics 2014 Part B: Mobile Web; mobile broadband penetration; 3G/4G subscribers and networks; mobile search. mobiForge.

[6] Divall P, Camosso-Stefinovic J, Baker R (2013). The use of personal digital assistants in clinical decision making by health care professionals: a systematic review. Health Informatics J.

[7] Miralles C, Granell Carlos, Díaz-Sanahuja Laura, Van Woensel William, Bretón-López Juana, Mira Adriana, Castilla Diana, Casteleyn Sven (2020). Smartphone Apps for the Treatment of Mental Disorders: Systematic Review. JMIR Mhealth Uhealth.

[8] Steinkamp JM, Goldblatt Nathaniel, Borodovsky Jacob T, LaVertu Amy, Kronish Ian M, Marsch Lisa A, Schuman-Olivier Zev (2019). Technological Interventions for Medication Adherence in Adult Mental Health and Substance Use Disorders: A Systematic Review. JMIR Ment Health.

[9] Reins JA, Boß Leif, Lehr Dirk, Berking Matthias, Ebert David Daniel (2019). The more I got, the less I need? Efficacy of Internet-based guided self-help compared to online psychoeducation for major depressive disorder. J Affect Disord.

[10] Killikelly Z, He Zhimin, Reeder Clare, Wykes Til (2017). Improving Adherence to Web-Based and Mobile Technologies for People With Psychosis: Systematic Review of New Potential Predictors of Adherence. JMIR Mhealth Uhealth.

[11] Fiol-DeRoque MA, Serrano-Ripoll Maria Jesús, Jiménez Rafael, Zamanillo-Campos Rocío, Yáñez-Juan Aina María, Bennasar-Veny Miquel, Leiva Alfonso, Gervilla Elena, García-Buades M Esther, García-Toro Mauro, Alonso-Coello Pablo, Pastor-Moreno Guadalupe, Ruiz-Pérez Isabel, Sitges Carolina, García-Campayo Javier, Llobera-Cánaves Joan, Ricci-Cabello Ignacio (2021). A Mobile Phone-Based Intervention to Reduce Mental Health Problems in Health Care Workers During the COVID-19 Pandemic (PsyCovidApp): Randomized Controlled Trial. JMIR Mhealth Uhealth.

[12] Lee H (2016). Future of the Smartphone for Patients and Healthcare Providers. Healthc Inform Res.

[13] (2020). About HIV and AIDS.

[14] (2019). HIV Drug Resistance. Avert.

[15] AIDSinfo.

[16] Tchiakpe R, Keke Rene K, Vidal Nicole, Ahoussinou Clément, Sekpe Olga, Dagba Hermione G, Gbaguidi Eric, Tonoukouen Conrad, Afangnihoun Aldric, Bachabi Moussa, Gangbo Flore A, Diop-Ndiaye Halimatou, Toure-Kane Coumba (2020). Moderate rate of transmitted resistance mutations to antiretrovirals and genetic diversity in newly HIV-1 patients diagnosed in Benin. BMC Res Notes.

[17] (2014). 90-90-90: An Ambitious Treatment Target to Help End the Aids Epidemic. UNAIDS.

[18] Hirsch MS, Brun-Vézinet F, D'Aquila RT, Hammer SM, Johnson VA, Kuritzkes DR, Loveday C, Mellors JW, Clotet B, Conway B, Demeter LM, Vella S, Jacobsen DM, Richman DD (2000). Antiretroviral drug resistance testing in adult HIV-1 infection: recommendations of an International AIDS Society-USA Panel. JAMA.

[19] Ehrenkranz Peter D, Baptiste Solange L, Bygrave Helen, Ellman Tom, Doi Naoko, Grimsrud Anna, Jahn Andreas, Kalua Thokozani, Nyirenda Rose Kolola, Odo Michael O, Ondoa Pascale, Vojnov Lara, Holmes Charles B (2019). The missed potential of CD4 and viral load testing to improve clinical outcomes for people living with HIV in lower-resource settings. PLoS Med.

[20] (2021). ARVPredictor - Apps on Google Play.

[21] Rhee Soo-Yon, Gonzales Matthew J, Kantor Rami, Betts Bradley J, Ravela Jaideep, Shafer Robert W (2003). Human immunodeficiency virus reverse transcriptase and protease sequence database. Nucleic Acids Res.

[22] Stanford Database (HIVdb).

[23] Bennett DE, Camacho RJ, Otelea D, Kuritzkes DR, Fleury H, Kiuchi M, Heneine W, Kantor R, Jordan MR, Schapiro JM, Vandamme A, Sandstrom P, Boucher CAB, van de Vijver D, Rhee S, Liu TF, Pillay D, Shafer RW (2009). Drug resistance mutations for surveillance of transmitted HIV-1 drug-resistance: 2009 update. PLoS One.

[24] Choudhury A (2011). Waterfall Model/SDLC.

[25] Beynon-Davies P, Carne C, Mackay H, Tudhope D (2017). Rapid application development (RAD): an empirical review. European Journal of Information Systems.

[26] Pawar RP (2015). A comparative study of Agile software development methodology and traditional waterfall model. IOSR Journal of Computer Engineering.

[27] Chung I, Ko I (2015). Data-Sharing Method for Multi-Smart Devices at Close Range. Mobile Information Systems.

[28] Paetsch F, Eberlein A, Maurer F (2003). Requirements engineering and agile software development.

[29] Gomaa H, Mason G (2011). Software Modeling and Design: UML, Use Cases, Patterns, and Software Architectures.

[30] Smyth N (2020). Android Studio 4.1 Development Essentials: Java Edition.

[31] Potts D (1996). Java Programming Language Handbook (First Edition).

[32] (2017). IntelliJ IDEA Handbook.

[33] (2003). Java Programming Language - An Overview.

[34] Dusch V (2012). Nginx - the webserver you might actually like. https://www.researchgate.net/publication/231400849_Nginx_-_The_Webserver_you_might_actually_like/link/5a71c5300f7e9ba2e1cc43a4/download.

[35] (2011). DigitalOcean - The developer cloud.

[36] (1995). MySQL.

[37] (2021). GraphQL (Microservices) Architecture by Apollo. ITNEXT.

[38] Drohan D (2017). Android Retrofit.

[39] McHugh Ml (2012). Interrater reliability: the kappa statistic. Biochem Med.

[40] Macharia VM, Ngugi C, Lihana R, Ngayo MO (2016). Transmitted HIV-1 drug resistance and the role of herpes simplex virus-2 coinfection among fishermen along the shores of Lake Victoria, Kisumu, Kenya. J HIV Retrovirus.

[41] (2021). ARV_Predictor_App_OngadiBA. GitHub.

