# Supplementary material for: An Android-Based Mobile App (ARVPredictor) for the Detection of HIV Drug-Resistance Mutations and Treatment at the Point of Care: Development Study
Source: JMIR Form Res. 2022 Feb 2;6(2):e26891. doi: 10.2196/26891 (PMC8851341; doi:10.2196/26891)
Supplement: Multimedia Appendix 1 [file formative_v6i2e26891_app1.pdf]

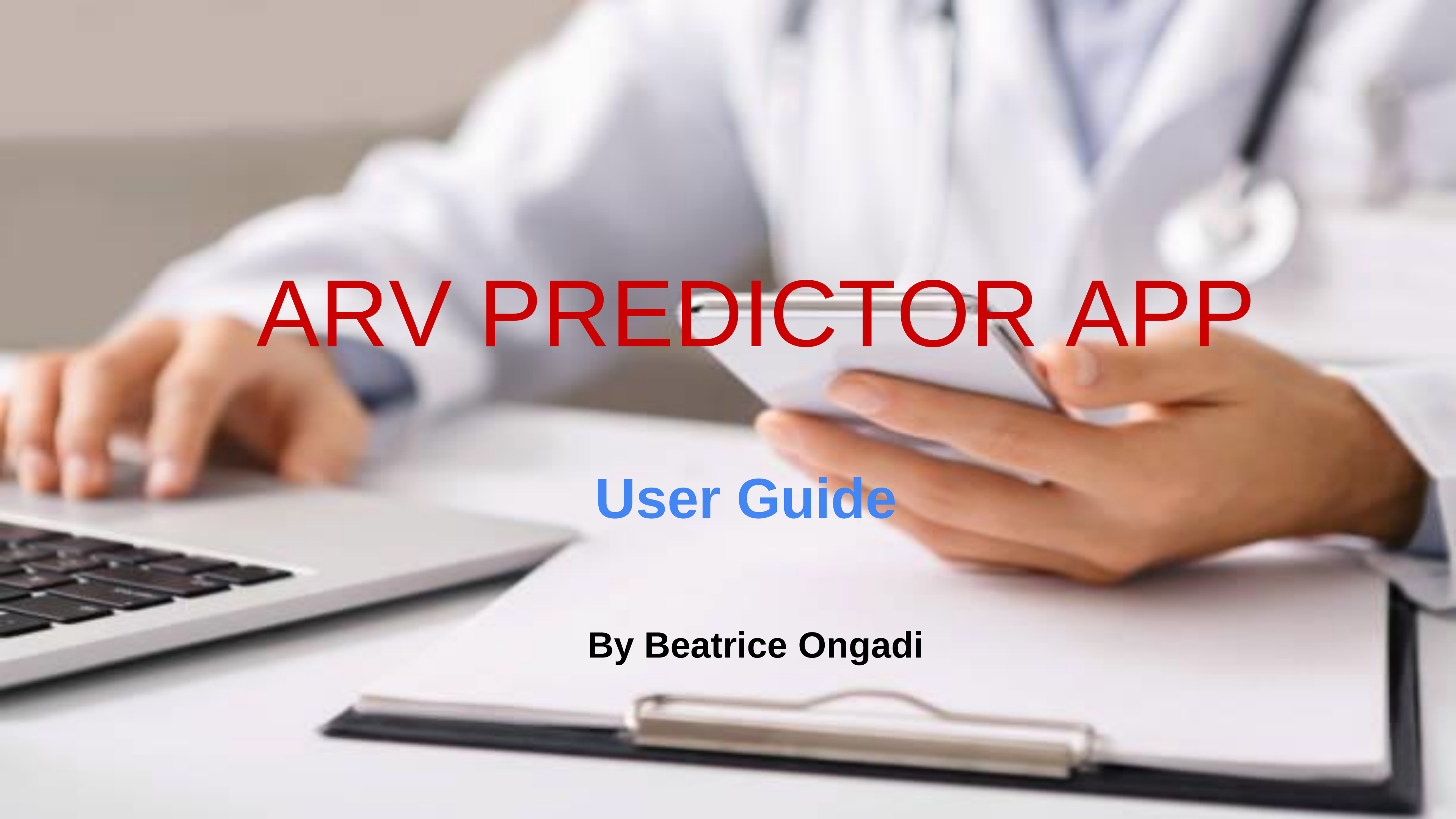

ARV PREDICTOR APP

User Guide

By Beatrice Ongadi

Intro Screens

The introduction screen explains more about the app and what it does. These can always be skipped by swiping right.

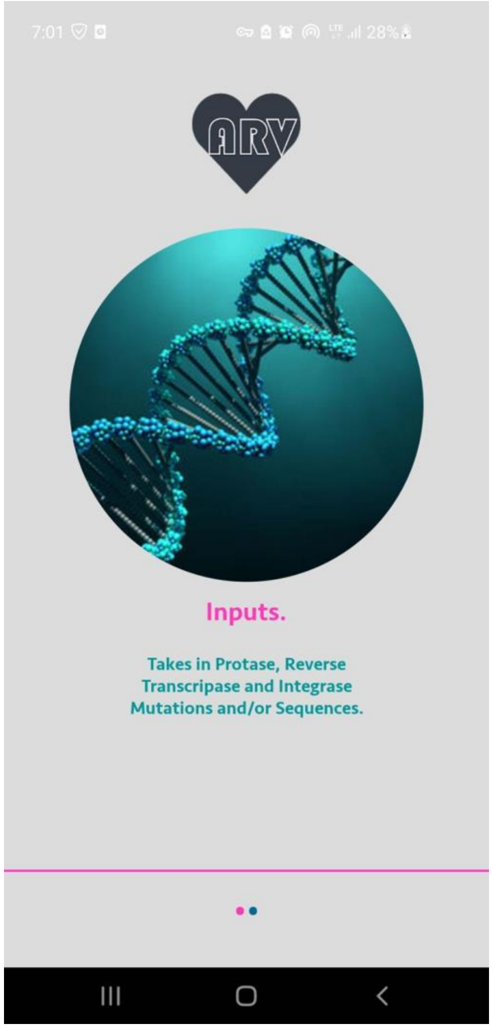

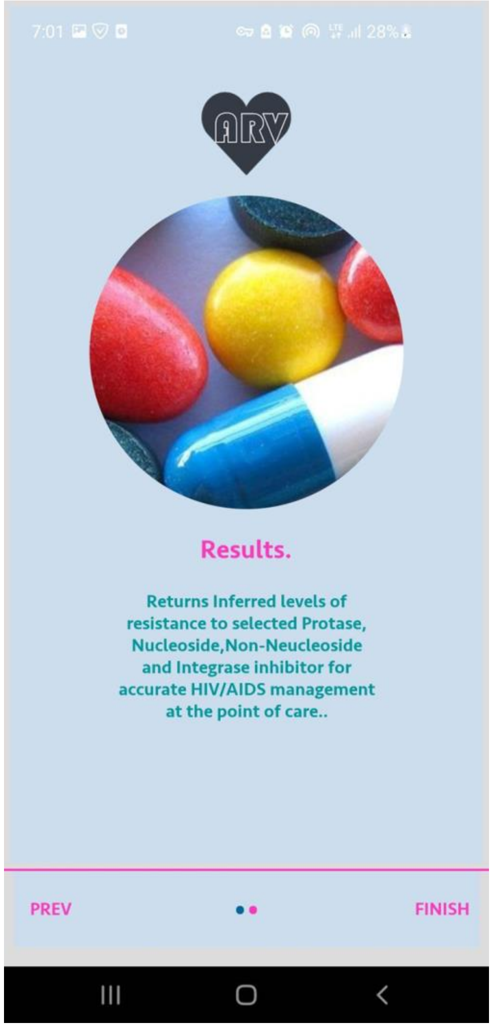

Login Page

On this page, you the user is to provide the phone number they registered with and their password before clicking the Login button. However if the user is not registered, they should click the register button for registration page.

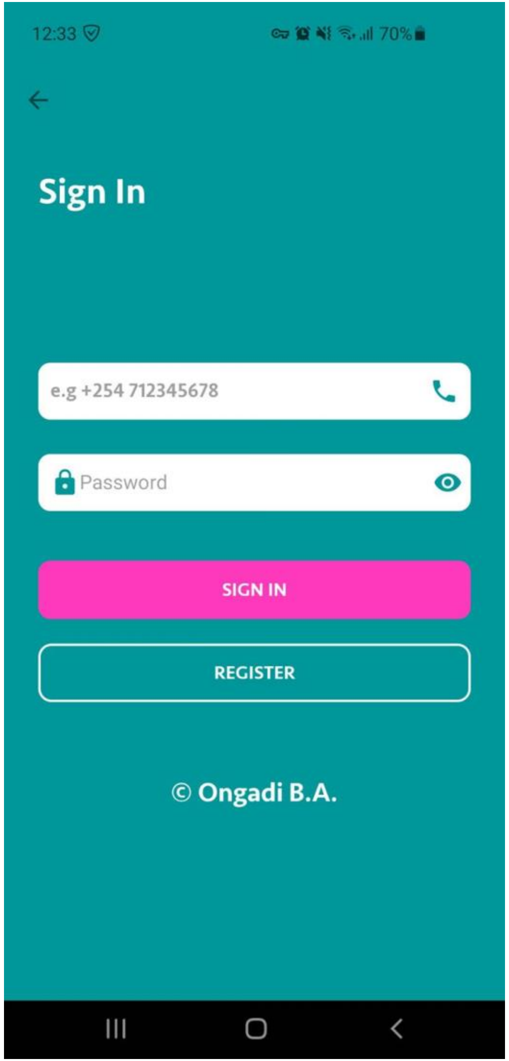

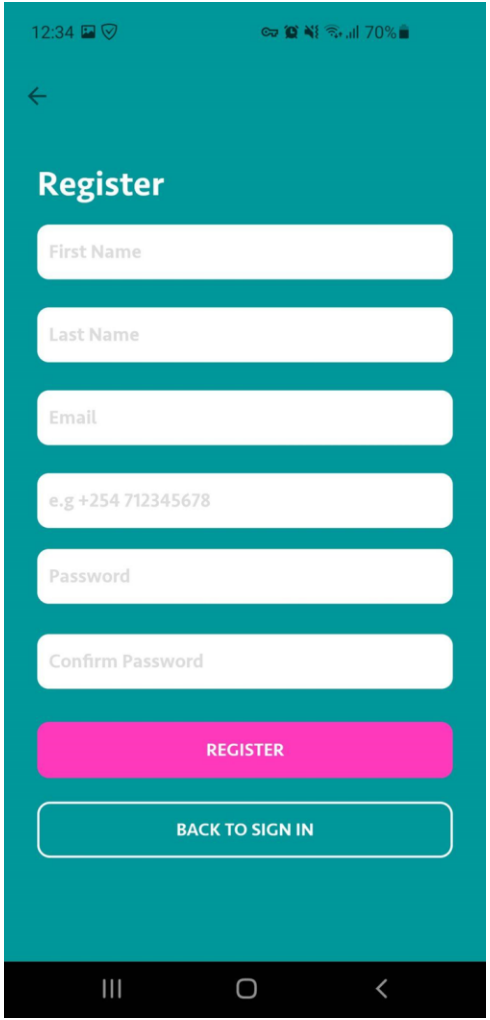

Registration Page

On this page., the user provides they first and second names, theri email,phone number and password. They then click register to initiate the registration process.

Phone Number Verification

In order to be sure about the phone number provided, we send a verification code through SMS. this code should be provided on this page for verification purposes.

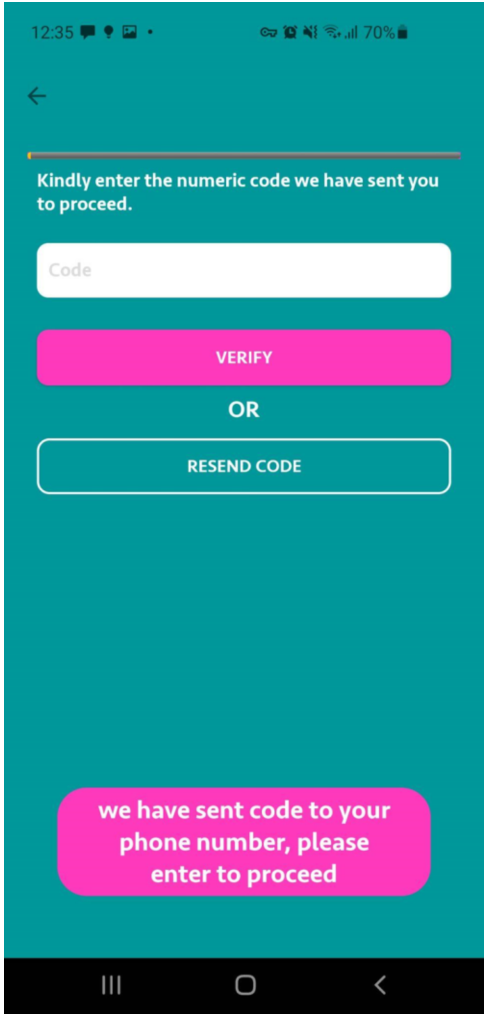

Drug Selection Page

On this page, the user selects the drug varieties that they want to be part of the results during mutation or sequence analysis. It is advisable to select all the available drugs.

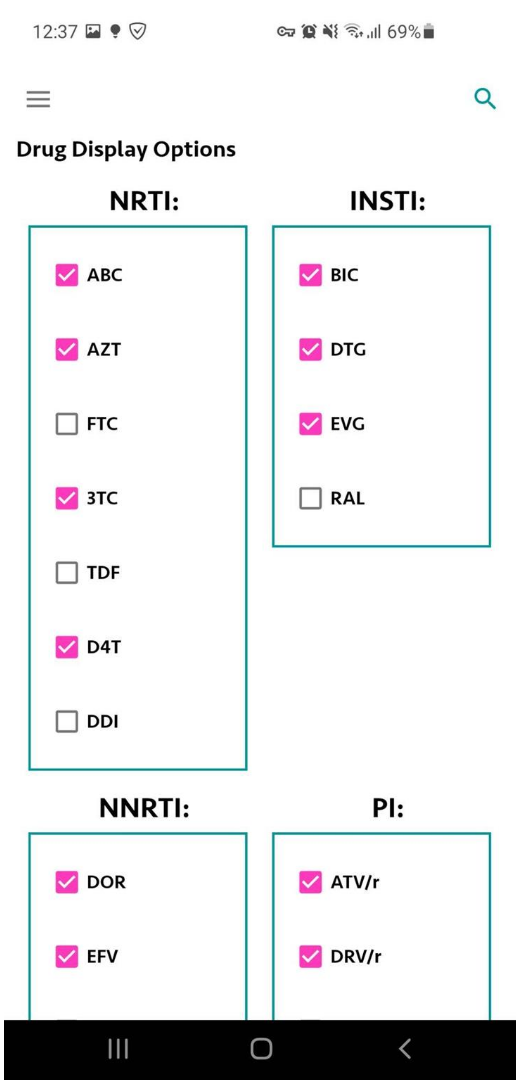

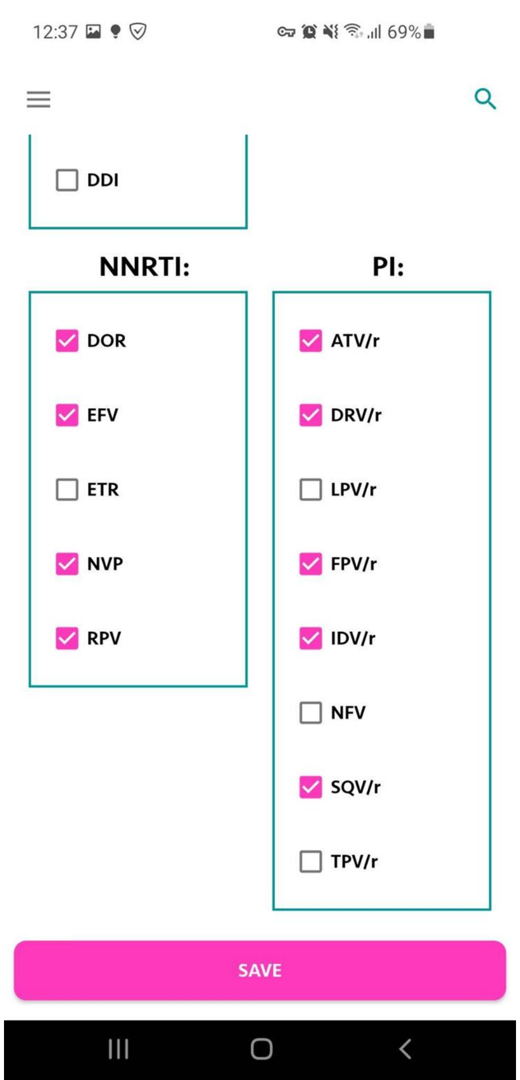

Mutation Selection Page

On this screen, the user is able to select the various mutations to analyze. There is the analyze button to initiate analysis.

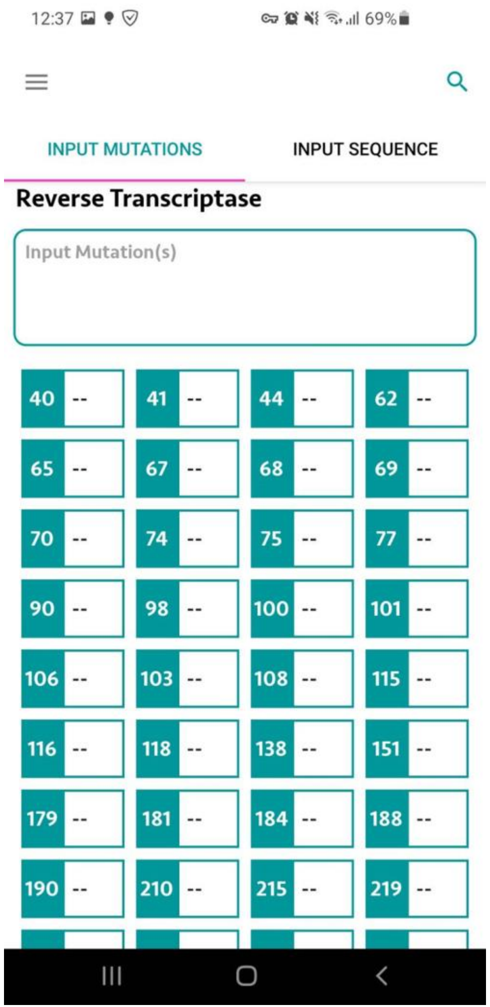

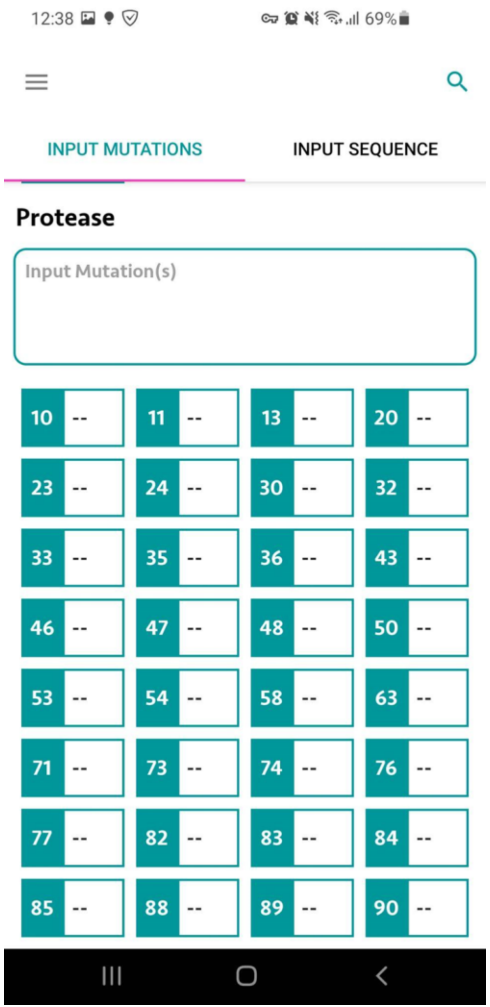

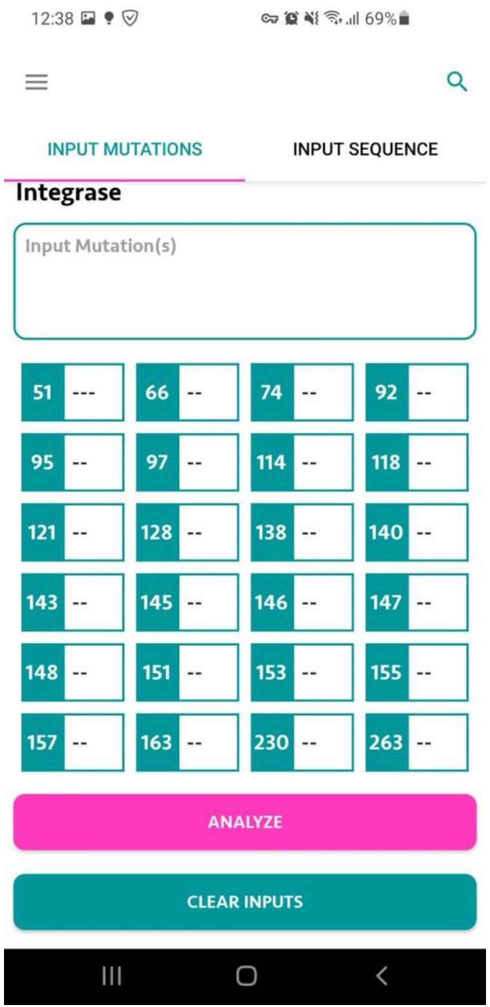

Mutation Analysis Result Page

On this screen, the user is set to view the results from analysis of particular mutation sets. These are very comprehensive results and are produced depending on the number of mutations provided.

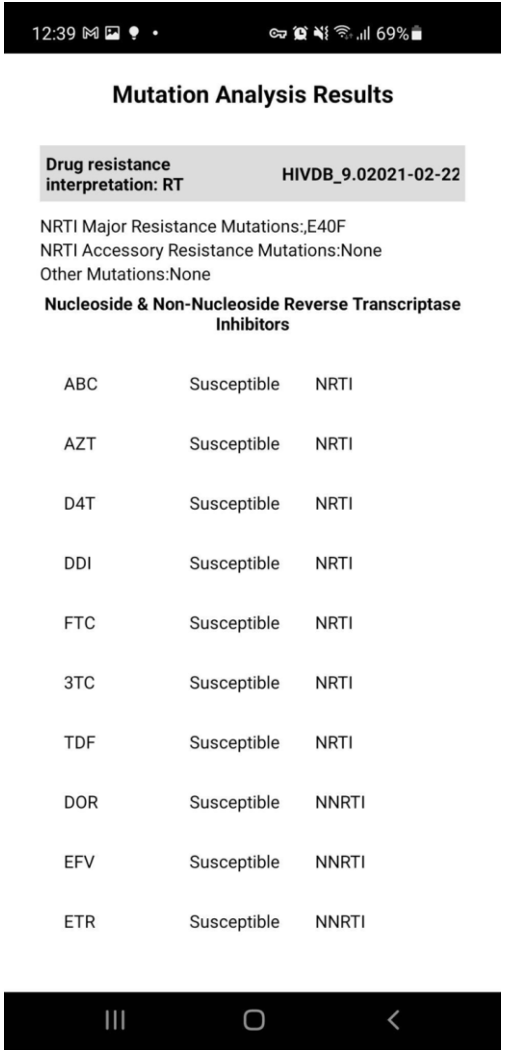

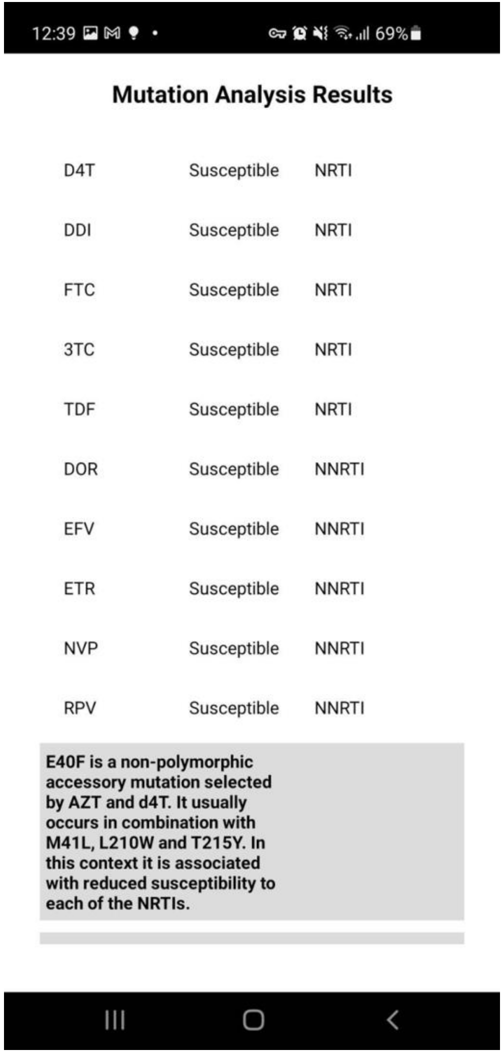

Sequence Analysis Page

This screen expects the sequence string from the user and a header parameter. To ensure no interference, the string should be copied from a separate file and pasted on the provided text area. The headers define extra parameters needed for the analysis.

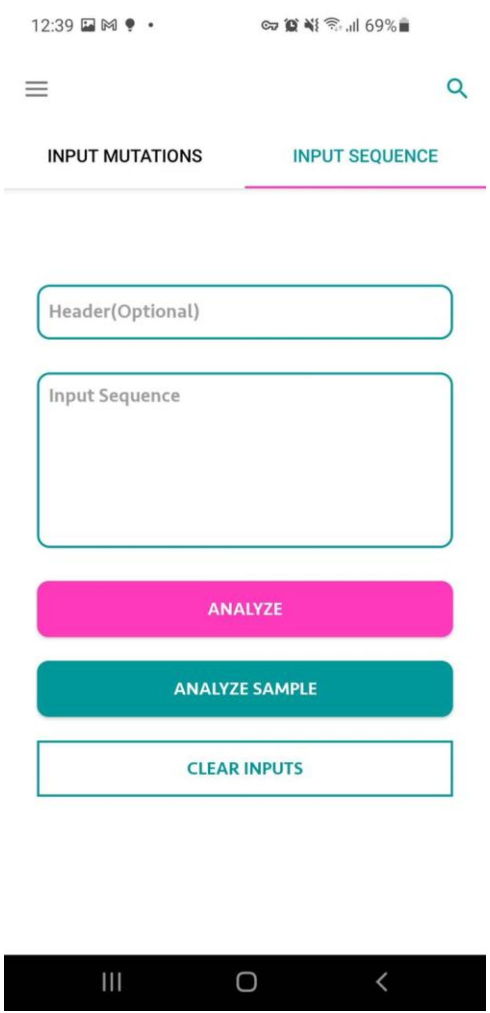

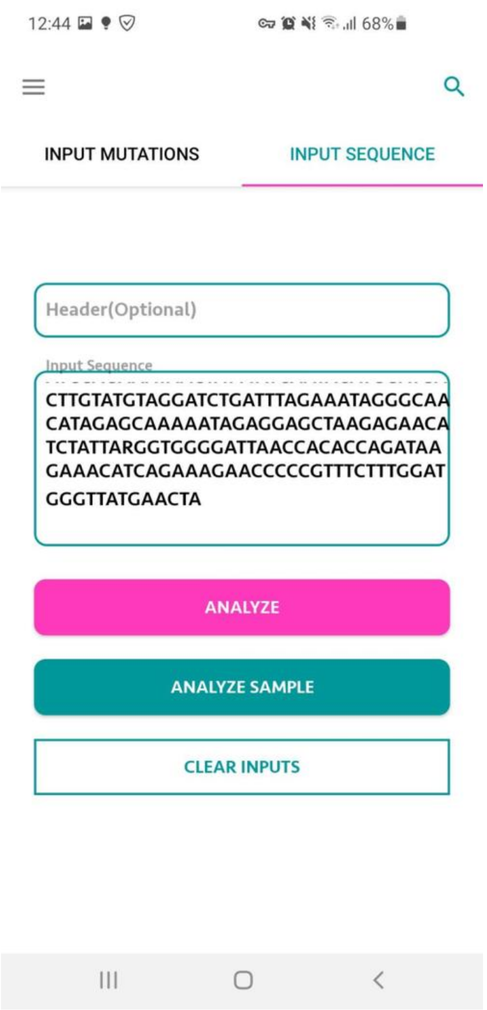

Sequence Analysis Result Page

On this screen, the user will be able to view the results of their sequence analysis. The results are as well comprehensive and are displayed according to the number of sequences selected.

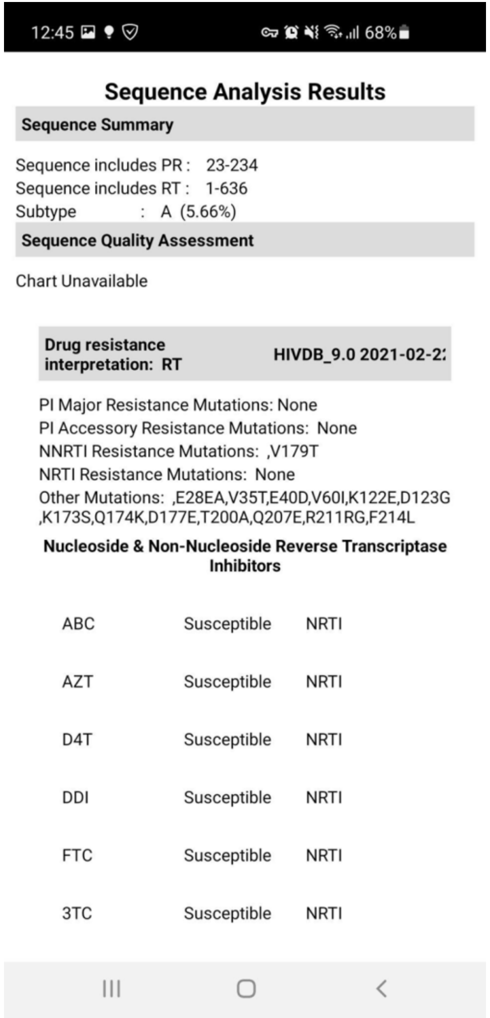

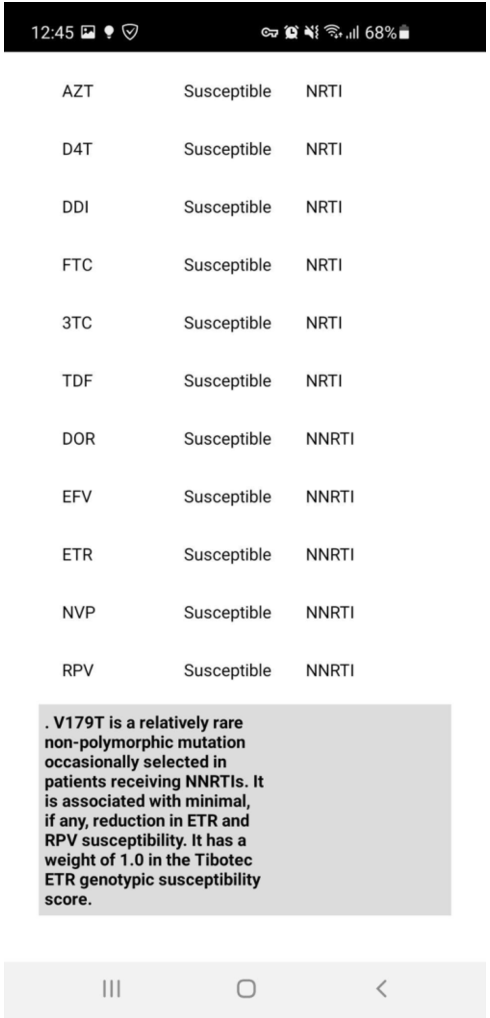

Thank You.
