# Supplementary material for: An Android-Based Mobile App (ARVPredictor) for the Detection of HIV Drug-Resistance Mutations and Treatment at the Point of Care: Development Study
Source: JMIR Form Res. 2022 Feb 2;6(2):e26891. doi: 10.2196/26891 (PMC8851341; doi:10.2196/26891)
Supplement: Multimedia Appendix 2 [file formative_v6i2e26891_app2.docx]

**Table 2**. One hundred test performance sequences for ©ARVPredictor using Stanford database as the gold standard to identify HIV subtypes and mutations.

| PID | SEQ |
| --- | --- |
| 001 | CAATGGCCATTGACAGMAGAAAAAATAAAAGCATTAACAGAAATTTGCACAGATATGGAAAAGGAAGGAAAAATTTCAAAAATTGGGCCTGAAAATCCATACAATACTCCAATATTTGCTATAAAGAAAAAAGACAGTACTAAATGGAGGAAATTAGTAGATTTCAGGGAGCTCAATAAAAGAACACAAGACTTTTGGGAAGTTCAATTAGGGATACCGCATCCAGCGGGCTTAAAAAAGAAGAAATCAGTAACAGTACTAGATGTGGGGGACGCATATTTTTCAGTTCCTTTAGATGAAGGCTTTAGGAAATATACTGCGTTCACCATACCTAGTATAAACAATGAGACACCAGGAATCAGATATCAGTATAATGTGCTCCCACAGGGATGGAAAGGATCACCAGCAATATTCCAGAGTAGYATGACAAAAATCTTAGAGCCCTTCAGATCAAAAAATCCAGAAATAACTATTTATCAATACATGGATGACTTGTATGTAGGATCTGATTTAGAAATAGGGCAACATAGAGCAAAAATAGAGGAGCTAAGAGAACATCTATTARGGTGGGGATTAACCACACCAGATAAGAAACATCAGAAAGAACCCCCGTTTCTTTGGATGGGTTATGAACTA  SUBTYPE A:SUS |
| 002 | AAACAATGGCCATTGACAGAAGAAAAAATAAAAGCATTAACAGAAATTTGTATGGAAATGGAGAAGGAAGGAAAAATTTCAAAAATTGGGCCTGAAAATCCATACAATACACCAATATTTGCAATAAAGAAAAAGGATAGCACTAAATGGAGGAAATTAGTAGATTTCAGAGAGCTCAATAAAAGAACACAAGACTTTTGGGAAGTTCAGCTAGGAATACCGCATCCAGCGGGTCTAAAAAAGAAAAAATCAGTAACAGTACTAGATGTGGGGGACGCATATTTTTCAGTTCCTTTACATGAAGGCTTTARAAAATATACTGCATTCACCATACCTAGTACAAACAATGAGACACCAGGAATCAGATATCAGTACAATGTGCTTCCACAGGGATGGAAAGGATCACCATCAATATTCCAGAGTAGCATGATAAAAATTTTAGAACCTTTCAGATCAAAAAATCCAGAAATAATTATCTATCAATATATGGATGACTTGTATGTAGGATCTGATTTAGAAATAGAGCAACATCGAGCAAAAATAGAAGAGTTGAGAGCTCATCTATTGAGCTGGGGATTTACTACACCAGACAAAAAGCATCAGAAAGAACCTCCATTCCTTTGGATGGGWTATGAACTA  SUBTYPE A:SUS |
| 003 | AAACAATGGCCATTGACAGAAGAAAAAATAAAAGCATTAACAGAAATTTGTATGGAAATGGAGAAGGAAGGAAAAATTTCAAAAATTGGGCCTGAAAATCCATACAATACACCAATATTTGCAATAAAGAAAAAGGATAGCACTAAATGGAGGAAATTAGTAGATTTCAGAGAGCTCAATAAAAGAACACAAGACTTTTGGGAAGTTCAGCTAGGAATACCGCATCCAGCGGGTCTAAAAAAGAAAAAATCAGTAACAGTACTAGATGTGGGGGACGCATATTTTTCAGTTCCTTTACATGAAGGCTTTAGAAAATATACTGCATTCACCATACCTAGTACAAACAATGAGACACCAGGAATCAGATATCAGTACAATGTGCTTCCACAGGGATGGAAAGGATCACCATCGATATTCCAGAGTAGCATGATAAAAATTTTAGAACCTTTCAGATCAAAAAATCCAGAAATAATTATCTATCAATATATGGATGACTTGTATGTAGGATCTGATTTAGAAATAGAGCAACATCGAGCAAAAATAGAAGAGTTGAGAGCTCATCTATTGAGCTGGGGATTTACTACACCAGACAAAAAGCATCAGAAAGAACCTCCATTCCTTTGGATGGGTTATGAACTA  SUBTYPE A:SUS |
| 004 | GTTAAACAATGGCCATTGACAGAAGAAAAAATAAAAGCATTAACAGAAATTTGTACAGAAATGGAAAAGGAAGGAAAAATTTCAAAAATTGGACCTGAAAATCCATACAATACTCCAATATTTGCTATAAAGAAAAAGGACAGCACTAAATGGAGAAAATTAGTAGATTTCAGAGAGCTCAATAAAAGAACTCAAGATTTTTGGGAAGTTCAATTAGGAATACCGCATCCAGCGGGCCTAAAAAAGAAAAAATCAGTAACAGTACTGGATGTGGGGGATGCATATTTTTCAGTACCTTTAGATGAAAGCTTTAGAAAATATACTGCATTCACCATACCTAGTACAAACAATGAGACACCAGGAATCAGGTATCAGTACAATGTGCTTCCACAGGGATGGAAAGGGTCACCAGCAATATTCCAGAGTAGCATGACAAAAATCTTAGAGCCCTTTAGATCAAAAAATCCAGAAATAGTTATCTACCAATACATGGATGACTTGTATGTAGGATCTGATTTAGAAATAGGGCAGCATAGAGCAAAAATAGAAGAATTAAGAGCTCATCTGTTGAGCTGGGGATTTACTACCCCMGACAAAAAACATCAGAAAGAACCGCCATTTCTTTGGATGGGTTATGAAC  SUBTYPE A:SUS |
| 005 | AGAAATTTGTACAGATATGGAAAAGGAAGGAAAACTATCAAGGATTGGGCCTGAAAATCCATATAACACTCCAATATTTGCTATAAAGAAAAAAGACAGTACCAAGTGGAGAAAATTAGTAGATTTCAGGGAACTTAATAAGAGAACTCAAGATTTCTGGGAAGTTCAATTAGGAATACCACACCCGGCAGGGCTAAAAAAGAAAAAATCAGTAACAGTACTGGATGTGGGTGATGCCTATTTTTCAGTTCCCTTATGTGAAGAGTTTARAAAATATACTGCATTTACCATACCTAGTATAAACAATGAGACACCAGGAATTAGATATCAGTACAATGTGCTTCCACAGGGATGGAAAGGATCACCAGCAATATTCCAAAGTAGCATGACAAAAATCTTAGAACCCTTTAGAGAACAAAATCCAGAAATAGTTATCTATCAATACATGGATGATTTGTATGTAGGATCTGACTTAGAAATAGGGCAGCATAGAGCAAAAATAGAGGAACTAAGAGAACATCTATTGAGGTGGGGATTTACCACACCAGATAAAAAACATCAGAAAGAACCTCCATTTCTTTGGATGGGTTATGAACTAAA  SUBTYPE D:SUS |
| 006 | CAATGGCCATTGACAGAAGAAAAAATAAAMGCATTAACAGAAATCTGTACAGAAATGGAGGAAGAAGGAAAAATTTCAAAAATTGGGCCTGAAAATCCATACAATACTCCAATATTTGCGATAAAGAAAAAAGATAGCACTAAATGGAGGAAATTAGTAGATTTTAGAGAGCTTAATAAAAGAACTCAAGACTTTTGGGAGGTTCAATTAGGAATACCGCATCCAGCAGGTTTAAAAAAGAAMAAATCAGTAACAGTACTAGATGTGGGGGACGCATATTTTTCAGTTCCTTTAGATGAAGGCTTTAGAAAGTATACAGCGTTCACCATACCTAGTACAAACAATGAGACACCAGGAATCAGGTATCAGTACAATGTGCTTCCACAGGGATGGAAAGGATCACCGGCAATATTCCAAAGTAGCATGACAAAAATCTTAGAGCCCTTTAGAATAAAAAATCCAGACATAATTATCTATCAATACATGGATGACTTATATGTAGGATCTGATTTAGAGATAGGGCAGCATAGAGCAAAAATAGAAGAGTTGAGAGCTCACCTATTGAGCTGGGGATTCACTACACCAGACAAAAAGCATCAGAAAGAACCTCCATTCCTTTGGATGGGTTATGAACTA  SUBTYPE A:NNRTI RES EFV,NFP |
| 007 | AAACAATGGCCATTGACAGAAGAAAAGATAAAAGCATTGACAGAAATTTGTACAGACATGGAAAAGGAAGGAAAAATTTCAAGAATTGGGCCTGAAAATCCATACAATACTCCAATATTTGCTATAAAGAAAAAAGACAGTACTAAGTGGAGAAAATTAGTAGATTTCAGAGAGCTTAATAAAAGAACTCAAGACTTCTGGGAAGTTCAACTAGGAATACCACATCCTGCAGGGCTAAAGAAGAAAAAGTCAGTAACAGTACTAGATGTGGGTGATGCATATTTTTCAGTTCCCTTATATGAAGATTTTAGAAAATATACCGCATTCACCATACCTAGTATAAACAATGAGACACCAGGAATTAGATATCAGTACAATGTGCTTCCACAGGGATGGAAAGGATCGCCGGCAATATTCCAAAGTAGCATGACAAAAATCTTAGAACCTTTTAGAAAACGAAATCCAGAAGTGGTTATCTATCAATACATGGATGATTTGTATGTAGGGTCTGACTTAGAGATAGGGCAGCATAGAATAAAAATAGAGGAATTAAGGGAACACCTATTGAAGTGGGGATTTACCACACCAGACAAAAAGCATCAGAAAGAACCTCCATTTCTTTGGATGG  SUBTYPE D:SUS |
| 008 | CAATGGCCATTGACAGAAGAAAAAATAAAGGCATTGATAGAAATTTGTACAGAGATGGAAAAGGAAGGAAAAATTTCAAGAATTGGGCCTGAGAATCCATACAATACTCCAGTATTTGCCATAAAAAAGAARGACAGTACWAAGTGGAGAAAATTAGTAGATTTCAGGGAACTCAATAAAAGRACCCARGACTTTTGGGAAGTTCAATTAGGRATACCACACCCAGCAGGGTTAAAARAGAAAAAATCAGTGACAGTACTAGATGTGGGGGATGCRTATTTTTCAGTWCCTTTAGATGAAAGCTTCAGGAAATATACTGCATTCACCATACCAAGTATAAACAATGAGACACCAGGAATCAGRTATCAGTACAATGTGCTTCCACAAGGATGGAAAGGATCACCAGCAATATTCCAAGCTAGCATGACAAAAATYCTGGAACCTTTTAGGAAACAAAATCCAGAAATGATTATCTATCAATACATGGATGATTTGTATGTAGGATCTGACTTAGAAATAGGGCAACATAGAGCAAAAATAGAGAAATTAAGGGAACACCTGTTRAAGTGGGGGTTTACTACACCAGACAAAAAGCATCAGAAAGAACCTCCATTCCTTTGGATGGGTTATGAACTA  SUBTYPE B:SUS |
| 009 | AATGGCCATTGACRGAAGAAAAAATAAAGGCATTGATAGAAATTTGTACAGAGATGGAAAAGGAAGGAAAAATTTCAAGAATTGGGCCTGAGAATCCATACAATACTCCAGTATTTGCCATAAAAARGAAAGACAGTACTAAGTGGAGAAAATTAGTAGATTTCAGGGAACTCAATAAAAGAACCCAAGACTTTTGGGAAGTTCAATTAGGRATACCACACCCAGCAGGGTTAAAARAGAAAAAATCAGYGACAGTACTAGATGTGGGGGATGCRTATTTTTCAGTWCCTTTAGATGAAAGCTTCAGGAAATATACTGCATTYACCATACCRAGTRTAAACAATGAGACACCAGGAATCAGRTATCAGTACAATGTGCTTCCACAAGGATGGAAAGGATCACCRGCAATATTCCAAGCTAGCATGACAAAAATYCTGGAACCTTTTAGGAAACAAAATCCAGAAATGATTATCTATCAATACATGGATGATTTGTATGTAGGATCTGACTTAGAAATAGGGCAACATAGAGCAAAAATAGAGRAATTAAGGGAACACCTGTTAAAGTGGGGGTTTACTACACCAGACAAAAAGCATCAGAAAGAACCTCCAYTCCTTTGGATTGGTTAT  SUBTYPE B:RES NRTI, NNRTI |
| 010 | AACAATGGCCATTGACAGAAGAAAAAATAAAGGCATTGATAGAAATTTGTACAGAGATGGAAAAGGAAGGAAAAATTTCAAGAATTGGGCCTGAGAATCCATACAATACTCCAGTATTTGCCATAAAAAAGAAAGACAGTACTAAGTGGAGAAAATTAGTAGATTTCAGGGAACTCAATAAAAGAACCCAAGACTTTTGGGAAGTTCAATTAGGGATACCACACCCAGCAGGGTTAAAAGAGAAAAAATCAGCGACAGTACTAGATGTGGGGGATGCGTATTTTTCAGTACCTTTAGATGAAAGCTTCAGGAAATATACTGCATTCACCATACCAAGTATAAACAATGAGACACCAGGAATCAGATATCAGTACAATGTGCTTCCACAAGGATGGAAAGGATCACCAGCAATATTCCAAGCTAGCATGAAAAAAATTCTGGAACCTTTTAGGAAACAAAATCCAGAAATGATTATCTATCAATACGTGGATGATTTGTATGTAGGATCTGACTTAGAAATAGGGCAACATAGAGCAAAAATAGAGGAATTAAGGGGACACCTGTTGAAGTGGGGGTTTACTACACCAGACAAAAAGCATCAGAAAGAACCTCCATTCCTTTGGATGGGTTATGAAC  SUBTYPE D:RES NNRTI,NRTI |
| 011 | CAGAAGARAAAATAAAAGCATTAACAGCAATTTGTGAAGARATGGAGAAGGAAGGAAAAATTACAAAAATTGGGCCTGAAAATCCATATAACACTCCAGTATTTGCCATAAAAAAGAAGGACAGTACTAAGTGGAGAAAATTAGTAGATTTCAGGGAACTCAATAAAAGAACTCAAGACTTTTGGGAAGTTCAATTAGGGATACCACACCCAGCAGGTTTAAAGAAAAACAAATCAGTGACAGTACTAGATGTGGGGGATGCATATTTTTCAGTCCCTTTAGATGAAAATTTCAGGAAGTATACAGCATTCACCATACCTAGTAYAAACAATGAGAMACCAGGGATTAGATATCAATACAATGTGCTTCCACAGGGATGGAAAGGATCACCAGCAATATTCCAAAGTAGTATGACAAAAATCTTAGAACCCTTTAGGGCACAAAATCCAGAAATGGTTATCTATCAATATGTGGATGATTTGTATGTAGGGTCTGACTTAGAAATAGGGCAACATAGAGCAAAAATAGAGGAGTTGAGAAACCATCTATTGAAGTGGGGATTTACCACACCAGACAAAAAACATCAGAAAGAACCCCCATTTCTTTGGATGGGTTATGAACT  SUBTYPE C:RES NRTI,NNRTI |
| 012 | AATGGCCATTGACAGAAGAGAAAATAAAAGCAATAACAGCAATTTGTGAAGAAATGGAGAAGGAAGGAAAAATTACAAAAATTGGGCCTGAAAATCCATATAACACTCCAGTATTTGCCATAAAAAAGAAGGACAGTACTAAGTGGAGAAAATTAGTAGATTTCAGGGAACTCAATAAAAGAACTCARGASTTTTGGGAAGTTCAATTAGGGATACCACACCCAGCAGGTTTAAAGAAAAACAAATCAGTGACAGTACTAGATGTGGGGGATGCATATTTTTCAGTCCCTTTAGATGAAAATTTCAGGAAGTATACAGCATTCACCATACCTAGTATAAACAATGAGAAACCAGGGATTAGATATCAATACAATGTGCTTCCACAGGGATGGAAAGGATCACCAGCAATATTCCAAAGTAGTATGACAAAAATCTTARAACCCTTTAGGGCACAAAATCCAGAAATGGTTATCTATCAATATGTGGATGATTTGTATGTAGGGTCTGACTTAGAAATAGGGCAACATAGAGCAAAAATAGAGGAGTTGAGAAACCATCTATTGAAGTGGGGATTTACCACACCAGACAAAAAACATCAGAAAGAACCCCCATTTCTTTGGATGGGTTATGAAC  SUBTYPE C:RES NRTI,NNRTI |
| 013 | CAATGGCCATTGACAGAAGAAAAAATAAAAGCATTAACAGAAATTTGTATGGAAATGGAGAAAGAAGGAAAAATTTCAAAAATTGGGCCTGAAAATCCATACAATACTCCAATATTTGCCATAAAGAAAAAGGACAGTACAAAATGGAGAAAATTGGTAGATTTCAGAGAACTTAACAAGAGAACGCAAGATTTCTGGGAAGTTCAATTAGGAATACCGCATCCTGCAGGGCTAAAAAARAARAAATCAGTAACAGTACTGGATGTGGGTGATGCATATTTTTCAGTTCCCTTATATGAARATTTTARGAAGTATACTGCATTCACCATACCCAGTATAAACAATGAGACACCAGGAATTAGATATCAGTACAATGTACTTCCACAGGGATGGAAAGGATCACCGGCAATATTCCAAAGTAGTATGACAAAAATCTTAGAACCCTTTAGGAAGAAAAATCCAGAAATGGTCATCTATCAATACATGGATGATTTGTATGTAGGATCTGACTTAGAAATAGGACAGCATAGAACAAAAATAGAAGAATTAAGGGAACATTTATTGAGGTGGGGATTTACCACACCAGACAAAAAACATCAGAAAGAACCTCCATTTCTTTGGATGGGTTATGAACTA  SUBTYPE D:SUS |
| 014 | GACAGAAGAAAAAATAAAAGCATTAACAGAAATTTGTACAGACATGGAAAGGGAAGGAAAAATTTCAAGAATTGGGCCTGAAAATCCATACAATACTCCAATATTTGCCATAAARAAAAAAGACAGTACTAAGTGGAGAAAATTAGTAGATTTCAGAGAACTTAATAAGAGAACTCAAGACTTTTGGGAAGTTCAGCTAGGAATACCACATCCTGGAGGGCTAAAAAAGAAGAAATCAGTAACAGTATTGGATGTGGGTGATGCATATTTTTCAGTTCCCTTATATGAAGAATTTAGAAAATATACTGCATTCACCATACCTAGTACAAACAATGAGACACCAGGGATTAGATATCAGTACAATGTGCTTCCACAAGGATGGAAAGGATCACCAGCAATATTCCAAAGTAGCATGACAAAAATCTTAGAACCTTTTAGAAAACAAAATCCAGAAATGGTTATCTGTCAATACGTGGATGATTTGTATGTAGGATCTGACTTAGAACTAGGGCAGCATAGAATAAAAATARAAAAATTAAGAGAACACCTGTTAAAGTGGGGATTTACCACACCAGACAAAAAAYATCAGAAAGAACCTCCATTTCTTTGGATGGGTTATGAACT  SUBTYPE D:RES NRTI,NNRTI |
| 015 | AACAATGGCCATTGACAGAAGAGAAAATAAAAGCATTAACAGAAATTTGTACAGAAATGGAAAAGGAAGGAAAAATTTCAAAAATTGGGCCTGAAAATCCATACAATACTCCAATATTTGCAATAAAGAAAAAAGATAGCACTAAATGGAGAAAATTAGTAGATTTCAGAGAGCTCAATAAAAGAACACAAGACTTTTGGGAAGTTCAATTAGGGATACCGCATCCAGCGGGCTTAAAAAAGAAAAAATCAGTAACAGTGCTAGATGTGGGGGACGCATATTTCTCAGTTCCTTTACATGAAAGTTTCAGGAAGTATACTGCGTTCACCATACCTAGTATAAACAATGAGACACCAGGAATCAGGTATCAGTACAATGTGCTTCCACAGGGATGGAAGGGATCACCAGCAATATTCCAGAGTAGCATGACAAAAATCTTAGAGCCCTTTAGATCAAAAAATCCAGAAATAGTTATCTATCAATACATGGATGACTTGTATGTAGGATCTGATTTAGAAATAGGGCAGCATAGAGCAAAAATAGAAGAGTTAAGARCTCATCTATTGAGCTGGGGGTTTACTACACCAGACAAAAAGCATCAGAAAGAACCTCCATTCCTTTGGATGGGTTATGAA  SUBTYPE A:SUS |
| 016 | AAACAATGGCCATTGACAGAAGAAAAAATAAAAGCATTAACAGAAATTTGTGCAGATATGGAAAAAGAAGGAAAAATTTCAAAAATTGGGCCTGAAAATCCATACAACACCCCAATATTTGCAATAAAGAAAAAAGATAGCACTAAATGGAGGAAATTAGTAGATTTCAGAGAGCTCAATAAGAGAACACAAGACTTCTGGGAAGTTCAATTAGGAATACCACATCCAGCGGGCCTAAAAAAGAAAAAATCAGTAACAGTACTAGATGTGGGGGATGCATATTTTTCAGTTCCTTTACATGAGGACTTTAGAAAGTATACTGCATTCACCATACCTAGTACAAACAATGAGACACCGGGAATCAGATACCAGTACAATGTGCTTCCACAAGGATGGAAAGGATCACCAGCAATATTCCAGAGCAGCATGACAAAGATCTTAGAGCCCTTTAGATCAAAAAATCCACAAATAATCATCTACCAATACATGGATGACTTGTATGTAGGATCTGATTTAGAAATAGGCCAGCATAGAGCAAAAATAGAAGAGCTGAGAGCTCATCTATTGAGCTGGGGGTTTACTACACCAGACAAAAAGCATCAGAAAGAACCTCCATTCCTTTGGATGGGG  SUBTYPE A:SUS |
| 017 | CAATGGCCATTGACAGAAGAAAAAATAAAAGCATTAACAGAAATTTGTAATGAGATGGAAAAGGAAGGAAAAATTTCAAAAATTGGGCCTGAGAATCCATACAATACTCCAATATTTGCTATAAAGAAAAAAGACAGCACTAAATGGAGAAAATTAGTAGATTTTAGAGAGCTCAATAAAAGAACTCAAGACTTTTGGGAAGTTCAATTGGGAATACCGCATCCTGCAGGTTTAAAAAAGAAAAAATCAGTAACAGTATTAGATGTGGGGGACGCCTATTTTTCAGTTCCCTTAGATGAAAGCTTTAGAAAGTATACTGCATTCACCATACCTAGTATAAACAATGAGACACCAGGGATCAGATATCAGTACAATGTGCTTCCACAGGGATGGAAAGGATCACCGGCAATATTCCAGGCTAGCATGACAAAAATATTAGAACCCTTTAGATCAAAAAATCCAGAAATAGTTATCTATCAATACATGGATGACTTGTATGTAGGATCTGATTTAGAAATAGGGCAGCATAGAATAAAAGTAGAGGAGTTGAGAGATCATCTATTGAAGTGGGGATTTACTACACCAGACAAAAAGCATCAAAAAGAACCTCCATTTCTTTGGATGGGTTATGAACTA  SUBTYPE A:SUS |
| 018 | AAACAATGGCCATTGACAGAAGAAAAAATAAAAGCATTAACAGAAATTTGTAATGAGATGGAAAAGGAAGGAAAAATTTCAAAAATTGGGCCTGAGAATCCATACAATACTCCAATATTTGCTATAAAGAAAAAAGACAGCACTAAATGGAGAAAATTAGTAGATTTTAGAGAGCTCAATAAAAGAACTCAAGACTTTTGGGAAGTTCAATTGGGAATACCGCATCCTGCAGGTTTAAAAAAGAAAAAATCAGTAACAGTATTAGATGTGGGGGACGCCTATTTTTCAGTTCCCTTAGATGAAAGCTTTAGAAAGTATACTGCATTCACCATACCTAGTATAAACAATGAGACACCAGGGATCAGATATCAGTACAATGTGCTTCCACAGGGATGGAAAGGATCACCGGCAATATTCCAGGCTAGCATGACAAAAATATTAGAACCCTTTAGATCAAAAAATCCAGAAATAGTTATCTATCAATACATGGATGACTTGTATGTAGGATCTGATTTAGAAATAGGGCAGCATAGAATAAAAGTAGAGGAGTTGAGAGATCATCTATTGAAGTGGGGATTTACTACACCAGACAAAAAGCATCAAAAAGAACCTCCATTTCTTTGGATGGGTTATGAACTA  SUBTYPE A:SUS |
| 019 | GCCATTGACAGAAGAAAAAATAAAAGCATTAACAGAAATTTGTCTAGAAATGGAGAAGGAAGGAAAAATTTCAAAAATTGGGCCTGAAAATCCATACAACACTCCAGTGTTTGCTATAAAGAAAAAAGATAGCACTAAATGGAGAAAATTAGTAGATTTTAGAGAACTCAATAAGAGAACTCAAGACTTCTGGGAAGTTCAGTTAGGAATACCACATCCAGCAGGATTAAAAAAGAAAAAATCAGTAACAGTATTAGATGTGGGGGACGCATATTTTTCCGTTCCCTTAGATAAAGAATTTAGAAAATATACTGCATTCACCATACCTAGTATAAACAATGAGACACCAGGAATTAGATATCAGTACAATGTGCTCCCACAGGGATGGAAAGGATCACCAGCAATATTCCAAAGTAGCATGACAAAAATCTTAGAGCCCTTTAGAAAACAAAATCCAGAAATGGTTATCTATCAATACGTGGATGATTTGCTTGTAGGATCTGACTTAGAAATAGGGCAGCATAGAGCAAAAATAGAGGAGTTAAGAGAACATCTATTGAAATGGGGATTTACCACACCAGATAAAAAACATCAAAAAGAACCTCCATTTCTTTGGATGGGTT  SUBTYPE D:RES NRTI,NNRTI |
| 020 | AACAATGGCCATTGACAGAGGAAAAAATAAAAGCATTAACAGAAATCTGTACAGAAATGGAAAAGGAAGGAAAAATTTCAAAAATTGGGCCTGAAAATCCATACAATACTCCAATATTTGCAATAAAGAAAAAAGATAGCACTAAGTGGAGAAAATTAGTAGACTTCAGAGAGCTCAATAAAAGAACACAAGACTTTTGGGAAGTTCAGTTAGGAATACCGCATCCAGCGGGCCTAAAAAAGAAAAAATCAGTAACAGTGCTAGATGTGGGGGATGCATATTTTTCAGTTCCTTTAGATAAAGAGTTCAGAAAATATACTGCATTCACCATACCTAGTACAAACAATGAGACACCAGGAATCAGGTATCAGTACAATGTGCTTCCACAGGGATGGAAAGGATCACCGGCAATATTCCAGAATAGCATGCTAAAAATTTTAGAGCCCTTTAGATCAAAGAATCCAGAAATAATTATCTATCAATACATGGATGACTTGTATGTAGGATCTGATTTAGAAATAGAGCAGCATAGATCAAAAGTAGAAGAGTTGAGAGCTCATCTATTGAGATGGGGACTAACTACACCAGACAAAAAGCATCAGAAAGAACCTCCATTCCTTTGGATGGGWTATGARCTA  SUBTYPE A:SUS |
| 021 | ACAATGGCCATTGACAGAAGAAAAAATAAAAGCACTAACAGAAATTTGTATGGAAATGGAAAAGGAAGGAAAAATTTCAAGAATTGGGCCTGAAAATCCATACAATACTCCAATATTTGCCATAAAGAAAAAAGACAGTACTAAGTGGAGAAAATTAGTAGATTTCAGAGAACTTAATAAGAGAACTCAAGACTTCTGGGAGGTTCAACTAGGAATACCACATCCTGCAGGGCTAAAAAARAAAAAATCAGTAACAGTACTGGATGTGGGTGATGCATATTTTTCAGTTCCATTGTATGAAGACTTTAGAAAATATACCGCATTCACCATACCTAGTATAAACAATGAGACACCAGGAATTAGATATCAGTACAATGTGCTTCCACAAGGATGGAAAGGATCACCAGCAATATTCCAAAGTAGTATGACAAAAATCCTAGAACCTTTTAGAAGAAAAAATCCAGAAATAGTTATCTATCAATACATGGATGATTTGTATGTAGGATCTGACTTAGAAATAGGGCAGCATAGAATAAAAATAGAGGAATTAAGGGAACACCTATTGAAGTGGGGATTTACCACACCAGACAAAAAGCATCAGAAAGAACCCCCATTTCTTTGGATGGGTTATGAACTA  SUBTYPE D:SUS |
| 022 | ACAATGGCCATTGACAGAAGAAAAAATAAAAGCACTAACAGAAATTTGTATGGAAATGGAAAAGGAAGGAAAAATTTCAAGAATTGGGCCTGAAAATCCATACAATACTCCAATATTTGCCATAAAGAAAAAAGACAGTACTAAGTGGAGAAAATTAGTAGATTTCAGAGAACTTAATAAGAGAACTCAAGACTTCTGGGAGGTTCAACTAGGAATACCACATCCTGCAGGGCTAAAAAAGAAAAAATCAGTAACAGTACTGGATGTGGGTGATGCATATTTTTCAGTTCCCTTGTATGAAGACTTTAGAAAATATACCGCATTCACCATACCTAGTATAAACAATGAGACACCAGGAATTAGATATCAGTACAATGTGCTTCCACAAGGATGGAAAGGATCACCAGCAATATTCCAAAGTAGTATGACAAAAATCCTAGAACCTTTTAGAAGAAAAAATCCAGAAATAGTTATCTATCAATACATGGATGATTTGTATGTAGGATCTGACTTAGAAATAGGGCAGCATAGAATAAAAATAGAGGAATTAAGGGAACACCTATTGAAGTGGGGATTTACCACACCAGACAAAAAGCATCAGAAAGAACCCCCATTTCTTTGGATGGGTTATGAAC  SUBTYPE D:SUS |
| 023 | TTAAACAATGGCCATTGACAGAAGAAAAAATAAAAGCACTAACAGACATTTGTAATGAAATGGAAAAGGAAGGGAAAATTTCAAAGATTGGGCCTGAAAATCCATACAATACCCCAATATTTGCCATAAAGAAAAAGGACAGTACTAAGTGGAGAAAATTAGTAGATTTCAGAGAGCTTAATAAGAGAACTCAAGACTTCTGGGAAGTTCAACTAGGAATACCACATCCTGCAGGGCTAAAGAAGAAAAAATCAGTAACAGTACTGGATGTGGGTGATGCATATTTTTCAGTTCCCTTGGATGAAGACTTTAGAAAATATACTGCATTCACCATACCTAGTATAAACAATGAGACACCAGGAATTAGATATCAGTACAATGTGCTGCCACAAGGATGGAAAGGATCACCGGCAATATTTCAAAGTAGCATGACAAAAATCTTAGAGCCTTTTAGAAAACAAAATCCAGAAATGGTTATCTATCAATATATGGATGATTTGTATGTAGSATCTGACTTAGAAATAGGGCAGCATAGAATAAAAATAGAGGAATTAAGGGAACACCTATTGAAGTGGGGATTTACCACACCAGACAAAAAGCATCAGAAAGAACCCCCATTTCTTTGGATGGGTTATGAACT  SUBTYPE D:RES NRTI |
| 024 | AACAATGGCCATTGACAGAAGAAAAAATAAAAGCATTAACAGAAATTTGTACAGAAATGGAAAAGGAGGGAAAAATTTCAAAAATTGGGCCTGAAAATCCATACAATACTCCAATATTTGCGATCAAGAAAAAAGATAGCACTAAATGGAGGAAATTAGTAGACTTCAGAGAGCTCAATAAAAGAACACAAGATTTTTGGGAAGTKCAATTAGGGATACCACATCCAGCRGGCCTAAAAAAGAAAAAATCAGTAACAGTACTAGATGTGGGGGACGCATATTTTTCAGTCCCCTTAGATAAAGACTTTAGAAAATATACTGCATTCACCATACCTAGTACAAACAATGAGACACCAGGAATCAGATATCAATACAATGTGCTTCCACAGGGATGGAAAGGATCACCAGCAATATTCCAGAGTAGCATGACAAAAATCTTAGAGCCTTTTAGATTAAAGAATCCAGAAATAATTATCTATCAATACATGGATGACTTGTATGTAGGATCTGATTTAGAAATAGGGCAGCATAGAACAAAAATAGAGGAGTTAAGAGCCCATCTATTGAGCTGGGGGTTTACTACACCAGACAAAAAGCATCAGAAAGAACCTCCATTCCTTTGGATGGGWTATGA  SUBTYPE A:SUS |
| 025 | AAACAATGGCCATTGACAGAAGAAAAAATAAGAGCATTAMCAGAAATTTGTACAGAAATGGAAAAGGAAGGAAAAATTTCRAAAATTGGGCCAGAAAATCCATACAATACTCCAATATTTGCTATAAAGAAAAAAGACAGCACTAAATGGAGAAAATTAGTAGATTTCAGAGAGCTTAATAAAAGAACTCAAGATTTTTGGGAAGTTCAATTAGGAATACCGCACCCAGCGGGCCTAAAAAAGAAYAAATCAGTAACAGTACTAGATGTGGGGGACGCATATTTTTCRGTTCCCTTAGATGAAAGYTTTAGAAAGTATACTGCGTTCACCATACCTAGTACAAACAATGAGACACCAGGAATCAGATATCAGTACAATGTGCTTCCACAGGGATGGAAAGGYTCACCATCAATATTCCAGAGTAGCATGACAAAAATCTTAGARCCCTTTAGAGCAAAAAATCCAGAAATAATTATCTATCAATACATGGATGACTTGTATGTAGGATCTGATTTAGAAATAGGGCAGCATAGAACAAAARTAGAAGARTTGAGAGCTCATCTATTGAGCTGGGGATTTACTACCCCAGACAAAAARCATCAGAAAGAACCGCCATTTCTTTGGATGGGTTATGAACTA  SUBTYPE A:RES NRTI |
| 026 | AAACAATGGCCATTGACAGAAGAAAAAATAAGAGCATTAACAGAAATTTGTACAGAAATGGAAAAGGAAGGAAAAATTTCGAAAATTGGGCCAGAAAATCCATACAATACTCCAATATTTGCTATAAAGAAAAAAGACAGCACTAAATGGAGAAAATTAGTAGATTTCAGAGAGCTTAATAAAAGAACTCAAGATTTTTGGGAAGTTCAATTAGGAATACCGCACCCAGCGGGCCTAAAAAAGAATAAATCAGTAACAGTACTAGATGTGGGGGACGCATATTTTTCGGTTCCCTTAGATGAAAGTTTTAGAAAGTATACTGCGTTCACCATACCTAGTACAAACAATGAGACACCAGGAATCAGATATCAGTACAATGTGCTTCCACAGGGATGGAAAGGCTCACCATCAATATTCCAGAGTAGCATGACAAAAATCTTAGAACCCTTTAGAGCAAAAAATCCAGAAATAATTATCTATCAATACGTGGATGACTTGTATGTAGGATCTGATTTAGAAATAGGGCAGCATAGAACAAAAATAGAAGAATTGAGAGCTCATCTATTGAGCTGGGGATTTACTACCCCAGACAAAAAGCATCAGAAAGAACCGCCATTTCTTTGGATGGGTTATGAACTAA  SUBTYPE A:RES NRTI,NNRTI |
| 027 | AAACAATGGCCATTGACAGAAGAAAAAATAAAAGCATTAACAGAAATTTGTACAGAGATGGAAAAGGAAGGGAAAATTTCAAAAATTGGACCTGAAAATCCATACAATACTCCAATATTTGCTATAAAGAAAAAAGATAGCACTAAATGGAGAAAATTAGTAGATTTCAGAGAGCTCAATAAGAGAACTCAGGACTTCTGGGAAGTTCAATTAGGAATACCACACCCAGCAGGTTTAAAAAAGAAGAAATCGGTAACAGTACTAGATGTGGGGGATGCATATTTTTCAGTTCCTTTAGATGAAAGCTTTAGAAAGTATACTGCATTCACCATACCTAGTACAAACAATGAGACACCAGGAGTCAGGTATCAATATAATGTGCTTCCACAGGGATGGAAAGGATCACCAGCAATATTCCAGAGTAGCATGACAAAAATCTTAGAGCCCTATAGATCAAAAAATCCAGAAATAATTATTTATCAATACATGGATGATTTGTATGTAGCATCTGATTTAGAAATAGGACAACATAGAGCAAAAATAGAGGAGCTGAGAGCTCATCTATTAAGTTGGGGGTTTACTACACCAGACAAAAAGCATCAGAAAGAACCCCCATTTCTTTGGATGG  CRF01_AE:RES NNRTI |
| 028 | TGGCCATTGACAGAAGAAAAAATAAAAGCATTAACAGAAATTTGTACAGAGATGGAAAAGGAAGGGAAAATTTCAAAAATTGGACCTGAAAATCCATACAATACTCCAATATTTGCTATAAAGAAAAAAGATAGCACTAAATGGAGAAAATTAGTAGATTTCAGAGAGCTCAATAAGAGAACTCAGGACTTCTGGGAAGTTCAATTAGGAATACCACACCCAGCAGGTTTAAAAAAGAAGAAATCGGTAACAGTACTAGATGTGGGGGATGCATATTTTTCAGTTCCTTTAGATGAAAGCTTTAGAAAGTATACTGCATTCACCATACCTAGTACAAACAATCAGACACCAGGAGTCAGGTATCAATATAATGTGCTTCCACAGGGATGGAAAGGATCACCAGCAATATTCCAGAGTAGCATGACAAAAATCTTAGAGCCCTATAGATCAAAAAATCCAGAAATAATTATTTATCAATACGTGGATGATTTGTATGTAGCATCTGATTTAGAAATAGGACAACATAGAGCAAAAATAGAGGAGCTGAGAGCTCATCTATTAAGTTGGGGGTTTACTACACCAGACAAAAAGCATCARAAAGAACCCCCATTTCTTTGGATGGGTTATGAACT  CRF01_AE :RES NRTI,NNRTI |
| 029 | AACAATGGCCATTGACAGAAGAAAAAATAAAAGCATTAACAGAAATTTGTAAWGARATGGAAAAGGAAGGAAAAATTTCAAAAATTGGGCCTGAAAATCCATATAATACTCCAATATTTGCMATAAAGAAAAAAGACAGTACTAAGTGGAGAAAATTAGTAGATTTCAGAGAACTWAATAARAGAACTCAAGACTTYTGGGAARTTCAATTAGGAATACCRCATCCTGCAGGGCTAAAAAAYAAAAARTCAGTAACAGTACTRGATGTGGGAGATGCATATTTTTCAGTTCCCTTAYRTGAAGATTTTAGAAAGTATACTGCATTTACCATACCTAGTRYAAAYAATGAGACACCAGGGRTTAGATATCAGTACAATGTGCTTCCACAGGGATGGAAAGGRTCACCAGCAATATTYCAAAGTAGCATGACAAAAATCTTAGAACCTTTTAGAAAACAAAATCCAGARGTGGTTATCTATCAATACATGGATGATTTGTATGTAGGATCTGACTTAGAAATAGGKMARCATAGAACAAAAATAGAGGAATTAAGGGAACAYCTATTAARGTGGGGRTTTACCACACCAGACAAAAAACATCAGAAAGAGCCTCCATTTCTTTGGATGGGTTATGAACTA  SUBTYPE D:RES NRTI |
| 030 | AAACAATGGCCATTGACAGAAGAAAAAATAAAAGCATTAACAGAAATTTGTGAAGAGATGGAAAAGGAAGGAAAAATTTCAAAAATTGGGCCTGAAAATCCATACAATACTCCAGTGTTTGCTATAAAGAAAAAGGATAGCACTAAATGGAGAAAATTAGTAGATTTTAGAGAGCTCAATAAAAGAACACAGGACTTCTGGGAAGTTCAATTAGGAATACCCCATCCTGCAGGTTTAAAAAAGAAAAAATCAGTAACAGTACTAGATGTGGGGGATGCCTATTTTTCAGTTCCTTTAGATAAAGATTTTAGAAAGTATACTGCATTCACCATACCTAGTATAAACAATGAGACACCAGGAACCAGGTATCAGTACAATGTGCTTCCACAAGGATGGAAAGGATCACCAGCAATATTCCAGAGTAGCATGGCAAAAATCTTAGAGCCCTTTAGATCACAAAATCCAGGAATAATTATTTATCAATACGTGGATGACTTGTATGTAGCATCTGATTTAGAAATAGGGCAGCATAGAACAAAAGTAGAAGAATTGAGAGCTCATCTATTGAGTTGGGGATTTACTACACCAGACAAAAAACATCAGAAAGAACCTCSATTTCTTTGGATGGGTTATGAACTA  SUBTYPE A:RES NRTI,NNRTI |
| 031 | GTTAAACAATGGCCATTGACAGAAGAAAAAATAAAAGCATTAACAGAAATTTGTACAGAMATGGAAAAGGAAGGAAAAATTTCAAAAATTGGGCCTGAAAATCCATACAATACTCCAATATTTGCCATAAAGAARAAAGACAGTACTAAGTGGAGAAAGTTAGTAGATTTCAGAGAACTCAATAAAAGAACTCAAGACTTTTGGGAAGTCCAATTAGGGATACCACACCCAGCAGGGTTGAAAAAGAAAAAATCAGTGACAGTATTGGATGTGGGGGATGCATATTTTTCAGTTCCTTTAGATGAAGACTTCAGAAAATATACTGCATTTACAATACCTAGTATAAACAATGAAACACCAGGAATTAGATATCAGTATAATGTGCTTCCACAGGGATGGAAAGGATCACCAGCAATATTCCAAAGCAGCATGACAAAAATTTTAGAGCCCTTTAGGGCGCAAAACCCAGAAATAGWTATCTATCAATACATGGATGACTTGTATGTAGGATCTGACTTAGAAATAGGGCAACATAGAGCAAAAATAGAGGAGTTAAGGGAACATCTGTTGARGTGGGGGTTTACCACACCAGATAAGAAACATCAGAAAGAACCTCCATTTCTWTGGATGGGTTATGAACTA  SUBTYPE B:RES NRTI |
| 032 | CAATGGCCATTGACAGAAGAAAAAATAAAAGCACTAACAGAAATTTGTAAAGAAATGGAAAAGGAAGGAAAAATTTCAAGAATTGGGCCTGAGAATCCATACAATACTCCAATATTTGCCATAAGAAAGAAAGACAGTACTAAGTGGAGAAAATTARTGGATTTCAGGGAACTCAATAAAAGAACCCAAGACTTTTGGGAAGTTCAATTAGGGATACCACACCCAGCAGGGTTAAAAAAGAAAAAATCAGTGACAGTACTAGATGTGGGAGATGCATATTTTTCAGTTCCTTTAGATGAAGGCTTCAGAAAATATACTGCATTCACCATACCTAGTAKRAAYAATGAGACACCAGGAATTAGATATCAGTACAATGTGCTTCCGCAAGGATGGAAAGGATCACCGGCAATATTCCAAAGTAGCATGACAAAAATCTTAGAACCCTTTAGGAAACAAAATCCAGAAATGGTTATCTRTCAATACATGGATGATTTGTATGTAGGATCTGACTTAGAAATAGGGCAACATAGAATAAAAATAGGAGAGTTAAGGGAACACCTATTGAAGTGGGGATTTACTACACCAGACAAAAAGCATCAGAAAGAACCTCCATTCCTTTGGATGG  SUBTYPE D:RES NRTI,NNRTI |
| 033 | AAAAATTTCAAAAATTGGGCCTGAAAATCCATACAATACTCCAATATTTGCAATAAAGAAAAAAGATAGCACTAAATGGAGAAAATTAGTAGATTTCAGAGAGCTCAATAAAAGAACACAAGACTTTTGGGAAGTTCAATTAGGGATACCGCATCCAGCGGGCCTAAAAAAGAAAAAATCAGTAACAGTACTAGATGTGGGGGACGCCTATTTTTCAGTTCCTTTAGATGAAAACTTTAGAAAATATACTGCATTCACCATACCTAGTATAAATAATGAAACACCAGGAATAAGGTATCAGTACAATGTGCTTCCACAGGGATGGAAAGGATCACCAGCAATATTCCAGAGTAGTATGACAAAAATCTTAGAGCCCTTTAGAACAAAAAATCCAGAAATARTTATCTATCAATACATGGATGACTTGTATGTAGGATCTGATTTAGAGATAGGGCAGCATAGAGCAAAAATAGAAGAACTAAGAGCTCATCTGTTGAGCTGGGGATTTACYACACCAGACAAAAAGCATCAGAAAGAACCCCCATTCCTTTGGATGG  SUBTYPE A:SUS |
| 034 | AACAATGGCCATTGACAGAAGAAAAAATAAAAGCATTAACAGAAATTTGTACAGATATGGAAAAGGAGGGAAAAATTTCAAGAATTGGGCCTGAAAATCCATACAATACTCCAATATTTGCTATAAAGAAAAAGGACAGTACTAAATGGAGAAAATTAGTAGATTTCAGAGAACTTAATAAAAGGACTCAAGACTTTTGGGAAGTTCAATTAGGAATACCACACCCAGCAGGGTTAAAAAAGAAAAAATCAGTGACAGTACTGGATGTGGGTGATGCATATTTTTCAGTTCCTTTAGATAAAGACTTTAGAAAGTATACCGCATTCACCATACCTAGTATAAACAATGAAACACCAGGAATTAGATATCAGTACAATGTGCTCCCACAGGGATGGAAAGGATCACCGGCAATATTCCAAAGTAGCATGACAAAAATCTTAGAGCCCTTTAGAAAGAAAAATCCAGAAATAGTTATTTATCAGTACATGGATGATTTATATGTAGGATCTGACTTAGAAATAGGGCAGCATAGAACAAAAATAGAAGAATTAAGAGAGCATCTTTTAAGGTGGGGATTTACCACACCAGACAAAAAACATCAGAAGGAACCTCCATTTCTTTGGATGGGTTATGAACTA  SUBTYPE D:SUS |
| 035 | GATGGAAAAGGAAGGAAAGATTTCAAAAATAGGGCCTGAAAATCCATACAATACTCCAGTATTTGCTATAAAGAAAAAAGATAGCACTAAATGGAGAAAATTAGTAGATTTTAGAGAACTCAATAAAAGAACTCAAGACTTTTGGGAAGTCCAACTAGGGATACCACACCCAGCAGGTTTAAAGAAGAAAAAATCAGTGACAGTACTGGATGTGGGGGATGCATATTTTTCAGTCCCTTTAGATGAAAACTTCAGGAAATACACTGCATTCACAATACCTAGTATAAACAATGAAACACCAGGGATTAGATATCAATACAATGTGCTTCCACAGGGGTGGAAAGGGTCACCAGCAATATTCCAGAGTAGCATGACAAAAATCTTAGAGCCCTTTAGATCAAAAAATCCAGAAATAATTATCTATCAATACATGGATGACTTGTATGTAGGATCTGATTTAGAAATAGGGCAGCATAGAACAAAAGTAGAGGAGTTGAGGGCTCATCTATTGAGGTGGGGATTTACTACACCAGACAAAAAACATCAGAAAGAACCTCCATTTCTTTGGATGGGTTATGAACTA  SUBTYPE B:SUS |
| 036 | AAAGGAAGGAAAAATTTCAAGAATTGGGCCTGAAAATCCATACAACACTCCATTATTTGCTATAAARAAAAAAGACAGTACTAAATGGAGAAAATTAGTAGATTTCAGAGAACTTAATAAGAGAACTCAGGACTTCTGGGAAGTTCAATTAGGAATACCGCATCCAGCAGGTTTAAAAAAGAAAAAATCAGTAACAGTACTAGATGTGGGGGACGCATATTTTTCAGTTCCTTTACATGAAGACTTTARAAAGTATACTGCCTTCACCATACCTAGTACAAACAATGAGACACCAGGAGTCAGGTATCAGTACAATGTGCTCCCACAAGGATGGAAAGGATCACCAGCGATATTCCAGAGTAGCATGACAAAAATCTTAGAACCCTTTAGAACAAAAAACCCAGAAATAGTTATCTACCAATACATGGATGACTTGTATGTAGGATCTGATTTAGAAATAGGGCAGCATAGAACAAAAATAGAGGAGCTGAGAGCTCATCTATTGAGATGGGGGCTCACTACACCAGACAAAAAGCATCAGAAAGAACCTCCATTTCTTTGGATGGGTTATGAACT  SUBTYPE A:SUS |
| 037 | AACAATGGCCATTGACAGAAGAAAAAATAAAAGCATTAACAGAAATTTGTACAGAAATGGAAAAGGAAGGGAAAATTTCAAAAATTGGGCCTGAAAATCCATACAATACTCCAATATTTGCAATAAAGAAAAAAGATAGTACCAAATGGAGGAAATTAGTAGATTTCAGAGAACTTAATAAAAGAACACAAGACTTTTGGGAARTTCAATTAGGAATACCGCATCCAGCGGGCCTAAAAAAGAAAAAATCAGTAACAGTACTAGATGTGGGAGATGCATATTTTTCAGTTCCTTTAGATGAAAGTTTTAGAAAATACACTGCATTCACCATACCTAGTACAAACAATGAGACACCAGGAGTCAGATATCAGTACAATGTGCTTCCACAGGGATGGAAAGGATCTCCGGCAATATTCCAGAGTAGCATGACAAAAATCTTAGAGCCATTTAGATCAAAAAATCCAGAAATAATTATCWWTCAATACATGGATGACTTGTATGTAGGATCTGATTTAGAAATAGGGCAACATAGAACAAAAATAGAAGAGTTAAGAGCTCATCTACTGAGCTGGGGATTTACTACACCAGACAAAAAACATCAGAAAGAACCTCCATTCCTTTGGATGGGTTATGAAC  CRF01_AE :RES NNRTI |
| 038 | AACAATGGCCATTGACAGAAGAGAAAATAAAAGCATTAACAGAAATTTGTGGAGAAATGGAAAAGGAAGGAAAAATTTCAAAGATTGGGCCTGAAAATCCATACAATACTCCAATATTTGCAATAAAGAAAAAGGATAGCACTAAATGGAGGAAATTAGTAGATTTCAGAGAGCTCAATAAAAGAACACAAGACTTTTGGGAGGTTCAATTAGGAATACCACATCCAGCAGGCCTAAAAAAGAAGAAATCAGTAACAGTACTGGATGTGGGGGATGCATATTTTTCAGTGCCTTTAGATAAGGACTTCAGAAAATATACTGCATTCACCATACCTAGTACAAACAATGAGACACCAGGAATCAGATATCAGTACAATGTGCTTCCACAGGGATGGAAAGGATCACCAGCAATATTCCAGAGTAGCATGACAAAAATCTTAGAGCCCTTTAGATCAAAAAATCCAGACATAGTGATCTATCAATACATGGATGACTTGTATGTAGGATCTGATCTAGAAATAGGGCAGCATAGAACAAAAATAGAAGAGTTGAGAGCTCATCTATTGAACTGGGGATTTACTACACCAGACAAAAAGCATCAGAAAGAACCCCCATTCCTTTGGATGGGTTATGAACTA  SUBTYPE A:SUS |
| 039 | AAACAATGGCCATTGACAGAAGAAAAAATAAAAGCATTAACAGAAATTTGTACAGATATGGAAAAGGAAGGAAAGATTTCAAAAATTGGGCCTGAAAATCCATATAATACTCCAATATTTGCAATAAAGAAAAAAGATAGTACCAAATGGAGAAAATTGGTAGATTTCAGAGAGCTCAACAAAAGAACACAAGACTTTTGGGAAGTTCAATTAGGAATACCGCATCCAGCGGGCTTAAAAAAGAAAAAATCAGTAACAGTACTAGATGTGGGGGATGCATATTTTTCAGTTCCTTTAGATGAAAACTTTAGAAAATACACCGCGTTCACCATACCGAGTATAAACAATGAGACACCAGGAGTCAGATATCAGTACAATGTGCTTCCACAGGGATGGAAAGGATCACCAGCAATATTTCAGAGTAGCATGACAAAAATCTTAGAGCCCTTTAGATCAAAAAATCCAGAAATAATTATCTATCAATACATGGATGACTTGTATGTAGGATCTGATTTAGAAATAGGGCAGCATAGAACAAAAATAGAAGAGTTGAGGGCTCATCTATTGAGCTGGGGGTTCACTACACCAGACAAAAAGCATCAGAAAGAACCTCCATTTCTTTGGATGG  SUBTYPE A:SUS |
| 040 | CAATGGCCATTGACAGAAGAGAAAATAAAAGCTTTAATAGAAATTTGTACAGAAATGGAAAAGGAAGGAAAAATTTCAAAAATTGGGCCTGAAAATCCATACAATACTCCAATATTTGCCATAAAGAAAAAGGACAGTACTAAGTGGAGAAAACTAGTAGATTTCAGAGAGCTCAATAAAAGAACTCAAGATTTCTGGGAAGTCCAATTAGGGATACCTCACCCCGCGGGTCTAAAGAAGAAAAAATCAGTAACAGTACTAGATGTGGGGGATGCATATTTCTCAGTTCCCTTAGATGAAAACTTTAGAAAGTATACAGCATTCACTATACCTAGTGTAAATAATGAGACACCAGGGATTAGATACCAGTACAATGTGCTGCCTCAGGGATGGAAAGGATCACCAGCAATTTTTCAGAGTAGTATGACAAAAATCCTAGAGCCCTTTAGAAGAGAAAATCCAGAAATGGTAATTTGCCAATATATGGATGATTTATATGTAGGATCTGATTTAGAAATAGGGCAGCATAGAGCAAAAATAGAAGAATTAAGAAAACATCTATTGAATTGGGGATTTACCACACCAGATAAAAAATATCAGAAAGAACCCCCATTCCTTTGGATGG  SUBTYPE G:RES NRTI |
| 041 | CAATGGCCATTGACAGAAGAAAAAATAAAAGCATTAACAGAAATTTGTATAGAGATGGAAAAGGAAGGAAAAATTTCAAAAATTGGGCCTGAGAATCCATACAATACTCCAATATTTGCTATAAAGAAAAAAGACAGCACTAAATGGAGGAAACTAGTAGATTTTAGAGAGCTCAATAAAAGAACACAAGACTTCTGGGAAGTTCAATTAGGGATACCGCATCCAGCGGGACTAAAAAAGAAAAAATCAGTAACAGTACTGGATGTGGGGGACGCATATTTTTCAGTTCCTTTACATAAAGACTTTAGAAAATATACTGCATTCACCATACCTAGTACAAACAATGAGACACCAGGAATCAGGTATCAGTACAATGTGCTTCCACAGGGATGGAAAGGATCACCGGCAATTTTCCAGAGTAGCATGACAAAAATCTTAGAGCCCTTTAGATCAAATAATCCAGAAATAGTTATCTATCAATACATGGATGACTTGTATGTAGGATCTGATTTAGAACTAGGGCAGCATAGAGCAAAAATAGAAGAGTTGAGGGCGCATTTATTGAGCTGGGGATTAACTACCCCAGACAAAAAGCATCAGAAAGAGCCGCCATTTCTTTGGATGG  SUBTYPE A:SUS |
| 042 | ACAATGGCCATTGACAGAAGAAAAAATAAAAGCATTGACAGAAATTTGTACAGAGATGGAAAAGGAAGGAAAAATTTCAAGAATTGGGCCTGAAAATCCATACAATACTCCAATATTTGCAATAAAGAAAAAAGATAGTACTAAATGGAGGAAATTAGTAGACTTCAGAGAGCTCAATAAAAGAACACAAGACTTTTGGGAAGTTCAATTAGGGATACCGCATCCAGCGGGCCTAAAAAAGAAGAAATCAGTAACAGTACTAGATGTGGGGGACGCATATTTTTCAGTTCCTTTAGATGTAGACTTTAGAAAGTATACTGCGTTCACCATACCTAGTACAAACAATGAGACACCAGGAATAAGGTATCAGTACAATGTGCTTCCACAGGGATGGAAAGGATCACCGGCAATATTCCAGAGTAGCATGACAAAAATCTTAGAGCCCTTTAGATCAAAAAATCCAGAAATAATTATCTATCAATACATGGATGACTTGTATGTAGGATCTGATTTAGAAATAGGTCAGCATAGAGCAAAAGTAGAGGAGTTGAGAGCTCATCTATTGAGTTGGGGGTTTACTACACCAGATAAAAAACATCAGAAAGAACCTCCATTTCTTTGGATGGGTTATGAACTAC  SUBTYPE A:SUS |
| 043 | AACAATGGCCATTGACAGAAGAAAAAATAAAAGCATTAACAGAAATTTGTACAGAAATGGAAAAGGAAGGAAAAATTTCAAAAATTGGGCCTGAGAATCCATACAATACCCCAATATTTGCTATAAAGAAAAAAGACAGTACTAAGTGGAGAAAATTAGTGGATTTTAGAGAACTTAATAAGAGAACTCAAGATTTCTGGGAAGTTCAATTAGGAATACCACATCCTGCAGGATTAAAAAAGAAAAATTCAGTAACAGTACTGGATGTGGGTGATGCATATTTTTCAGTTCCCTTAGATGAAGACTTTAGAAAATATACCGCATTCACTATACCTAGTATAAATAACGAGACACCAGGAGTTAGATATCAGTACAACGTGCTTCCACAAGGATGGAAAGGGTCACCATCAATATTTCAAAGTAGCATGACAAAGATCTTAGAACCTTTTAGAAAACAAAATCCAGAAATAGTTATCTATCAATACATGGATGATTTGTATGTAGGATCTGACTTAGAAATAGGGCAGCATAGAACAAAAATAGAGGAATTAAGGGGACACCTATTGAAGTGGGGATTCACCACACCAGACAAAAAGCATCAGAAAGAACCTCCATTTCTTTGGATGGGTTATGAACTA  SUBTYPE D:SUS |
| 044 | AGTTAAACAATGGCCATTGACAGAAGAAAAAATAAAAGCATTAACAGAAATTTGTACGGAAATGGAAAAGGAGGGAAAAATTTCAAAAATTGGGCCTGAAAATCCATACAATACTCCAATATTTGCGATAAAGAAAAAGGATAGCACTAAATGGAGGAAATTAGTAGATTTCAGAGAGCTCAATAAAAGAACACAAGACTTTTGGGAAGTTCAATTAGGGATACCGCATCCAGCGGGCCTAAAAAAGAAAAAATCAGTAACAGTACTGGATGTGGGGGACGCATACTTTTCAGTTCCTTTACATAAGGACTTTAGAAAGTATACTGCGTTCACCATACCTAGTACCAACAATGAGACACCAGGAATCAGATATCAGTACAATGTACTTCCACAGGGATGGAAAGGATCACCAGCAATATTCCAGAGTAGCATGACAAAAATCTTAGAGCCCTTTAGATCAAAAAATCCAGAAATAATCATCTATCAATACATGGATGATTTGTATGTAGGATCTGATTTAGAAATAGGGCAACATAGAGCAAAAATAGAAGAGTTGAGAGCTCATCTCTTGAGCTGGGGATTTACTACCCCAGACAAAAAGCATCAGAAAGAACCTCCATTCCTTTGGATGGGTTATGAACTA  SUBTYPE A:SUS |
| 045 | CAGAAGAAAAAATAAAAGCATTAACAGAAATTTGTTTAGAGATGGAAAAGGAGGGAAAGATTTCAAAAATTGGGCCTGAAAATCCATACAATACTCCAATATTTGCAATAAAGAAAAAGGATAGTACTAAATGGAGAAAATTAGTAGATTTCAGAGAGCTCAATAAAAGAACACAAGACTTTTGGGAAGTTCAATTAGGGATACCGCATCCAGCGGGCCTAAAAAAGAAGAAATCAGTAACAGTACTAGATGTGGGGGATGCATATTTTTCAGTTCCTTTACATGAAGACTTTAGAAAGTATACTGCATTCACCATACCTAGCACAAACAATGAGACACCAGGAATCAGATATCAGTAYAATGTGCTTCCACAAGGATGGAAAGGATCACCAGCAATATTCCAGAGTAGCATGACAAAAATCTTAGAGCCCTTTAGGTTAAAAAATCCAGAAATAATTATCTATCAATACATGGATGACTTGTATGTAGGATCTGATTTAGAGATAGGGCAGCATAGRACAAAAATAGAAGAGTTGAGGGCTCATCTATTGAGCTGGGGATTTACTACACCAGACAAAAAGCATCAGAAAGAACCTCCATTCCTTTGGATGGGTTATGAACTA  CRF01_AE:SUS |
| 046 | CAATGGCCATTGACAGAGGAAAAAATAAAAGCATTAACAGAAATTTGTACAGAAATGGAAAAGGAAGGAAAAATTTCAAGAATTGGGCCTGAAAATCCATATAATACTCCAATATTTGCCATAAAGAAAAAAGACAGTACTAAATGGAGGAAATTAGTGGATTTCAGAGAGCTCAATAAAAGAACTCAAGATTTTTGGGAAGTTCAATTAGGAATACCGCATCCAGCGGGCTTAAAAAAGAAAAAATCAGTAACAGTACTGGATGTGGGGGACGCATATTTTTCAGTTCCCTTAGATGAAGGCTTTAGGAAGTATACGGCGTTCACCATACCTAGTACAAACAATGAGACACCAGGGATCAGGTATCAGTACAATGTGCTTCCACAGGGATGGAAAGGGTCTCCAGCAATATTCCAGAGTAGTATGACAAAAATCTTAGAGCCCTTTAGGCTAAAAAATCCAGAAGTAACTATCTATCAATACATGGATGACTTATATGTAGGGTCTGATTTAGAAATAGGGCAGCATAGAACAAAAGTAGAGGAGTTGAGAGATCATCTATTGAGCTGGGGATTAACTACACCAGACAAAAAGCATCAGAAAGAACCTCCATTTCTTTGGATGG  SUBTYPE A:RES NNRTI |
| 047 | CAATGGCCATTGACAGAAGAAAAAATAAAAGCATTAACAGAAATTTGTATAGATATGGAAAAGGAAGGAAARATTTCAAGAATTGGGCCTGAAAATCCATACAATACTCCAATATTTGCCATAAAGAAAAAAGACAGTACTAAGTGGAGAAAATTAGTAGATTTCAGAGAACTTAATAAGAGAACTCAAGACTTCTGGGAAGTTCAATTAGGAATACCACACCCTGCAGGGCTAAAAAAGAAAAAATCAGTAACAGTACTGGATGTGGGTGATGCATATTTTTCAGTTCCCCTATATGAAGATTTTAGAAAATATACTGCATTCACCATACCTAGTACAAACAATGAGACACCAGGAATCAGGTATCAGTACAATGTGCTTCCACAGGGATGGAAAGGATCACCAGCAATATTCCAGAGTAGCATGACAAAAATCTTAGAGCCCTATAGATTAAAAAATCCAGAAATAATTATCTATCAATACATGGATGACTTGTATGTGGGATCTGATTTAGAAATAGGGCAGCATAGAACAAAAATAGAAGAGTTGAGAGCTCATCTATTGAGCTGGGGATTTACCACACCAGACAAAAAGCATCAAAAAGAACCTCCATTTCTTTGGATGG  SUBTYPE D:SUS  TCCAAAAGTTAAACAATGGCCATTGACAGAAGAAAAAATAAAAGCATTAACAGAAATTTGTACAGAAATGGAAAAGGAAGGGAAAATTTCAAAAATTGGGCCTGAAAATCCATACAATACTCCAATATTTGCAATAAAGAAGAAAGATAGCACTAAATGGAGGAAACTAGTAGATTTCAGAGAGCTCAATAAAAGAACACAAGACTTTTGGGAAGTTCAATTAGGAATACCACATCCAGCAGGCCTGAAGAAGAAAAAATCAGTAACAGTACTAGATGTGGGGGATGCATATTTTTCAGTTCCTCTAGATGAAAGCTTTAGAAAGTATACTGCATTCACCATACCTAGTAGAAACAATGAGACACCAGGAATCAGGTATCAGTACAATGTGCTTCCACAAGGATGGAAAGGATCACCAGCAATATTCCAGAGTAGCATGACAAAAATCTTAGATCCCTTTAGATCAAAAAATCCAGAAATAGTTATCTATCAATACATGGATGACTTGTATGTAGGATCTGATTTAGAAATAGAGCAGCACAGAACAAAAATAGAAGAATTAAGAGCTCATCTATTGAGCTGGGGATTCACTACACCAGACAAAAAGCATCAGAAAGAACCTCCATTCCTTTGGATGGG  CRF01_AE:SUS |
| 048 |  |
| 049 | TATGGAAAAGGAAGGAAAAATTTCAAAAATTGGGCCTGAAAATCCATACAATACTCCAATATTTGCAATAAAGAAAAAAGATAGCACTAAGTGGAGGAAATTAGTAGATTTCAGAGAGCTCAATAAAAGAACACAAGACTTTTGGGAAGTCCAATTAGGAATACCGCATCCAGCGGGCCTAAAAAARAAAAAATCAGTAACAGTACTTGATGTGGGGGATGCATATTTTTCAGTTCCTTTATATGAAGACTTTAGAAAATACACAGCATTCACCATACCTAGTAYAAACAATGAGACACCAGGAATCAGATATCAGTACAATGTGCTTCCACAGGGATGGAAAGGGTCACCAGCAATATTCCAGCATAGCATGACAAAAATTTTAGAGCCCTTTAGATTAAAAAATCCAGAAATAATTATCTATCAATACATGGATGACTTGTATGTAGGATCTGATTTAGAAATAGGACAGCATAGAACAAAAATAGAAGAGTTAAGAGCTCATTTATTGAGCTGGGGATTTACTACACCAGACAAGAAGCATCAGAAAGAACCTCCATTCCTTTGGATGGGTTATGAACTA  CRF01_AE :SUS |
| 050 | AAAAATTTCAAAAATTGGGCCTGAAAATCCATACAATACTCCAATATTTGCCATAAAGAAAAAAGACAGTACTAAGTGGAGAAAACTAGTAGATTTTAGAGAGCTCAATAAAAGAACTCAAGATTTCTGGGAGATCCAATTAGGAATACCCCATCCCGCAGGTTTAAAAAAGAAYAAATCAGTCACAGTACTAGATGTGGGGGATGCATATTTTTCAGTCCCCTTAGATAAAGATTTTAGAAAATATACAGCATTCACTATACCTAGTGCAAATAATGAGACACCAGGAGTTAGATAYCAGTACAATGTGCTGCCACAGGGATGGAAAGGATCACCAGCAATCTTTCAGGCTAGCATGACAAAAATTTTAGAGCCYTTTAGAAMAGARAATCCAGACATAGTGATCTACCAATATATGGATGATTTATATGTAGGATCWGACYTAGAAATAGGGCARCATAGAGCAAAAATAGAGGAATTAAGAGAACATCTATTGAGATGGGGATTTACCACACCAGATAAAAAACATCAGAAAGAACCTCCATTMCAATGGATGGGATATGAGCTCCATCCTGACAAATGGACGGTACAGCCTATACAGCTGCCAGAAAAAGAAAGCTGGACTGTCAATGATATACAAAAGTTAGTGGGAAAACTAAATTGGGCAAGTCAGATTTATGCA  SUBTYPE G:RES NNRTI |
| 051 | CAATGGCCATTGACAGAAGAAAAAATAAAAGCATTAACAGAAATTTGTCAAGAGATGGAAAAGGAAGGAAAAATTTCAAAAATTGGGCCTGAAAATCCATACAATACTCCAGTATTTGCTATAAAGAAAAAAGATAGCACAAAATGGAGAAAATTAGTAGATTTTAGAGAACTTAATAAAAGAACTCAGGATTTTTGGGAAGTTCAATTAGGAATACCGCATCCTGCAGGTTTAAAGAAGAAAAAAGCAGTAACAGTACTGGATGTGGGGGATGCATATTTTTCAGTGCCTTTAGATGAAAACTTTAGAAAGTATACTGCATTCACCATACCTAGTACAAACAATGAGACACCAGGAATCAGGTATCAGTACAATGTGCTTCCACAGGGATGGAAGGGATCACCAGCAATATTTCAGAGTAGCATGACAAAAATCTTAGAGCCCTTTAGAGCACAAAATCCAGGAATAATTATCTATCAATACATGGATGACTTATATGTAGGATCTGATTTAGAAATAGGGCAACATAGAGCAAAAGTGGAGGAGTTGAGAGCTCATCTATTACAATGGGGATTTACTACACCAGATAAAAAACATCAGAAAGAACCTCCATTTCTTTGGATGGGTTATGAAC  SUBTYPE A:SUS |
| 052 | ATCCATACAATRBBSSAVKRKYDGCTWTAWWGAAWAAAGACAGCACTAAAYGGAGAADATTAGTASAYYTCAGAGAACTTAATAAAAGAACACCAGACTTTTGGGAAGTTCAATTAGGGATACCGCATCCAGCGGGCCTAGAAAAGAAAAAATCAGTAACAGTATTGGATGTGGGGGACGCATATTTTTCAGTGCCTTTAGATGAAAACTTTAGAAAATATACTGCATTCACCATACCTAGTACAAACAATGCGACACCAGGAGTCAGGTATCAGTACAATGTACTTCCACAGGGATGGAAAGGATCCCCAGCAATATTCCAGAGTAGCATGACAAAAATCTTAGAGCCCTTCAGATCTAAAAATCCAGACATAATTATCTATCAATACGTGGATGACTTGTATGTAGCATCTGATTTGGAAATAGGGCAGCATAGAGCAAAAATAGAAGAGTTAAGAGCTCATTTATTGAGTTGGGGATTDDCTACACCAGACAAAAAGCATCAGAAAGAAC  SUBTYPE A:RES NNRTI/NRTI |
| 053 | GTCCAAAAGTTAAACAATGGCCATTGACAGAAGAAAAAATAAAAGCATTAACAGAAATTTGTACAGATATGGAAAAGGAAGGAAAACTATCAAGGATTGGGCCTGAAAATCCATATAACACTCCAATATTTGCTATAAAGAAAAAAGACAGTACCAAGTGGAGAAAATTAGTAGATTTCAGGGAACTTAATAAGAGAACTCAAGATTTCTGGGAAGTTCAATTAGGAATACCACACCCGGCAGGGCTAAAAAARAAAAAATCAGTAACAGTACTGGATGTGGGTGATGCCTATTTTTCAGTTCCCTTATGTGAAGAGTTTARAAAATATACTGCATTTACCATACCTAGTATAAACAATGAGACACCAGGAATTAGATATCAGTACAATGTGCTTCCACAGGGATGGAAAGGATCACCAGCAATATTCCAAAGTAGCATGACAAAAATCTTAGAACCCTTTAGAGAACAAAATCCAGAAATAGTTATCTATCAATACATGGATGATTTGTATGTAGGATCTGACTTAGAAATAGGGCAGCATAGAGCAAAAATAGAGGAACTAAGAGAACATCTATTGAGGTGGGGATTTACCACACCAGATAAAAAACATCAGAAAGAACCTCCATTTCTTTGGATGGGTTATGAAM  SUBTYPE D:SUS |
| 054 | AAAAAGTTAAAACAATGGCCATTGACAGAAGAAAAAATAAAGGCATTGATAGAAATTTGTACAGAGATGGAAAAGGAAGGAAAAATTTCAAGAATTGGGCCTGAGAATCCATACAATACTCCAGTATTTGCCATAAAAAAGAAAGACAGTACTAAGTGGAGAAAATTAGTAGATTTCAGGGAACTCAATAAAAGRACCCARGACTTTTGGGAAGTTCAATTAGGRATACCACACCCAGCAGGGTTAAAARAGAAAAAATCAGTGACAGTACTAGATGTGGGGGATGCRTATTTTTCAGTWCCTTTAGATGAAAGCTTCAGGAAATATACTGCATTCACCATACCRAGTRTAAACAATGAGACACCAGGAATCAGRTATCAGTACAATGTGCTTCCACAAGGATGGAAAGGATCACCRGCAATATTCCAAGCTAGYATGACAAAAATYCTGGAACCTTTTAGGAAACAAAATCCAGAAATGATTATCTATCAATACATGGATGATTTGTATGTAGGATCTGACTTAGAAATAGGGCAACATAGAGCAAAAATAGAGRAATTAAGGGRACACCTGTTRAAGTGGGGGTTTACTACACCAGACAAAAAGCATCAGAAAGAACCTCCATTYCTTTGGATGGGGTTATGAAM  SUBTYPE D:SUS |
| 055 | TTTTCCAAAAGTTAAAACAATGGCCATTGACAGAAGAAAAAATAAAAGCATTAACAGAAATTTGTCAAGAGATGGAAAAGGAAGGAAAAATTTCAAAAATTGGGCCTGAAAATCCATACAATACTCCAGTATTTGCTATAAAGAAAAAAGATAGCACAAAATGGAGAAAATTAGTAGATTTTAGAGAACTTAATAAAAGAACTCAGGATTTTTGGGAAGTTCAATTAGGAATACCGCATCCTGCAGGTTTAAAGAAGAAAAAAGCAGTAACAGTACTGGATGTGGGGGATGCATATTTTTCAGTGCCTTTAGATGAAAACTTTAGAAAGTATACTGCATTCACCATACCTAGTACAAACAATGAGACACCAGGAATCAGGTATCAGTACAATGTGCTTCCACAGGGATGGAAGGGATCACCAGCAATATTTCAGAGTAGCATGACAAAAATCTTAGAGCCCTTTAGAGCACAAAATCCAGGAATAATTATCTATCAATACATGGATGACTTATATGTAGGATCTGATTTAGAAATAGGGCAACATAGAGCAAAAGTGGAGGAGTTGAGAGCTCATCTATTACAATGGGGATTTACTACACCAGATAAAAAACATCAGAAAGAACCTCCATTTCTTTGGATGGGGTTATGAAC  SUBTYPE A:SUS |
| 056 | ATATTATGMHATWTRMAKAARMAAAGAWAGCACTAAAKGGARRAAATTAGTAGATTTCAGAGAGCTCAACAAAAGAACACAAGACTTTTGGGAAGTTCAGTTAGGGATACCGCATCCAGGGGGCCTAAAAAAGAAGAAATCAGTAACAGTACTGGATGTGGGVGATGCATATTTTTCAGTTCCCTTAGATGAAAGCTTTAGAAAATATMCTGCATTCACCATACCTAGTACAAACAATGAGAGACCAGGAATAAGGTATCAGTACAATGTGCTTCCACAGGGATGGAAAGGATCACCAGCAATCTTCCAGAGTAGTATGACAAAAATCTTAGAGCCCTTTAGACTAWAAWWTYYWKAAAWAATTATCTGTCAATACGTGGATGACTTGTATGTAGGATCTGATTTAGAAATAGGGCAGCATAGAGCAAAAATDGCAGAATTAAGAGCTCATCTATGRAGCTGGGGATTHHAYACACCAGACAAAAAGCATCAARDDWRAACCCC  SUBTYPE A:RES NNRTI/NRTI |
| 057 | CAATGGCCATTGACAGAAGAAAAAATAAAAGCATTAACAGAAATTTGTACAGAGATGGAAAAGGAAGGAAAAATTTCAAAAATTGGGCCTGAAAATCCATACAATACTCCAGTATTTGCTATAAAGAAAAAAGATAGCACAAAATGGAGAAAATTAGTAGATTTCAGAGAGCTCAATAAAAGAACACAAGACTTTTGGGAAGTTCAATTAGGAATACCGCATCCAGCAGGCCTAAAAAAGAAAAGATCAGTAACAGTGCTAGATGTGGGAGATGCATATTTTTCAGTTCCTTTACATAAAGATTTTAGAAAGTATACTGCATTCACCATACCTAGTACAAACAATGAGACACCAGGAATCAGATATCAGTACAATGTGCTTCCACAGGGATGGAAAGGATCACCAGCAATATTCCAGTATAGCATGACAAAAATCTTAGAGCCCTTTAGATTAAAAAATCCAGAAATAGTTATCTATCAATACATGGATGACTTGTATGTGGGATCTGATTTAGAAATAGGGCAGCATAGAACAAAAATAGAAGAATTAAGAGCTCATCTATTGAGCTGGGGATTTACTACACCAGACAAAAAGCATCAGAAAGAACCTCCATTTCTTTGGATGGGTTATGAAC  SUBTYPE A:SUS |
| 058 | WWTAWADAGAMAAGATGGCACTACATGKAGGAAATTAGTAGATTTCAGAGAACTCAATAAAAGAACACAAGACTTTTGGGAAGTTCAGTTGGGAATACCACATCCAGGAGGCCTAGAAAAGAAAAAATCARTAACAGTACTAGATGTGGGGGATGCATATTTTTCAGTTCCTTTGCATGAAGACTTTAGAAAATATACTGCATTCACCATACCTAGTATAAACAATGAGACACCAGGAATCAGATATCAGTACAATGTGCTTCCACAGGGATGGAAAGGATCACCAGCAATATTCCAGAGTAGCATGACAAAAATCTTAGAGCCCTTTAGAGCAAAGAATCCAGAGATGACTATTTGTCAATACGTGGATGACTTGTATGTATCATCTGATTTAGAAATAGAGCAGCATAGAGCAAAAATDGDDGAGTTGAGAGCTCATCTATTGAACTGGGGATTTACYACCCCAGACAAAAAGCATCAGADAGAA  SUBTYPE A:RES NNRTI/NRTI |
| 059 | AAAAAATAAAAGCATTAACAGAAATTTGTATGGAAATGGAGAAAGAAGGAAAAATTTCAAAAATTGGGCCTGAAAATCCATACAATACTCCAATATTTGCCATAAAGAAAAAGGACAGTACAAAATGGAGAAAATTGGTAGATTTCAGAGAACTTAACAAGAGAACGCAAGATTTCTGGGAAGTTCAATTAGGAATACCGCATCCTGCAGGGCTAAAAAARAARAAATCAGTAACAGTACTGGATGTGGGTGATGCATATTTTTCAGTTCCCTTATATGAAGATTTTAGGAAGTATACTGCATTCMCCATACCCAGTATAAACAATGAGACMCCAGGAATTAGATATCAGTACAATGTACTTCCACAGGGATGGAAAGGATCACCGGCAATATTCCAAAGTAGTATGACAAAAATCTTARAACCCTTTAGGAAGAAAAATCCAGAAATGGTCATCTATCAATACATGGATGATTTGTATGTAGGATCTGACTTAGAAATAGGACAGCATAGAACAAAAATAGAAGAATTAAGGGAACATTTATTGAGGTGGGGATTTACCACACCAGACAAAAAACATCAGAAAGAACCTCCATTTCTTTGGATGGGTTA  SUBTYPE B:SUS |
| 060 | AAACAATGGCCATTGACAGAAGAAAAAATAAAAGCATTAACAGCAATTTGTGACGAAATGGAAAAAGAAGGAAAGATTACAAAAATTGGGCCTGAAAATCCATATAACACTCCAGTATTTGCTATAAAAAAGAAGGACAGTACAAAATGGAGAAAATTAGTAGATTTCAGGGAACTCAATAAAAGAACTCAAGACTTTTGGGAAGTTCAATTAGGAATACCGCACCCGGCAGGGTTAAAAAAGAAAAAATCAGTGACAGTACTGGATGKGGGGGATGCATATTTTTCAGTACCTTTAGATAAAGACTTCAGGAAATATACTGCATTCACCATACCTAGTATAAACAATGAAACACCGGGAATTAGATATCAATATAATGTGCTTCCACAAGGATGGAAAGGATCACCAGCAATATTCCAGAGTAGCATGACAAGAATCTTAGAGCCTTTTAGAGCAAAAAACCCAGAAATGGTTATCTATCAATATATGGATGACTTATATGTAGGATCTAATTTAGAAATGATGCAACATAGAGCAAAAATAGAGGAGTTAAGAGAACACCTATTGAGATGGGGATTTACCACACCAGACAAGAAACATCAGAAAGAACCCCCATTTCTTTGGATGGGTTATGAA  SUBTYPE C:SUS |
| 061 | CAATGGCCATTGACAGAAGAAAAAATAAAAGCACTAACAGAAATTTGTATAGACATGGAAAAGGAAGGAAAAATTTCAAGAATTGGGCCTGAAAATCCATACAATACTCCAATATTTGCTATAAAGAAAAAAGACAGTACCAAGTGGCGAAAATTAGTAGATTTCAGAGAACTTAATAAGAGAACTCAAGATTTCKGGGAAGTTCAACTAGGAATACCACATCCTGCAGGGCTAAAAAAGAAAAAATCAGTTACAATACTGGATGTGGGTGATGCATATTTTTCAGTTCCCTTGGATAAAGAATTTAGAAAATACACTGCATTCACCATACCTAGTATAAACAATGAGACACCAGGAATTAGATATCAGTATAATGTGCTTCCACAAGGGTGGAAAGGATCACCAGCAATATTCCAAAGTAGCATGACAAAAATCTTAGAGCCCTTTAGGAAACAAAATCCAGAAATAGTTATCTRTCAATACATGGATGACTTGTATGTAGGGTCTGACTTAGAAATAGGGCAGCATCGAGCAAAAATAGAACAGTTGAGAGCTCATCTATTGAGATGGGGATTTAMTACACCAGACAAGAAGCATCAGAAAGAACCTCCATTTCTTTGGATGGGTTATGAACTMAA  SUBTYPE D |
| 062 | TGGCCATTGACAGAAGAAAAAATAAAAGCATTAACAGAAATTTGTACAGAGATGGAAAAGGAAGGGAAAATTTCAAAAATTGGACCTGAAAATCCATACAATACTCCAATATTTGCTATAAAGAAAAAAGATAGCACTAAATGGAGAAAATTAGTAGATTTCAGAGAGCTCAATAAGAGAACTCAGGACTTCTGGGAAGTTCAATTAGGAATACCACACCCAGCAGGTTTAAAAAAGAAGAAATCGGTAACAGTACTAGATGTGGGGGATGCATATTTTTCAGTTCCTTTAGATGAAAGCTTTAGAAAGTATACTGCATTCACCATACCTAGTACAAACAATGAGACACCAGGAGTCAGGTATCAATATAATGTGCTTCCACAGGGATGGAAAGGATCACCAGCAATATTCCAGAGTAGCATGACAAAAATCTTAGAGCCCTATAGATCAAAAAATCCAGAAATAATTATTTATCAATACATGGATGATTTGTATGTAGCATCTGATTTAGAAATAGGACAACATAGAGCAAAAATAGAGGAGCTGAGAGCTCATCTATTAAGTTGGGGGTTTACTACACCAGACAAAAAGCATCAGAAAGAACCCCCATTTCTTTGGATGGGTTATGAAC  Subtype A  **G190A** |
| 063 | AACAATGGCCATTGACAGAAGAAAAAATAAAGGCATTGATAGAAATTTGTACAGAGATGGAAAAGGAAGGAAAAATTTCAAGAATTGGGCCTGAGAATCCATACAATACTCCAGTATTTGCCATAAAAAAGAAAGACAGTACTAAGTGGAGAAAATTAGTAGATTTCAGGGAACTCAATAAAAGAACCCAAGACTTTTGGGAAGTTCAATTAGGGATACCACACCCAGCAGGGTTAAAAGAGAAAAAATCAGCGACAGTACTAGATGTGGGGGATGCGTATTTTTCAGTACCTTTAGATGAAAGCTTCAGGAAATATACTGCATTCACCATACCAAGTATAAACAATGAGACACCAGGAATCAGATATCAGTACAATGTGCTTCCACAAGGATGGAAAGGATCACCAGCAATATTCCAAGCTAGCATGAAAAAAATTCTGGAACCTTTTAGGAAACAAAATCCAGAAATGATTATCTATCAATACGTGGATGATTTGTATGTAGGATCTGACTTAGAAATAGGGCAACATAGAGCAAAAATAGAGGAATTAAGGGGACACCTGTTGAAGTGGGGGTTTACTACACCAGACAAAAAGCATCAGAAAGAACCTCCATTCCTTTGGATGGGTTATGAAC  SUBTYPE D:RES NNRTI/NRTI |
| 064 | TCCAAAAGTTAAACAATGGCCATTGACAGAAGAAAAAATAAAAGCATTAACAGAAATTTGTACAGACATGGAAAAGGAAGGAAAAATTTCAAAAATTGGGCCTGAAAACCCATACAATACTCCAGTATTTGCTATAAAGAAAAAAGATAGCACTAAATGGAGAAAACTAGTAGATTTTAGAGAGCTCAATAAAAGAACTCAAGACTTCTGGGAGGTTCAATTAGGAATACCGCATCCCGCAGGTTTAAAAAAGAAGAAATCAGTAACAGTACTAGATGTGGGGGACGCATATTTCTCAGTTCCTTTAGATGAAAATTTTAGAAAGTACACTGCATTCACCATACCTAGTATAAACAATGAGACACCAGGAATCAGGTATCAGTACAATGTGCTTCCACAAGGATGGAAGGGATCACCAGCAATATTTCACAGTAGCATGACAAAAATCTTAGAGCCCTTTAGATCAAAAAATACAGAAATAATTATCTATCAATACATGGATGACCTGTATGTAGCATCTGATTTAGAAATAGGGCAGCATAGAGCAAAAGTAGAGGAATTAAGAGCTCATCTATTGAGCTGGGGGCTTACTACACCAGACAAAAAGCATCAGAAAGAACCTCCATTTCTTTGGATGGGTTA  Subtype A  **G190A** |
| 065 | GTTAAACAATGGCCATTGACAGAAGAAAAAATAAAAGCATTAACAGAAATTTGTCTAGAAATGGAGAAGGAAGGAAAAATTTCAAAAATTGGGCCTGAAAATCCATACAACACTCCAGTGTTTGCTATAAAGAAAAAAGATAGCACTAAATGGAGAAAATTAGTAGATTTTAGAGAACTCAATAAGAGAACTCAAGACTTCTGGGAAGTTCAGTTAGGAATACCACATCCAGCAGGATTAAAAAAGAAAAAATCAGTAACAGTATTAGATGTGGGGGACGCATATTTTTCCGTTCCCTTAGATGAAGAATTTAGAAAATATACTGCATTCACCATACCTAGTATAAACAATGAGACACCAGGAATTAGATATCAGTACAATGTGCTCCCACAGGGATGGAAAGGATCACCAGCAATATTCCAAAGTAGCATGACAAAAATCTTAGAGCCCTTTAGAAAACAAAATCCAGAAATGGTTATCTATCAATACGTGGATGATTTGCTTGTAGGATCTGACTTAGAAATAGGGCAGCATAGAGCAAAAATAGAGGAGTTAAGAGAACATCTATTGAAATGGGGATTTACCACACCAGATAAAAAACATCAAAAAGAACCTCCATTTCTTTGGATGGGWTATGAACTA  SUBTYPE A:RES NNRTI/NRTI |
| 066 | ATGGCCATTGACAGAAGAAAAAATAARAGCATTAACAGAAATTTGTGCAGACATGGAAAAGGAAGGAAAAATTTCAAAAATTGGGCCTGAAAACCCATACAATACTCCAGTATTTGCTATAAAGAAAAAAGATAGCACYAAATGGAGAAAACTAGTAGATTTTAGAGAGCTCAATAAAAGAACTCAAGACTTCTGGGAAGTTCAATTAGGAATACCGCATCCCGCAGGKTTAAAAAAGAAGAAATCAGTAACAGTACTAGATGTGGGGGACGCATATTTCTCAGTTCCTTTAGATGAAAATTTTAGAAAGTACACTGCATTCACCATACCTAGTATAAACAATGAGACACCAGGAATCAGGTATCAGTACAATGTGCTTCCACAGGGATGGAAGGGATCACCAGCAATATTTCAGAGTAGCATGACAAAAATCTTAGAGCCCTTTAGATCAAAAAATACAGAAATAATTATCTATCAATACATGGATGACCTGTATGTAGCATCTGATTTAGAAATAGGGCAGCATAGAGAAAAAGTAGAGGAATTAAGAGCTCATCTATTGAGTTGGGGGCTTACTACACCAGACAAAAAGCATCAGAAAGAACCTCYATTTCTTTGGATGGGTTA  Subtype A  **G190A** |
| 067 | AAACAATGGCCATTGACAGAAGAAAAAAWAAAAGCATTAACMGAGATTTGTACAGATATGGAAAAGGAAGGAAAAATTTCAAAAATTGGGCCTGAAAATCCATACAATACTCCCATATTTGCAATAAAGAAAAAAGATAGCACTAAATGGAGAAAATTAGTAGATTTCAGAGAACTTAATAAAMGAACACAAGACTTTTGGGAAGTTCAATTAGGAATACCGCATCCAGCGGGCCTAAAAAGAAAAAGATCAGTAACAGTACTAGATGTGGGGGATGCATATTTTTCAGTACCCTTATATGAAGATTTTAGAAAGTATACTGCATTCACCATACCTAGTACAAACAATGAGACACCAGGAATCAGATATCAGTACAATGTGTTGCCGCAGGGATGGAAGGGATCACCAGCAATATTCCAGAGTAGCATGACAAAAATCTTAGAGCCCTTTAGATCAAAAAATCCAGAAATAATTATCTATCAATACATGGATGACTTATATGTAGGATCTGATTTAGAAATAGGGCAGCATAGAGCAAAGATAGAAGAGCTAAGAGCTCATCTATTGAGCTGGGGATTTACTACACCAGACAAAAAGCATCAAAAAGAACCTCCATTTCTTTGGATGGGTTATGAACTAA  SUBTYPE A:SUS |
| 068 | AAACAATGGCCATTGACAGAAGAAAAAATAAAAGCATTAACAGAAATTTGTCTAGAAATGGAGAAGGAAGGAAAAATTTCAAAAATTGGGCCTGAAAATCCATACAACACTCCAGTGTTTGCTATAAAGAAAAAAGATAGCACTAAATGGAGAAAATTAGTAGATTTTAGAGAACTCAATAAGAGAACTCAAGACTTCTGGGAAGTTCAGTTAGGAATACCACATCCAGCAGGATTAAAAAARAAAAAATCAGTAACAGTATTAGATGTGGGGGACGCATATTTTTCCGTTCCCTTAGATGAAGAATTTAGAAAATATACTGCATTCACCATACCTAGTATAAACAATGAGACACCAGGAATTAGATATCAGTACAATGTGCTCCCACAGGGATGGAAAGGATCACCAGCAATATTCCAAAGTAGCATGACAAAAATCTTAGAGCCCTTTAGAAAACAAAATCCAGAAATGGTTATCTATCAATACATGGATGATTTGTATGTAGGATCTGACTTAGAAATAGGGCAGCATAGAGCAAAAATAGAGGAGTTAAGAGAACATCTATTGAAATGGGGATTTACCACACCAGATAAAAAACATCARAAAGAACCTCCATTTCTTTGGATGGGTTATGAAC  SUBTYPE A:SUS |
| 069 | CAATGGCCATTGACAGAAGAAAAAATAAAAGCATTAACAGAAATTTGTGCAGACATGGAAAAGGAAGGAAAAATTTCAAAAATTGGGCCTGAAAACCCATACAATACTCCAGTATTTGCTATAAAGAAAAAAGATAGCACTAAATGGAGAAAACTAGTAGATTTTAGAGAGCTCAATAAAAGAACTCAAGACTTCTGGGAGGTTCAATTAGGAATACCGCATCCCGCAGGTTTAAAAAARAARAAATCAGTAACAGTACTAGATGTGGGGGACGCATATTTCTCAGTTCCTTTAGATGAAAATTTTARAAAGTACACTGCATTCACCATACCTAGTATAAACAATGAGACACCAGGAATCAGGTATCAGTACAATGTGCTTCCACAGGGATGGAAGGGATCACCAGCAATATTTCAGAGTAGCATGACAAAAATCTTAGAGCCCTTTAGATCAAAAAATACAGAAATAATTATCTATCAATACATGGATGACCTGTATGTAGCATCTGATTTAGAAATAGGGCAGCATAGAGCAAAAGTAGAGGAATTAAGAGCTCATCTATTGAGTTGGGGGCTTACTACACCAGACAAAAAGCATCAGAAAGAACCTCCATTTCTTTGGATGGGTTATGAACTACATCCTGATAAA  Subtype A  G190A |
| 070 | CAATGGCCATTGACAGAAGAAAAAATAAAGGCATTGAYAGAAATTTGTACAGAGATGGAAAAGGAAGGAAAAATTTCAAGRRTTGGRCCTGAGAATYCATACAATACTCCARTATTTGCCATAAAAAAGAARGACAGTACWAAGTGGAGAAAATTAGTAGATTTCAGGGAACTCAATAAAAGRACCCARGACTTTTGGGAAGTTCAATTAGGRATACCACACCCAGCAGGGTTAAAARAGAAAAAATCAGTGACAGTACTAGATGTGGGGGATGCRTATTTTTCAGTWCCTTTAGATGAAAGCTTCAGGAAATATACTGCATTCACCATACCRAGTRTAAACAATGAGACACCAGGAATCAGRTATCAGTACAATGTGCTTCCACAAGGATGGAAAGGATCACCRGCAATATTCCAAGCTAGYATGACAAAAATYCTGGAACCTTTTAGGAAACAAAATCCAGAAATGATTATCTATCAATACATGGATGATTTGTATGTAGGATCTGACTTAGAAATAGGGCAACATAGAGCAAAAATAGAGRAATTAAGGGRACACCTGTTRAAGTGGGGGTTTACTACACCAGACAAAAAGCATCAGAAAGAACCTCCATTYCTTTGGATGGGTTATGAACTA  CRF10_CD  Susceptible |
| 071 | CAATGGCCATTGACAGAAGAAAAAATAAAAGCATTAACAGAAATTTGTCAAGAGATGGAAAAGGAAGGAAAAATTTCAAAAATTGGGCCTGAAAATCCATACAATACTCCAGTATTTGCTATAAAGAAAAAAGATAGCACAAAATGGAGAAAATTAGTAGATTTTAGAGAACTTAATAAAAGAACTCAGGATTTTTGGGAAGTTCAATTAGGAATACCGCATCCTGCAGGTTTAAAGAARAAAAAAGCAGTAACAGTACTGGATGKGGGGGATGCATATTTTTCAGTGCCTTTAGATGAAAACTTTARAAAGTATACTGCATTCMCCATACCTAGTACAAACAATGAGACACCAGGAATCAGGTATCAGTACAATGKGCTTCCACAGGGATGGAAGGGATCMCCAGCAATWTTTCARAGTAGCATGACAAAAATYTTAGAGCCCTTTAGAGCACAAAATCCAGGAATAATTATCTATCAATACATGGATGACTTATATGTAGGATCTGATTTARAAATAGGGCAACATARAGCAAAAGTGGAGGAGTTGAGAGCTCATCTATTACAATGGGGATTTACTACACCARATAAAAAACATCAGAAAGAACCTCCATTTCTTTGGATGGGTTATGAACT  SUBTYPE A:SUS |
| 072 | CATTAHCAGATATTTGTACAGAAATGGAAAAGGAAGGAAAAATTTCAAGAATTGGGCCTGAAAATCCATATAATACACCAATATTTGCCATAAAGAAAAAAGATAGTACTAAGTGGAGAAAATTAGTAGATTTCAGAGAACTTAATAAGAGAACTCAAGACTTCTGGGAAGTTCAACTAGGRATACCACATCCTGCAGGGCTAAAAAAGAAAAAATCAGTAACAGTACTGGATGTGGGTGATGCATATTTTTCAGTGCCCTTAYRTGAAGACTTTAGAAAATATACTGCATTCACCATACCTAGTAKAAACAATGAAACACCAGGAATTAGATATCAGTACAATGTGCTTCCACAAGGCTGGAAAGGATCACCGGCAATATTCCAAAGTAGCATGACAAAGATCTTAGAACCTTTTAGAAAACAAAACCCGGAAATGGTTATTTATCAATACATGGATGATTTGTATGTAGGATCTGATTTAGAAATAGGGCAGCATAGAACRAAAATAGAAGAATTAAGGGAGCACCTATTGAAGTGGGGCTTTACCACACCAGACAAAAAGCATCAGAAAGAACCTCCATTTCTTTGGATGGGTTATG  SUBTYPE D:SUS |
| 073 | CAATGGCCATTGACAGAAGAAAAAATAAAAGCATTAACAGAAATTTGTACAGAAATGGAAAAGGAAGGAAAAATTTCAAAAATTGGGCCTGAAAATCCATACAATACTCCCATATTTGCAATAAAGAAAAAAGATAGCACTAAGTGGAGAAAATTAGTAGATTTCAGAGAGCTCAATAAAAGAACACAAGACTTTTGGGAGGTTCAATTAGGAATACCGCATCCAGCGGGCCTAAAAAARAAAAAATCAGTGACAGTACTAGATGTGGGGGATGCATACTTTTCAGTTCCCTTAGATGAAAATTTTARAAAGTATACTGCATTCACCATACCTAGCACAAACAATGAGACACCAGGAATCAGGTATCAGTACAATGTGCTTCCACAGGGATGGAAAGGATCACCGGCAATATTCCAGAGTAGCATGACAAAAATTTTAGAGCCCTTTAGATTAAAAAATCCAGAAATAATCATCTATCAATACATGGATGACTTGTATGTAGGATCTGATTTAGAACTAGGGCAGCATAGAGCAAAAATAGAGGAGTTGAGAGCTCATCTATTGAGCTGGGGATTTACTACACCAGACAAAAAGCATCAGAAAGAACCTCCATTTCTTTGGATGGGTTATRAAC  SUBTYPE A:SUS |
| 074 | AAAGCATTAHCAGAAATTTGTACAGAGATGGAAAAGGAAGGAAAAATTTCAAAAATTGGGCCTGAAAATCCATACAATACTCCAATATTTGCAATAAAGAAAAAAGATAGCACTAAATGGAGAAAATTAGTAGATTTCAGAGAGCTCAATAAAAGAACACAAGACTTTTGGGAAGTTCAATTAGGAATACCGCATCCAGCGGGTTTAAAAAAGAAAAAATCAGTAACAGTATTAGATGTGGGGGATGCATATTTCTCAGTTCCTTTAGATGAAAGCTTTAGAAAGTATACTGCATTCACCATACCTAGTACAAACAATGAGACACCAGGGATCAGATATCAGTACAATGTGCTTCCACAGGGATGGAAAGGATCACCAGCAATATTCCAGAGTAGCATGACAAAAATCTTAGAGCCCTTTAGAGCAAAAAATCCAGAAATAATTATTTATCAATACATGGATGACTTATATGTAGGATCTGATTTAGAAATAGGGCAGCATAGAGCAAAAATAGAGGAGTTAAGAGCTCATCTATTGAGRTGGGGATTTACTACACCAGACAAAAAGCATCAGAAAGAACCCCCATTCCTTTGGATGGGTTATGAA  SUBTYPE A:SUS |
| 075 | AAACAATGGCCATTGACAGAAGAAAAAATAAAAGCATTAACAGAAATTTGCAAAGAGATGGAAAAGGAAGGAAAAATTTCAAAAATTGGGCCTGACAATCCATACAATACTCCAGTATTTGCTATAAAGAAAAAAGATAGCACTAAATGGAGAAAATTAGTAGATTTTAGAGAGCTCAATAAAAGAACTCAGGACTTCTGGGAAGTTCAATTGGGGATACCACATCCCGCAGGCTTAAAAAAGAAAAAATCAGTAACAGTATTAGATGTAGGGGACGCATATTTTTCAGTCCCTTTAGATGAAAACTTTAGAAAGTATACTGCATTCACCATACCTAGTACAAACAATGAGACACCAGGAATCAGGTATCAGTACAATGTGCTTCCACAGGGATGGAAAGGATCACCAGCAATATTCCAGAGTAGCATGATAAAAATCTTAGAGCCCTTTAGATCAAAAAATCCAGAAATAATTATATATCAATACATGGATGACTTATATGTAGGATCTGATTTAGAAATAGGGCAGCATAGAACAAAAGTAGAGGAGTTGAGAGCTCATCTATTGAATTGGGGATTTACTACACCAGACAAAAAGCATCAGAAAGAACCCCCATTTCTTTGGATGGGATATGA  SUBTYPE A:SUS |
| 076 | TAACAGAAATTTGTACAGATATGGAAAAGGAAGGAAAAATTTCAAGAATTGGGCCTGAAAATCCATACAATACTCCARTATTTGCCATAAAGAAAAAAGACAGTACTAAGTGGAGAAAATTASTAGATTTTAGAGAACTTAATAAAAGAACTCAAGACTTCTGGGAAGTTCAACTAGGAATACCACATCCTGCAGGGCTAAAAAARAAAAAATCAGTAACAGTACTGGACGTGGGTGATGCATWTTTTTCAGTTCCCTTARATGAAAACTTTARAAAATATACCGCATTCACCATACCCAGTATAAATAATGAGACACCAGGAATTAGATACCAGTACAATGTGCTTCCACAAGGATGGAAAGGATCACCAGCAATATTCCAAAGCAGCATGACAAAAATCCTAGAACCTTTTAGGAAACAAAATCCAGAAATRGTTATCTATCAATACATGGATGATTTGTATGTAGGATCTGACTTARAAATAGGGCAGCATAGAACAAAGATAGAGGAACTAAGGGAACACTTATTGAAGTGGGGGTTTACCACACCARACAAAAAGCATCAGAAAGAACCTCCATTTCTTTGGATGGGTTATGAACTA  Subtype D  **Y115YF** |
| 077 | AAACAATGGCCATTGACAGAAGAAAAAATAAAGGCATTAACAGAAATTTGTACAGAAATGGAAAAGGAAGGAAAAATCTCAAGAATTGGGCCTGAAAATCCATACAATACTCCAGTATTTGCTATAAAGAAAAAAGACAGCACCAAATGGAGGAAATTAGTAGATTTCAGAGAACTCAATAAAAGAACTCAGGATTTCTGGGAAGTTCAATTAGGAATACCGCATCCAGCAGGTTTAAAAAARAAAAAATCAGTAACAGTACTAGATGTGGGGGACGCATATTTTTCAGTGCCTTTAGATGAAAACTTTAGAAAGTATACTGCATTCACCATACCTAGTACAAACAATGAGACACCAGGAATCAGATATCAGTATAATGTGCTTCCACAGGGATGGAAAGGATCACCAGCAATATTCCAAAGCAGCATGACAAAAATCTTAGAGCCCTTTAGATCAAAAAATCCAGAAATAATTATCTATCAATACATGGATGACTTGTATGTAGGATCTGATTTAGAAATAGGGCAGCATAGAGAGAAAATAGAGGAGTTAAGGGCTCATCTATTGAGCTGGGGGTTTACTACACCAGACAAAAAGCATCAGAAGGAACCTCCATTTCTTTGGATGGGTTATGAACTA  CRF01_AE:SUS |
| 078 | CATTAACAGAAATTTGTACAGAAATGGAAAAGGAAGGAAAAATTTCAAAAATTGGGCCTGAAAATCCATACAATACTCCAATATTCGCGATAAAGAAAAAAGACAGCACTAAATGGAGAAAATTAGTAGATTTCAGAGAGCTCAATAAAAGAACTCAAGACTTTTGGGAAGTTCAATTAGGAATACCGCATCCAGCGGGCTTAAAAAAGAAAAAATCAGTAACAGTACTAGATGTGGGGGACGCATATTTTTCAGTTCCCTTAGATGAAAGCTTTAGAAAATATACTGCATTCACCATACCTAGTACAAACAATGAGACACCAGGGATTAGATATCAGTACAATGTGCTTCCACAGGGATGGAAAGGATCACCAGCAATATTCCAGAGTAGCATGACAAAAATCTTAGAGCCCTTCAGAGCAAAAAATCCAGAAATAATTATCTATCAATACATGGATGACTTGTATGTAGGATCTGATTTAGAAATAGGACCGCATAGGGCAAAAATAGAAGAATTGAGAGCTCATCTATTGAGCTGGGGATTAACTACACCAGACAAGAAGCATCAGAAAGAACCTCCATTCCTTTGGATGGGTTATGAAC  SUBTYPE A:SUS |
| 079 | AATTTGTAAAGAGATGGAGAAGGAAGGAAAAATTTCAAAAATTGGGCCTGAAAATCCATACAATACTCCAGTATTTGCTATAAAGAAAAAAGATAGCACTAAATGGAGAAAATTAGTAGATTTTAGAGAGCTCAATAAAAGAACTCAGGACTTCTGGGAAGTTCAATTAGGAATACCACATCCCGCAGGTTTAAAAAAGAAAAAATCAGTAACAGTACTAGATGTGGGAGACGCATATTTTTCAGTTCCTTTAGATGAAAACTTTAGAAAGTATACAGCATTCACCATACCTAGTATAAACAATGAGACACCAGGAATCAGGTATCAGTACAATGTGCTTCCACAGGGATGGAAAGGATCACCAGCAATATTCCAGAGTAGCATGACAAAAATCTTAGAGCCCTTCAGATCACAAAATCCAGAAATAATTATCTATCAATACATGGATGACTTGTATGTAGGATCTGATTTAGAAATAGGGCAGCATAGAGCAAAAGTAGAGGAGTTGAGAAGTCATCTATTGAAGTGGGGATTTACCACACCAGACAAAAAGCATCAGAAAGAACCCCCATTTCTTTGGATGGGTT  SUBTYPE A:SUS |
| 080 | CATTAACAGAAATTTGTGCAGATATGGAAAAGGAAGGAAAGATTTCAAAAATTGGGCCTGAGAATCCATACAATACTCCAATATTTGCTATAAAGAAAAAAGACAGCACTAAATGGAGAAAATTAGTAGATTTCAGAGAACTTAATAAAAGAACTCAAGACTTTTGGGAAGTTCAATTAGGAATACCGCATCCAGCGGGGTTAAAAAAGAAAAAATCAGTAACAGTACTAGATGTAGGGGACGCATATTTTTCAGTTCCCCTAGATGAAAGCTTTAGAAAGTATACAGCATTCACAATACCTAGTACAAATAATGAGACACCAGGAATCAGATATCAGTACAATGTGCTCCCACAGGGATGGAAAGGATCACCAGCAATATTCCAGAGCAGCATGACAAAAATCTTAGAGCCCTTTAGATCAAAAAATCCAGAAATAATTATCTATCAATACATGGATGACTTATATGTGGGATCTGATTTAGAAATAGGGCAGCATAGAGCAAAAATAGAAGAGTTAAGAGCTCATCTATTGAGATGGGGACTTACTACACCAGATAAAAAGCATCAGAAAGAACCTCCATTTCTTTGGATGGGTT  SUBTYPE A:SUS |
| 081 | AATTTGTAAAGAGATGGAGAAGGAAGGAAAAATTTCAAAAATTGGGCCTGAAAATCCATACAATACTCCAGTATTTGCTATAAAGAAAAAAGATAGCACTAAATGGAGAAAATTAGTAGATTTTAGAGAGCTCAATAAAAGAACTCAGGACTTCTGGGAAGTTCAATTAGGAATACCACATCCCGCAGGTTTAAAAAAGAAAAAATCAGTAACAGTACTAGATGTGGGAGACGCATATTTTTCAGTTCCTTTAGATGAAAACTTTAGAAAGTATACAGCATTCACCATACCTAGTATAAACAATGAGACACCAGGAATCAGGTATCAGTACAATGTGCTTCCACAGGGATGGAAAGGATCACCAGCAATATTCCAGAGTAGCATGACAAAAATCTTAGAGCCCTTCAGATCACAAAATCCAGAAATAATTATCTATCAATACATGGATGACTTGTATGTAGGATCTGATTTAGAAATAGGGCAGCATAGAGCAAAAGTAGAGGAGTTGAGAAGTCATCTATTGAAGTGGGGATTTACCACACCAGACAAAAAGCATCAGAAAGAACCCCCATTTCTTTGGATGGGTT  SUBTYPE A:SUS |
| 082 | GTTAAAACAATGGCCATTGACAGAAGAAAAAATAAAAGCATTAACAGAAATTTGTACAGAAATGGAAAAGGAAGGAAAAATTTCAAAAATTGGGCCTGAGAATCCATACAATACCCCAATATTTGCTATAAAGAAAAAAGACAGTACT:AAGTGGAGAAAATTAGTGGATTTTAGAGAACTTAATAAGAGAACTCAAGATTTCTGGGAAGTTCAATTAGGAATACCACATCCTGCAGGATTAAAAAAGAAAAATTCAGTAACAGTACTGGATGTGGGTGATGCATATTTTTCAGTTCCCTTAGATGAAGACTTTAGAAAATATACCGCATTCACTATACCTAGTATAAATAACGAGACACCAGGAGTTAGATATCAGTACAACGTGCTTCCACAAGGATGGAAAGGGTCACCATCAATATTTCAAAGTAGCATGACAAAGATCTTAGAACCTTTTAGAAAACAAAATCCAGAAATAGTTATCTATCAATACATGGATGATTTGTATGTAGGATCTGACTTAGAAATAGGGCAGCATAGAACAAAAATAGAGGAATTAAGGGGACACCTATTGAAGTGGGGATTCACCACACCAGACAAAAAGCATCAGAAAGAACCTCCATTTCTTTGGATGGGTTATGAACTACACCCTGA  SUBTYPE D:SUS |
| 083 | TGGCCATTGACAGAAGAAAAAATAAAAGCATTAACCAGCAATTTGTGAAGATATGGAGARGGAAGGAAAAATTACAAGAGTTGGGCCTGAGAATCCATATAACACTCCAATATTTGCCATAAAAAAGAAAGATAGTACTAAGTGGAGAAAATTAGTAGACTTTAGGGAACTCAATAAAAGAACTCAAGACTTTTGGGAAGTCCAATTAGGGATACCACACCCAGCAGGGTTAAAAAAGAAAAAATCAGTGACAGTACTGGATGTGGGGGATGCATATTTYTCAGTTCCTTTAGATGAGAGYTTTAGRAAATATACTGCATTCACCATACCTAGTACAAATAATGAAACACCAGGAATTAGATATCAATACAATGTRCTTCCACAGGGATGGAAAGGATCACCAGCGATATTTCAGAGTAGTATGACAAAAATCTTAGAGCCCTTTAGGGCACAAAACCCAGAAATAGTTATCTATCAATATATGGATGACTTGTATGTAGGATCTGACTTAGAAATAGGGCAACATAGGGCAAAAATAGAGGAGTTAAGAGAACATCTATTAAGGTGGGGATTTACCACACCAGACAAAAAGCATCAGAAAGAACCTCCATTTCTTTGGATGGGTTATGAACTABATCCTGA  SUBTYPE C:SUS |
| 084 | CCAAAAGTTAAACAATGGCCATTGACAGAAGAAAAGATAAAAGCATTAACAGAAATTTGTAAAGAAATGGAAGCTGAAGGAAAAATTTCAAAAATTGGGCCTGAAAATCCATACAATACTCCAATATTTGCTATAAAGAAAAAAGATAGCACTAAGTGGAGAAAATTGGTAGACTTTAGAGAGCTCAATAAAAGAACTCAGGACTTCTGGGAAGTTCAATTAGGAATACCGCATCCCGCGGGGTTAAAAAAGAAAAAATCAGTAACAGTACTAGATGTGGGGGATGCATATTTTTCAGTTCCTTTACATGAGAGCTTTAGAAAATATACTGCATTCACCATACCTAGTATAAACAATGAGACACCAGGAATTAGATATCAGTACAATGTGCTGCCACAAGGATGGAAAGGGTCACCGGCAATATTCCAGAGTAGCATGACAAAAATCTTAGAGCCATTTAGAGCAAAAAATCCAGAAATGRTTATCTATCAATACRTGGATGACCTGTATGTAGGATCTGATTTAGAAATAGGGCAGCATAGAGAAAAAATAGAACAATTAAGAGCTCACTTATTGAAATGGGGATTTACTACACCAGACAAAAAGCATCAGAAAGAACCTCCATTCCTTTGGATGGGWTATGARCTA  Subtype A  **M184MV** |
| 085 | AACAATGGCCATTGACAGAAGAAAAAATAAAAGCACTAACAGAAATTTGTACAGAAATGGAAAAGGAAGGAAAAATCTCAAGAATTGGGCCTGAAAATCCATACAATACTCCAATATTTGCCATAAAGAAAAAAGACAGTACTAAGTGGAGAAAATTAGTAGATTTCAGRGAACTTAATAARAGGACTCAAGACTTCTGGGAAGTTCAACTAGGAATACCACATCCWGCAGGGTTAAAAAAGAAAAAATCAGTAACAGTACTGGATGTGGGKGATGCATATTTTTCAGTTCCCTTATATGAAGACTTTAGAAAGTATACTGCATTCACCATACCTAGTATAAACAATGAGACACCAGGAATTAGATATCAATACAATGTGCTTCCACAAGGATGGAAAGGATCACCAGCAATATTCCAAAGTAGCATGACAAAAATCTTAGAACCTTTTAGAMRACAAAATCCAGAAATAGTTRTCTATCAATACATGGATGATTTGTAYGTAGGATCTGATTTAGACATAGGGCAGCACAGAAYAAAAATAGAGGAATTAAGAGAACACCTCTTGAAGTGGGGATTTACCACGCCAGATAAAAAGCATCAGAAAGAACCCCCATTTCTTTGGATGGGTTATGAACTA  SUBTYPE D:SUS |
| 086 | ARAAAAWAAAAAGCATTAACAGAAATTTGTGCAGATATGGAAAAGGARGGAAAAATTTCAAGAATTGGGCCTGAAAATCCATACAATACTCCAATATTTGCTATAAAGAAAAAAGACAGTACYAAATGGAGRAAATTAGTRGATTTCAGRGAACTYAATAARAGAACTCAAGATTTYTGGGARGTYCAATTAGGAATACCACATCCTGCRGGGYTRAAAAAGAAAAAATCAGTAACAGTAYTGGATGTRGGKGATGCATATTTTTCAGTTCCCTTATATGAARAYTTYAGAAAATATACTGCATTCACCATACCTAGTATAAACAATGAGACACCAGGARTYAGRTATCAATACAATGTGCTWCCACAGGGATGGAAAGGATCACCAGCAATATTCCAGAGTAGCATGACAAAAATCTTGGAGCCCTTTAGAAAACAAAAYCCAGAMATARTTATCTATCAATACATGGATGATTTGTATGTAGGATCTGAYTTRGAAATAGGGCAGCATAGAACAAAAATAGATGAACTAAGAGAACATCTATTGAAGTGGGGATTTACCACACCAGATAAAAAACATCAGAAAGAACCTCCATTTCTTTGGATGGGTTATGAACTACATCCTGA  SUBTYPE D:SUS |
| 087 | ACAATGGCCATTGACAGAAGAGAAAATAAAAGCATTAACAGCAATTTGTGAAGATATGGAAAAGGAAGGAAAAATTTCAAGAATTGGGCCTGAAAATCCATATAACACTCCAGTATTTGCCATAAAAAAGAAGGACAGTACTAAGTGGAGAAAATTAGTAGATTTCAGGGAGCTCAATAAAAGAACTCAAGACTTTTGGGAAGTTCAATTAGGGATACCACACCCAGCAGGGTTAAAGAAGAAAAAATCAGTGACAGTATTGGATGTGGGGGATGCATATTTCTCAGTACCTTTAGATGAAAACTTCAGGAAGTAYACAGCATTCACCATACCTAGTATAAACAATGAAACACCAGGAATTAGATATCAATATAATGTGCTTCCACAGGGATGGAAAGGATCACCATCAATATTTCAGAGTAGCATGACAAAAATCTTAGAGCCCTTTAGGATAAAAAACCCAGACATAGTTATCTATCAATATATGGATGATTTGTATGTAGGATCTGATTTAGAAATAGGGCAACATAGAGCAAAAATAGAGGAGTTAAGGGATCATCTATTGAAGTGGGGATTTACTACACCAGACAAGAAACATCAGAAAGAACCTCCATTTCTTTGGATGGGTTTA  SUBTYPE C:SUS |
| 088 | CAATGGCCATTGACAGAAGAAAAAATAAAAGCATTAACAGAAATTTGTACAGAAATGGAAAAGGAAGGAAAAATTTCAAAAATTGGSCCTGAAAAYCCATACAATACTCCAATATTTGCTATAAAGAAAAAAGAYAGYACTAAATGGAGGAAATTAGTGGACTTCAGAGAACTCAATAAAAGAACTCAAGAYTTTTGGGAAGTTCARTTAGGAATACCACAYCCAGCGGGCTTAAAAAAGAAAAAATCAGTAACAGTACTAGATGTGGGGGAYGCATATTTTTCAGTTCCCTTAGATGAAARCTTTAGAAAGTATACTGCATTCACCATACCTAGTAYAAACAATGAGACACCAGGAATCAGATATCAGTACAATGTGCTTCCGCAGGGATGGAARGGATCACCRGCAATATTCCAGAGTAGCATGACAAAAATCTTAGAGCCCTTTAGATCAAAGAACCCAGAAATAATTATCTATCAATACATGGATGACTTGTATGTAGGATCTGATTTGGAAATAGGGCAACATAGAACAAAAGTAGAAGAGTTGAGAGATCATCTATTGAGCTGGGGATTTACTACACCAGACAAAAAGCATCAGAAAGAACCTCCATTTCTTTGGATGGGTTATGAACTA  SUBTYPE A:SUS |
| 089 | ACAATGGCCATTGACAGAAGAAAAAATAAAAGCATTAGTAGAAATTTGTACAGAAATGGAAAAAGAAGGAAAAATTACAAAAATTGGGCCTGAAAATCCATACAATACTCCAATATTTGCTATAAAGAAAAAGGACAGCACTAAATGGAGGAAACTAGTAGATTTCAGAGAGCTCAATAAAAGGACACAAGACTTTTGGGAAGTTCAATTAGGGATACCGCATCCAGCGGGCCTAAAAAAGAAAAAATCAGTAACAGTACTGGATGTGGGGGATGCATATTTTTCAGTCCCTTTAGATGAAAGCTTTAGGAAATATACTGCGTTCACCATACCTAGTACAAACAATGAGGCACCAGGAATTAGATATCAATACAATGTGCTTCCACAAGGATGGAAAGGATCACCAGCAATATTCCAAAGTAGCATGACAAAAATCTTAGAACCTTTTAGAAAGCAAAATCCAGAAATAGTTATCTATCAATACATGGATGACTTGTATGTCGCATCTGACTTAGAAATAGGGCAACATAGAACAAAGATAGAAGAATTAAGGGAACACCTATTGAAATGGGGATTTACCACACCAGACAAAAAGCATCAGAAAGAACCTCCATTKCTKTGGATGGGTTATGAACTA  Subtype B  **G190A**, **F227FL** |
| 090 | TAAACAATGGCCATTGACAGAAGAAAAAATAAAAGCATTAACAGAAATTTGTATGGAAATGGAAAAGGAGGGAAAAATTTCAAAAATTGGGCCTGAAAATCCATACAATACTCCAATATTTGCAATAAGGAAAAAAGACAGCACTAAATGGAGAAAATTAGTAGATTTCAGAGAGCTCAATAAAAGAACACAAGACTTTTGGGAGGTTCAATTAGGAATACCACATCCAGCTGGCCTAAAAAARAAAAAATCAGTAACAGTACTAGATGTGGGGGACGCATATTTTTCAGTTCCTTTACATGAAGATTTTAGGAAGTATACTGCGTTCACCATACCTAGTACAAACAATGAGACACCAGGAATCAGATATCAGTACAATGTGCTCCCACAGGGATGGAAAGGATCACCGTCAATATTCCAGGCTAGCATGACAAAAATCTTAGAGCCCTTTAGATCAAAAAATCCAGAGCTAGTTATKTATCAGTACATGGATGACTTGTATGTAGGATCTGATTTAGAAATAGGGCAGCACAGAGCAAAAATAGAAGAGCTGAGAGCTCATCTATTGAGCTGGGGATTTACTACACCAGACAAAAAGCATCAGAAAGAACCTCCATTCCTCTGGATGGGATATGAGCTA  SUBTYPE A:SUS |
| 091 | AACAATGGCCATTGACAGAAGAAAAAATAAAAGCACTAACAGAAATTTGTATAGACATGGAAAAGGAAGGAAAAATTTCAAGAATTGGGCCTGAAAATCCATACAATACTCCAATATTTGCTATAAAGAAAAAAGACAGTACCAAGTGGCGAAAATTAGTAGATTTCAGAGAACTTAATAAGAGAACTCAAGATTTCKGGGAAGTTCAACTAGGAATACCACATCCTGCAGGGCTAAAAAAGAAAAAATCAGTTACAATACTGGATGTGGGTGATGCATATTTTTCAGTTCCCTTGGATAAAGAATTTAGAAAATACACTGCATTCACCATACCTAGTATAAACAATGAGACACCAGGAATTAGATATCAGTATAATGTGCTTCCACAAGGGTGGAAAGGATCACCAGCAATATTCCAAAGTAGCATGACAAAAATCTTAGAGCCCTTTAGGAAACAAAATCCAGAAATAGTTATCTRTCAATACATGGATGACTTGTATGTAGGGTCTGACTTAGAAATAGGGCAGCATCGAGCAAAAATAGAACAGTTGAGAGCTCATCTATTGAGATGGGGATTTAMTACACCAGACAAGAAGCATCAGAAAGAACCTCCATTTCTTTGGATGGGTTATGAACTA  SUBTYPE D:RES NNRTI/NRTI |
| 092 | GTTAAACAATGGCCATTGACAGCAGAAAAAATAAAAGCATTAACAGAAATTTGTTTAGAAATGGAAAAGGAAGGAAAAATTTCTAAAATTGGGCCTGAAAATCCATACAATACTCCAGTATTTGCAATAAAGAAAAAAGATAGCACTAAATGGAGAAAATTAGTAGATTTCAGAGAGCTCAATAAAAGAACACAAGACTTTTGGGAAGTTCAATTAGGAATACCGCATCCAGCGGGMCTAAAAAAGAAAAAATCAGTAACAGTACTAGATGTGGGGGATGCATATTTTTCTGTCCCCTTAGATGAAGAATTTAGAAAGTATACTGCATTCMCCATACCTAGTACAAACAATGAGACACCAGGAATCAGGTATCAATACAATGTGCTTCCACAGGGATGGAAAGGATCACCGGCAATATTCCAGAGTAGCATGACAAAAATCTTAGAGCCCTTTAGATCAAAGAATCCAGAGATAATTATCTATCAATACATGGATGACTTGTATGTAGGATCTGATTTAGAAATAGGGCAACATAGAGCAAAAATAGAAGAGTTGAGAGCTCATCTATTGAGCTGGGGATTTACTACACCAGACAAAAAGCATCAGAAAGAACCTCCATTCCTTTGGATGGGGTATGAACTA  SUBTYPE A:SUS |
| 093 | CAATGGCCATTGACAGAAGAAAAAATAAAAGCACTAACAGAAATTTGTATGGAAATGGAAAAGGAAGGAAAAATTTCAAAAATTGGGCCTGAAAATCCATACAACACTCCAGTATTTGCTATAAAGAAAAAAGACAGCACTAAGTGGAGAAAATTAGTAGATTTCAGAGAGCTCAATAAAAGAACTCAAGACTTTTGGGAAGTTCAGTTAGGAATACCACACCCAGCAGGGTTAAAAAAGAAAAAGTCAGTGACAGTATTGGATGTGGGGGATGCATATTTTTCAGTTCCTTTAGATGAAGGATTCAGGAAATATACTGCATTCACCATACCTAGTACAAACAATGAGACACCAGGAATTAGATATCAGTACAATGTGCTTCCACAAGGATGGAAAGGATCACCGGCAATATTCCAAAGTAGCATGACAAGAATCTTAGAACCTTTTAGAAAACAAAATCCAGAAATAGTTATCTATCAATACATGGATGATTTGTATGTAGGATCTGACTTAGAAATAGAGCAACATAGAGCAAAAATAGAGGAATTAAGGGAACACCTATTGAAGTGGGGGTTTACCACACCAGATAAAAAGCATCAGAAAGAACCTCCATTTCTTTGGATGGGTTATGAACTA  SUBTYPE B:SUS |
| 094 | AAACAATGGCCATTGACAGAAGAAAAAATAAAAGCATTAACAGAAATTTGTACAGAAATGGAGAAGGAAGGAAAAATTTCAAAAATCGGGCCTGAAAATCCATACAATACTCCAATATTTGCTATAAAGAAAAAGGACAGCACTAAGTGGAGAAAATTAGTAGATTTTAGAGAGCTCAATAAAAGAACTCAAGATTTTTGGGAAGTCCAATTAGGAATACCGCATCCAGCGGGCCTAAAAAAGAAAAAATCAGTAACAGTACTGGATGTGGGGGATGCATATTTCTCAGTTCCTTTAGATGAAAGCTTTAGAAAGTATACTGCGTTCACCATACCTAGTACAAACAATGAGACACCAGGAATCAGGTATCAGTACAATGTGCTTCCACAGGGATGGAAGGGATCACCAGCAATATTCCAAAGTAGCATGACAAAAATCTTAGAGCCTTTTAGATCAAAAAATCCAGAAATAATTATCTATCAGTACATGGATGACTTATATGTAGGATCTGATTTAGAAATAGGGCAGCATAGAGAAAAAATAGAGGAGTTGAGAGCTCATCTATTGAGCTGGGGACTTACTACCCCGGACAAAAAGCATCAGAAAGAACCGCCATTTCTTTGGATGGGTTATRAACTA  SUBTYPE A:SUS |
| 095 | ATGGCCATTGACAGAAGAAAAAATAAAAGCATTAACAGAAATTTGTAAAGAGATGGAAAAGGAAGGAAAAATTTCAAAAATCGGGCCTGAAAATCCATACAATACTCCAATATTTGCTATAAAGAAAAAAGATAGCACTAAATGGAGAAAATTAGTAGATTTTAGAGAGCTCAATAAAAGAACTCAGGACTTCTGGGAAGTTCAATTAGGAATACCACATCCAGCAGGTTTAAAAAAGAAAAAATCAGTAACAGTACTAGATGTGGGGGACGCATATTTTTCAGTTCCTTTAGATGAAAACTTTAGAAAGTATACTGCGTTCACCATACCTAGTATAAACAATGAGACACCAGGAATCAGGTATCAATACAATGTGCTCCCGCAGGGATGGAAAGGATCACCAGCAATATTCCAAAGTAGCATGACAAAAATCTTAGAGCCCTTTAGAGCAAAAAATCCAGAAATAATTATCTATCAATATATGGATGACTTGTATGTAGGATCTGATTTAGAAATAGGACAGCATAGARCAAAAGTAGAGGAATTGAGAGCTCATCTATTGAGCTGGGGATTTACTACACCAGACAAAAAGCATCAGAAAGAACCTCCATTTCTTTGGATGGGTTATGAACTA  SUBTYPE A:SUS |
| 096 | AACAATGGCCATTGACAGAAGAAAAGATAAAAGCATTAACAGAAATTTGTAAAGAAATGGAAGCTGAAGGAAAAATTTCAAAAATTGGGCCTGAAAATCCATACAATACTCCAATATTTGCTATAAAGAAAAAAGATAGCACTAAGTGGAGAAAATTGGTAGACTTTAGAGAGCTCAATAAAAGAACTCAGGACTTCTGGGAAGTTCAATTAGGAATACCGCATCCCGCGGGGTTAAAAAAGAAAAAATCAGTAACAGTACTAGATGTGGGGGATGCATATTTTTCAGTTCCTTTACATGAGAGCTTTAGAAAATATACTGCATTCACCATACCTAGTATAAACAATGAGACACCAGGAATTAGATATCAGTACAATGTGCTGCCACAAGGATGGAAAGGGTCACCGGCAATATTCCAGAGTAGCATGACAAAAATCTTAGAGCCATTYAGAGCAAAAAATYCAGAAATGRTTATCTATCAATACRTGGATGACCTGTATGTAGGATCTGATTTAGAAATAGGGCAGCATAGAGAAAAAATAGAACAATTAAGAGCTCACTTATTGAAATGGGGATTTACTACACCAGACAAAAAGCATCAGAAAGAACCTCCATTCCTTTGGATGGGTTATGAACTA  Subtype A  **M184MV** |
| 097 | AAAAAATAAAAGCATTAACTGAAATTTGTACAGAAATGGAAAAGGAAGGAAAAATTTCAAAAATTGGGCCTGAAAATCCATACAATACTCCAATATTTGCAATAAGGAAAAAAGATAGTACTAAATGGAGGAAATTAGTGGATTTCAGAGAGCTCAATAAAAGAACACAAGATTTTTGGGAAGTTCAATTAGGGATACCACATCCAGCGGGCCTAAAGAAGAAYAAATCAGTAACAGTACTAGATGTGGGGGATGCATATTTTTCAGTTCCTTTACATGAAGACTTTAGAAAATATACTGCGTTCACCATACCTAGTACAAACAATGAGACACCAGGAATCAGATATCAGTACAATGTGCTACCACAGGGATGGAAAGGATCACCAGCAATATTCCAGAGTAGCATGACAAAAATCTTAGAGCCCTTTAGATCAAAAAATCCAGAAATAAGCATCTATCAATACATGGATGACTTGTATGTAGGATCTGATTTAGAAATAGGGCAACATAGAGCAAAAATAGAGGAGTTAAGAGCTCATCTATTGAGCTGGGGGTTTACTACACCAGACAAAAAGCATCAGAAAGAACCTCCATTCCTTTGGATGGGTTATGAACTACATCCTGACAAGTGGACAGTCCAGCCTATAAAGCTGCCA  Subtype A  **K103N** |
| 098 | AAGAAAAAATAAAAGCATTAACAGATATTTGTACAGAAATGGAAAAGGAAGGAAAAATTTCAAGAATTGGGCCTGAAAATCCATATAATACACCAATATTTGCCATAAAGAAAAAAGATAGTACTAAGTGGAGAAAATTAGTAGATTTCAGAGAACTTAATAAGAGAACTCAAGACTTCTGGGAAGTTCAACTAGGAATACCACATCCTGCAGGGCTAAAAAAGAAAAAATCAGTAACAGTACTGGATGTGGGTGATGCATATTTTTCAGTGCCCTTATGTGAAGACTTTAGAAAATATACTGCATTCACCATACCTAGTATAAACAATGAAACACCAGGAATTAGATATCAGTACAATGTGCTTCCACAAGGCTGGAAAGGATCACCGGCAATATTCCAAAGTAGCATGACAAAGATCTTAGAACCTTTTAGAAAACAAAACCCGGAAATGGTTATTTATCAATACATGGATGATTTGTATGTAGGATCTGATTTAGAAATAGGGCAGCATAGAACGAAAATAGAAGAATTAAGGGAGCACCTATTGAAGTGGGGCTTTACCACACCAGACAAAAAGCATCAGAAAGAACCTCCATTTCTTTGGATGGGTTATGAACTAYAAYCCTG  SUBTYPE D:SUS |
| 099 | CAATGGCCATTGACAGAAGAAAAAAWWAAAAGCACTAAACAGAAATTTGTACAGATATGGAAAAGGAAGGAAAAATTTCAAGAATTGGGCCTGAAAATCCATACAATACTCCAATATTTGCCATAAAGAAAAAAGACAGTACTAAGTGGAGAAAATTAGTAGATTTTAGAGAACTTAATAAAAGAACTCAAGACTTCTGGGAAGTTCAACTAGGAATACCACATCCTGCAGGGCTAAAAAAGAAAAAATCAGTAACAGTACTGGACGTGGGTGATGCATATTTTTCAGTTCCCTTAGATGAAAACTTTAGAAAATATACCGCATTCACCATACCCAGTATAAATAATGAGACACCAGGAATTAGATACCAGTACAATGTGCTTCCACAAGGATGGAAAGGATCACCAGCAATATTCCAAAGCAGCATGACAAAAATCCTAGAACCTTTTAGGAAAMMAAAATCCAGAAATGGTTATCTATCAATACATGGATGATTTGTATGTAGGATCTGACTTAGAAATAAGGGCAGCATAGAAACAAAGATAGAGGAACTAARGGAAMCMCTTATTGAAGTGGGGGGTTTACCACACCAGACAAAAAGCATCARAAAGAACCTCCATTTCTTTGRAKGGRKTAWKAAAMTAA  SUBTYPE D:SUS |
| 100 | AAAWAAAAAGCATTAACAGAAATATGTAGGGAGATGGAAGAAGAAGGAAAAATTACAAAAATTGGGCCTGAAAATCCATATAACACTCCAGTATTTGCYATAAAAAAGAAGGACAGTACTAAGTGGAGAAAATTAGTAGACTTCAGGGAACTCAATAAAAGAACTCAAGACTTTTGGGAAGTTCAATTAGGAATACCACACCCAGCAGGATTAAAAAGGAACAAATCAATAACGGTACTGGATGTGGGAGATGCATATTTTTCAGTTCCTTTAGATGAAGATTTCAGRAAATACACTGCATTCACCATACCTAGTATAAACAATGAAACACCAGGAGTTAGATACCAATATAATGTGCTTCCACAAGGATGGAAAGGATCACCAGCAATATTCCAGAGTAGTATGACAAGAATCTTAGAGCCCTTTAGAGCAAGAAACCCAGAGATAGTTATCTATCAATATATGGATGACTTGTATGTAGGATCTGATTTAGAACTAAAGCAACATAGAGCAAAAATAGAAGAGTTAAGAGAACACCTATTGAAATGGGGATTTACCACACCAGACAAGAAACATCAGAAAGAACCCCCATTTCTTTGGATGGGTTATGAACTACATCCTGA  Subtype C  **K103N**, **V106I** |
