# Supplementary material for: An Android-Based Mobile App (ARVPredictor) for the Detection of HIV Drug-Resistance Mutations and Treatment at the Point of Care: Development Study
Source: JMIR Form Res. 2022 Feb 2;6(2):e26891. doi: 10.2196/26891 (PMC8851341; doi:10.2196/26891)
Supplement: Multimedia Appendix 3 [file formative_v6i2e26891_app3.docx]

|  |  |  |  | |  | |  | |  | |  |  |  | |  | |  | |  | |
| --- | --- | --- | --- | --- | --- | --- | --- | --- | --- | --- | --- | --- | --- | --- | --- | --- | --- | --- | --- | --- |
|  |  |  |  | |  | |  | |  | |  |  |  | |  | |  | |  | |
|  | **Stanford HIV Database** | | | | | | | | | | **©ARVPredictor** | | | | | | | | | |
| **SeqID** |  |  | | **RT Mutation Output** | | | | | | |  |  | | **RT Mutation Output** | | | | | |  |
|  | **HIV Subtype** | **Susceptibility** | | **NNRTI** | | **NRTI** | | | |  | **HIV Subtype** | **Susceptibility** | | **NNRTI** | | **NRTI** | | | |  |
|  |  |  | |  | | **TAMs** | | **Non-TAMs** | | **Other Mutations** |  |  | |  | | **TAMs** | | **Non-TAMs** | | **Other Mutations** |
| 1 | A | Susceptible | | **V179T** | | None | | None | | E28EA, V35T, E40D, V60I, K122E, D123G,  F214L | A | Susceptible | | **V179T** | | None | | None | | SAME |
| 2 | A | Susceptible | | **V179T** | | None | | None | | V35T, T39M, V60I, D121H, K122E, D123G, R125RK, R211S | A | Susceptible | | **V179T** | | None | | None | | SAME |
| 3 | A | Susceptible | | None | | None | | None | | V35T, T39M, V60I, D121H, K122E, D123G, I135T, A158S, T211S | A | Susceptible | | None | | None | | None | | SAME |
| 4 | A | Susceptible | | None | | None | | None | | V35T, V60I, K122E, D123S, I135T, K173S, Q174K, D177E, T200A, Q207A, R211S | A | Susceptible | | None | | None | | None | | SAME |
| 5 | B | Susceptible | | None | | None | | None | | E40D, I47L, K49R, V60I, D121C, K122E, D123E, R125RK, K173E, D177E, T200A, Q207E | B | Susceptible | | None | | None | | None | | SAME |
| 6 | A | **NNRTI** | | **K103KN** | | None | | None | | K32KN, V35T, K43E, V60I, K122E, D123G, I135T, K173I, Q174K, V179I, T200A, Q207A, R211S | A | **NNRTI** | | **K103KN** | | None | | None | | SAME |
| 7 | D | Susceptible | | None | | None | | None | | V35T, E40D, K49R, V60I, D121Y, K122E, Q174R, D177E, I178V, T200I, Q207E, R211K | D | Susceptible | | None | | None | | None | | SAME |
| 8 | B | Susceptible | | None | | None | | None | | V35I, K49R, K102KE, K122E, D123S, S162A, D177E, I178M, V179I, T200A, E204K, Q207E, R211K | B | Susceptible | | None | | None | | None | | SAME |
| 9 | B | **NNRTI, NRTI** | | **V106VA, F227FL, M230I** | | None | | **K65KR** | | V35I, K49R, K102KE, K122E, D123S, I135IV, S162A, D177E, I178M, V179I, T200A, E204EK, Q207E, R211K | C+D | **NNRTI, NRTI** | | **V106VA**, **F227FL** | | None | | **K65KR** | | **M2301** |
| 10 | C + D | **NNRTI, NRTI** | | **V106A** | | None | | **M184V** | | V35I, K49R, K102E, K122E, D123S, S162A, T165K, D177E, I178M, V179I, T200A, Q207G, R211K | C+D | **NNRTI, NRTI** | | **V106A** | | None | | **M184V** | | SAME |
| 11 | C | **NNRTI, NRTI** | | **K103N** | | None | | **M184V** | | V35T, E36A, T39E, S48T, K122E, D123N, I135IT, T139TK, K173A, D177E, I178M, T200A, Q207N, R211K | C | **NNRTI, NRTI** | | **K103N** | | None | | **M184V** | | SAME |
| 12 | C | **NNRTI, NRTI** | | **K103N** | | None | | **M184V** | | L34I, V35T, E36A, T39E, S48T, D86DE, K122E, D123N, T139K, E169EK, K173A, D177E, I178M, T200A, Q207N, R211K | C | **NNRTI, NRTI** | | **K103N** | | None | | **M184V** | | SAME |
| 13 | B | Susceptible | | None | | None | | None | | V35T, T39M, V60I, D121Y, K122E, D123DN, R125RK, Q174K, D177E, I178M, Q207E | B | Susceptible | | None | | None | | None | | SAME |
| 14 | D | **NNRTI, NRTI** | | **A98G**, **Y181C**, **H221HY** | | None | | **M184V** | | V35T, E40D, K43R, K49R, V60I, D121Y, K122E, D123E, I135T, D177E, I178M, I195L, T200I, E203EK, E204K, Q207E, R211K | D | **NNRTI, NRTI** | | **A98G**, **Y181C** | | None | | **M184V** | | **H221HY** |
| 15 | A | Susceptible | | None | | None | | None | | V35T, V60I, D121H, K122E, D123S, K173S, Q174K, D177E, T200A, Q207AT, R211S | A | Susceptible | | None | | None | | None | | SAME |
| 16 | A | Susceptible | | None | | None | | None | | V35T, T39A, E40D, V60I, D121H, K122E, I135T, K173S, Q174K, D177Q, V179I, T200A, Q207A, R211S | A | Susceptible | | None | | None | | None | | SAME |
| 17 | A | Susceptible | | None | | None | | None | | V35T, T39N, V60I, K122E, D123S, S162A, K173S, Q174K, D177E, T200I, I202V, Q207D, R211K | A | Susceptible | | None | | None | | None | | SAME |
| 18 | A | Susceptible | | None | | None | | None | | V35T, T39N, V60I, K122E, D123S, S162A, K173S, Q174K, D177E, T200I, I202V, Q207D, R211K | A | Susceptible | | None | | None | | None | | SAME |
| 19 | A2 | **NNRTI, NRTI** | | **Y188L** | | None | | **M184V** | | V35T, T39L, D123E, D177E, I178M, T200A, Q207E, R211K | A2 | **NNRTI, NRTI** | | **Y188L** | | None | | **M184V** | | SAME |
| 20 | A | Susceptible | | None | | None | | None | | V35T, V60I, D123E, I135T, S162N, T165L, K173S, Q174K, D177E, V179I, G196E, T200S, I202V, Q207A, F214L | A | Susceptible | | None | | None | | None | | SAME |
| 21 | D | Susceptible | | None | | None | | None | | V35T, T39M, K49R, V60I, D121Y, K122E, K173R, Q174K, D177E, T200I, Q207E, R211K | D | Susceptible | | None | | None | | None | | SAME |
| 22 | D | Susceptible | | None | | None | | None | | V35T, T39M, K49R, V60I, D121Y, K122E, K173R, Q174K, D177E, T200I, Q207E, R211K | D | Susceptible | | None | | None | | None | | SAME |
| 23 | D | **NNRTI** | | **G190GA** | | None | | None | | V35T, E36D, T39N, V60I, K122E, D177E, I178M, T200I, Q207E, R211K | D | **NNRTI** | | **G190GA** | | None | | None | | SAME |
| 24 | A | Susceptible | | None | | None | | None | | V35T, V60I, I135T, K173L, Q174K, D177E, V179I, Q207A, R211S | A | Susceptible | | None | | None | | None | | SAME |
| 25 | A | **NNRTI** | | **K103N** | | None | | None | | K32R, V35PT, V60I, K122E, D123S, I135T, A158S, K173A, Q174K, D177E, V179I, I202IV, Q207A, R211S | A | **NNRTI** | | **K103N** | | None | | None | | SAME |
| 26 | A | **NNRTI, NRTI** | | **K103N** | | None | | **M184V** | | K32R, V35T, V60I, K122E, D123S, I135T, A158S, K173A, Q174K, D177E, V179I, Q207A, R211S | A | **NNRTI, NRTI** | | **K103N** | | None | | **M184V** | | SAME |
| 27 | A | **NNRTI** | | **G190A** | | None | | None | | V35T, V60I, K122E, D123S, I135T, I142V, F171Y, K173S, Q174K, D177E, V179I, T200A, Q207A, R211S | A | **NNRTI** | | **G190A** | | None | | None | | SAME |
| 28 | A | **NNRTI, NRTI** | | **E138Q**, **G190A** | | None | | **M184V** | | V35T, V60I, K122E, D123S, I135T, I142V, F171Y, K173S, Q174K, D177E, V179I, T200A, Q207A, R211S | A | **NNRTI, NRTI** | | **E138Q**, **G190A** | | None | | **M184V** | | SAME |
| 29 | D | Susceptible | | None | | None | | None | | V35T, T39KN, V60I, V90VI, K102N, D121CHRY, K122E, I135IATV, I142IV, D177E, I178V, Q197QK, Q207E, R211RK | D | Susceptible | | None | | None | | None | | SAME |
| 30 | A | **NNRTI, NRTI** | | **G190A** | | None | | **M184V** | | V35T, T39E, I142T, T165A, K173S, D177G, V179I, I202V, Q207A, R211S, P226PR | A | **NNRTI, NRTI** | | **G190A** | | None | | **M184V** | | SAME |
| 31 | B | **NNRTI** | | **V179VD** | | None | | None | | V35T, E40ED, V60I, K122E, K173A, D177E, T200A, Q207E, R211RK | B | **NNRTI** | | **V179VD** | | None | | None | | SAME |
| 32 | D | **NNRTI, NRTI** | | **Y181YC** | | None | | **V75VM** | | V35T, T39K, K49R, V60I, K64R, K122E, D123G, I135IMR, D177E, I178M, T200I, E203G, Q207E, R211K | C+D | **NNRTI, NRTI** | | **Y181YC** | | None | | **V75VM** | | SAME |
| 33 | A | Susceptible | | None | | None | | None | | V60I, K122E, D123N, K173T, Q174K, D177E, V179VI, T200A, Q207A, R211S | A | Susceptible | | None | | None | | None | | SAME |
| 34 | B | Susceptible | | None | | None | | None | | V35T, E40D, K49R, V60I, Q174K, D177E, Q207E | B | Susceptible | | None | | None | | None | | SAME |
| 35 | A | Susceptible | | None | | None | | None | | K122E, D123N, K173S, Q174K, D177E, V179I, I202V, Q207A | A | Susceptible | | None | | None | | None | | SAME |
| 36 | A | Susceptible | | None | | None | | None | | K49R, V60L, D121H, K122E, R125RK, I135T, I142V, K173T, Q174K, D177E, Q207A, F214L | A | Susceptible | | None | | None | | None | | SAME |
| 37 | A | **NNRTI** | | **Y181YFIN** | | None | | None | | V35T, V60I, V90VI, K122E, D123S, I135T, I142V, K173S, Q174K, D177E, V179I, Q207A, R211S | A | **NNRTI** | | **Y181YFIN** | | None | | None | | SAME |
| 38 | A | Susceptible | | None | | None | | None | | V35T, T39G, V60I, I135T, K173S, Q174K, Q207A, R211N | A | Susceptible | | None | | None | | None | | SAME |
| 39 | A | Susceptible | | None | | None | | None | | V35T, E40D, V60I, K122E, D123N, I142V, K173S, Q174K, D177E, V179I, Q207A, R211S | A | Susceptible | | None | | None | | None | | SAME |
| 40 | G | **NNRTI** | | **Y181C**, **H221Y** | | None | | None | | V35I, V60I, K122E, D123N, I135V, K173R, Q174E, D177E, I178M, T200A, Q207K, R211N | G | **NNRTI** | | **Y181C** | | None | | None | | **H221Y** |
| 41 | A | Susceptible | | None | | None | | None | | V35T, T39I, V60I, D121H, I135T, K173S, Q174N, D177E, I195L, T200A, Q207A, R211S, F214L | A | Susceptible | | None | | None | | None | | SAME |
| 42 | A | Susceptible | | None | | None | | None | | V35T, K49R, V60I, K122V, I135T, K173S, Q174K, D177E, V179I, T200A, I202V, Q207A, R211S | A | Susceptible | | None | | None | | None | | SAME |
| 43 | D | Susceptible | | None | | None | | None | | V35T, V60I, K104N, K122E, I142V, A158S, D177E, Q207G, R211K | D | Susceptible | | None | | None | | None | | SAME |
| 44 | A | Susceptible | | None | | None | | None | | V35T, V60I, D121H, I135T, K173S, Q174K, D177E, V179I, T200A, Q207A, R211S | A | Susceptible | | None | | None | | None | | SAME |
| 45 | A | Susceptible | | None | | None | | None | | V35T, T39L, V60I, D121H, K122E, I135T, K173L, Q174K, D177E, V179I, Q207A, R211S | A | Susceptible | | None | | None | | None | | SAME |
| 46 | A | Susceptible | | **V179T** | | None | | None | | V35T, K49R, V60I, K122E, D123G, I135T, K173L, Q174K, D177E, I178V, I202V, Q207D, R211S, F214L | A | Susceptible | | None | | None | | None | | SAME |
| 47 | D | Susceptible | | None | | None | | None | | V35T, T39I, E40D, K49R, V60I, D121Y, K122E, I135T, F171Y, K173L, Q174K, D177E, V179I, Q207A, R211S | D | Susceptible | | None | | None | | None | | SAME |
| 48 | A | Susceptible | | None | | None | | None | | V35T, V60I, K122E, D123S, I135R, E169D, K173S, Q174K, D177E, G196E, Q207A, R211S | A | Susceptible | | None | | None | | None | | SAME |
| 49 | A | Susceptible | | None | | None | | None | | V60I, D121Y, K122E, I135IT, S162H, K173L, Q174K, D177E, V179I, Q207A, R211S | A | Susceptible | | None | | None | | None | | SAME |
| 50 | CRF02_AG | **NNRTI** | | **K103N**, **F227FL** | | None | | None | | V60I, V90I, I135A, I142V, S162A, K173KT, Q174E, T200A, Q207E, L228Q, V245Q, D250E | CRF02_AG | **NNRTI** | | **K103N**, **F227FL** | | None | | None | | SAME |
| 51 | A | Susceptible | | None | | None | | None | | V35T, T39Q, S105A, K122E, D123N, I135T, K173A, D177G, V179I, T200A, I202V, Q207A, R211Q | A | Susceptible | | None | | None | | None | | SAME |
| 52 | A | **NNRTI, NRTI** | | **K101E**, **E138A**, **G190A** | | **F77FL**, **T215TAS** | | M184V | | Q85P, K122E, D123N, I135T, I142V, K173S, Q174K, V179I, T200A, Q207A, R211S, F214FL | A | **NNRTI, NRTI** | | **K101E**, **E138A**, **G190A** | | **F77L** | | M184V | | T215TAS |
| 53 | D | Susceptible | | None | | None | | None | | V35T, E40D, I47L, K49R, V60I, D121C, K122E, D123E, R125RK, K173E, D177E, T200A, Q207E | D | Susceptible | | None | | None | | None | | SAME |
| 54 | C+D | Susceptible | | None | | None | | None | | V35I, K49R, K102KE, K122E, D123S, I135IV, S162A, D177E, I178M, V179I, T200A, E204EK, Q207EG, R211K | C+D | Susceptible | | None | | None | | None | | SAME |
| 55 | A | Susceptible | | None | | None | | None | | V35T, T39Q, S105A, K122E, D123N, I135T, K173A, D177G, V179I, T200A, I202V, Q207A, R211Q | A | Susceptible | | None | | None | | None | | SAME |
| 56 | A | **NNRTI, NRTI** | | **A98G**, **Y181C** | | **L210*W, T215HNY** | | **M184V**, | | K122E, D123S, T128TP, I135T, T139R, K173L, Q174*K, N175NFIY, P176PFLS, D177*E, I178IK, V179I, T200A, I202IM, E203A, Q207A, R211S | A | **NNRTI, NRTI** | | **A98G**, **Y181C** | | **L210W** | | None | | **T215HNY** |
| 57 | A | Susceptible | | None | | None | | None | | V35T, K104R, D121H, I135T, S162Y, K173L, Q174K, D177E, Q207A, R211S | A | Susceptible | | None | | None | | None | | SAME |
| 58 | A | **NNRTI, NRTI** | | **A98G**, **K101E**, **V106VI**, **V179T**, **Y181C**, **G190S** | | None | | **M184V** | | D121H, K122E, K173A, Q174K, D177E, I178M, G196E, T200A, I202IM, E203EDGV, Q207A, R211N | A | **NNRTI, NRTI** | | **A98G**, **K101E**, **V106VI**, **V179T**, **Y181C** | | None | | **M184V** | | SAME |
| 59 | B | Susceptible | | None | | None | | None | | V35T, T39M, V60I, D121Y, K122E, T131TP, E169EK, Q174K, D177E, I178M, Q207E | B | Susceptible | | None | | None | | None | | SAME |
| 60 | C | Susceptible | | None | | None | | None | | V35T, E36A, T39D, S48T, V111VG, K166R, K173A, Q174K, D177E, I178M, D192N, I195M, G196M, T200A, Q207E | C | Susceptible | | None | | None | | None | | SAME |
| 61 | D | **NNRTI, NRTI** | | **V108I**, **Y181YC** | | **T215TN** | | None | | K32E, V35T, T39L, V60I, D121Y, K122E, D177E, R206RK, Q207E, R211K | D | **NNRTI** | | **V108I**, **Y181YC** | | None | | None | | **T215TN** |
| 62 | A | **NNRTI** | | **G190A** | | None | | None | | V35T, V60I, K122E, D123S, I135T, I142V, F171Y, K173S, Q174K, D177E, V179I, T200A, Q207A, R211S | A | **NNRTI** | | **G190A** | | None | | None | | SAME |
| 63 | C + D | **NNRTI, NRTI** | | **V106A** | | None | | **M184V** | | V35I, K49R, K102E, K122E, D123S, S162A, T165K, D177E, I178M, V179I, T200A, Q207G, R211K | C + D | **NNRTI, NRTI** | | **V106A** | | None | | **M184V** | | SAME |
| 64 | A | **NNRTI** | | **G190A** | | None | | None | | V35T, E40D, K122E, D123N, Q161H, K173S, Q174K, P176T, D177E, V179I, T200A, I202V, Q207A, R211S, F214L | A | **NNRTI** | | **G190A** | | None | | None | | SAME |
| 65 | A2 | **NNRTI, NRTI** | | **Y188L** | | None | | **M184V** | | V35T, T39L, K122E, D123E, D177E, I178M, T200A, Q207E, R211K | A2 | **NNRTI, NRTI** | | **Y188L** | | None | | **M184V** | | SAME |
| 66 | A | **NNRTI** | | **G190A** | | None | | None | | K32KR, V35T, T39A, E40D, K122E, D123N, K173S, Q174K, P176T, D177E, V179I, T200E, I202V, Q207A, R211S, F214L, P226PL | A | **NNRTI** | | **G190A** | | None | | None | | SAME |
| 67 | A | Susceptible | | None | | None | | None | | I31IK, V35T, E40D, V60I, K102R, K104R, D121Y, K122E, I135T, K173S, Q174K, D177E, V179I, T200A, Q207A, R211S | A | Susceptible | | None | | None | | None | | SAME |
| 68 | A | Susceptible | | None | | None | | None | | V35T, T39L, K122E, D123E, D177E, I178M, T200A, Q207E, R211K | A | Susceptible | | None | | None | | None | | SAME |
| 69 | A | **NNRTI** | | **G190A** | | None | | None | | V35T, T39A, E40D, K122E, D123N, R125RK, K173S, Q174K, P176T, D177E, V179I, T200A, I202V, Q207A, R211S, F214L | A | **NNRTI** | | **G190A** | | None | | None | | SAME |
| 70 | C + D | Susceptible | | None | | None | | None | | V35IT, K49R, I50IV, P55PS, V60VI, K102KE, K122E, D123S, I135IV, S162A, D177E, I178M, V179I, T200A, E204EK, Q207EG, R211K | C + D | Susceptible | | None | | None | | None | | SAME |
| 71 | A | Susceptible | | None | | None | | None | | V35T, T39Q, S105A, V111VG, K122E, D123N, R125RK, T131TP, I135T, V148VG, K173A, D177G, V179I, E194EK, R199RK, T200A, I202V, Q207A, R211Q, D218DN | A | Susceptible | | None | | None | | None | | SAME |
| 72 | D | Susceptible | | None | | None | | None | | V35PST, E36D, K49R, V60I, D121CHRY, K122E, I135IR, D177E, I178M, Q207E, R211K | D | Susceptible | | None | | None | | None | | SAME |
| 73 | A | Susceptible | | None | | None | | None | | V35T, V60I, K122E, D123N, R125RK, I135T, K173L, Q174K, D177E, V179I, I195L, T200A, Q207A, R211S | A | Susceptible | | None | | None | | None | | SAME |
| 74 | A | Susceptible | | None | | None | | None | | V35PST, V60I, K122E, D123S, I135T, K173A, Q174K, D177E, V179I, T200A, Q207A | A | Susceptible | | None | | None | | None | | SAME |
| 75 | A | Susceptible | | None | | None | | None | | V35T, T39K, E53D, K122E, D123N, I135T, T165I, K173S, Q174K, D177E, V179I, I202V, Q207A, R211N | A | Susceptible | | None | | None | | None | | SAME |
| 76 | D | **NRTI** | | None | | None | | **Y115YF** | | V35T, E40D, K49R, V60VI, V75VL, D121DN, K122E, D123N, R125RK, D177E, I178IM, E194EK, Q207E, R211K, D218DN | D | **NRTI** | | None | | None | | **Y115YF** | | SAME |
| 77 | A | Susceptible | | None | | None | | None | | V35T, K49R, K122E, D123N, I135T, K173S, Q174K, D177E, V179I, T200E, Q207A, R211S | A | Susceptible | | None | | None | | None | | SAME |
| 78 | A | Susceptible | | None | | None | | None | | V35T, V60I, K122E, D123S, I135T, K173A, Q174K, D177E, V179I, Q197P, T200A, Q207A, R211S, F214L | A | Susceptible | | None | | None | | None | | SAME |
| 79 | A | Susceptible | | None | | None | | None | | T39K, K122E, D123N, K173S, D177E, V179I, T200A, I202V, Q207S, R211K | A | Susceptible | | None | | None | | None | | SAME |
| 80 | A | Susceptible | | None | | None | | None | | V35T, T39A, E40D, V60I, K122E, D123S, I135T, K173S, Q174K, D177E, V179I, T200A, Q207A, F214L | A | Susceptible | | None | | None | | None | | SAME |
| 81 | A | Susceptible | | None | | None | | None | | T39K, K122E, D123N, K173S, D177E, V179I, T200A, I202V, Q207S, R211K | A | Susceptible | | None | | None | | None | | SAME |
| 82 | D | Susceptible | | None | | None | | None | | V35T, V60I, K104N, K122E, I142V, A158S, D177E, Q207G, R211K | D | Susceptible | | None | | None | | None | | SAME |
| 83 | C | Susceptible | | None | | None | | None | | V35T, E36S, T39E, E40D, K43KR, S48T, K49R, I50V, V60I, K122E, D123S, I135T, K173A, D177E, T200A, Q207E | C | Susceptible | | None | | None | | None | | SAME |
| 84 | A | **NRTI** | | None | | None | | **M184V** | | V35T, T39K, K43A, V60I, D121H, K122E, D123S, K173A, Q174K, D177E, I178M, V179VI, T200E, E204Q, Q207A, R211K | A | **NRTI** | | None | | None | | **M184V** | | SAME |
| 85 | D | Susceptible | | None | | None | | None | | V35T, K49R, V60I, D121Y, K122E, K173KQR, D177E, I180IV, E194D, T200TI, Q207E, R211K | D | Susceptible | | None | | None | | None | | SAME |
| 86 | B | Susceptible | | None | | None | | None | | V35T, T39A, E40D, K49R, V60I, D121Y, K122E, D123DN, I142IV, D177DE, V179VI, E203D, Q207E, R211K | B | Susceptible | | None | | None | | None | | SAME |
| 87 | C | Susceptible | | None | | None | | None | | V35T, E36A, T39E, E40D, K49R, K122E, D123N, A158S, K173I, Q174K, T200A, Q207D, R211K | C | Susceptible | | None | | None | | None | | SAME |
| 88 | A | Susceptible | | None | | None | | None | | V35T, V60I, K122E, D123NS, I135IT, K173S, Q174K, D177E, V179I, I202V, Q207D, R211S | A | Susceptible | | None | | None | | None | | SAME |
| 89 | B | **NNRTI** | | **G190A**, **F227FL** | | None | | None | | S48T, V60I, K122E, D123S, I135T, T139A, D177E, Q207E, R211K | B | **NNRTI** | | **G190A** | | None | | None | | F227FL |
| 90 | A | Susceptible | | None | | None | | None | | V35T, T39M, V60I, K64R, D121H, K122E, I135T, A158S, S162A, K173S, Q174K, D177E, I178L, I180IM, T200A, Q207A, R211S | A | Susceptible | | None | | None | | None | | SAME |
| 91 | D | **NNRTI, NRTI** | | **V108I**, **Y181YC** | | **T215TN** | | None | | V35T, T39I, E40D, K49R, V60I, W88WG, D123E, D177E, T200A, E204Q, Q207A | D | **NNRTI, NRTI** | | **V108I**, **Y181YC** | | None | | None | | **T215TN** |
| 92 | A | Susceptible | | None | | None | | None | | E28A, V35T, T39L, K122E, D123E, T131TP, I135T, K173S, Q174K, D177E, V179I, T200A, Q207A, R211S | A | Susceptible | | None | | None | | None | | SAME |
| 93 | D | Susceptible | | None | | None | | None | | V35T, T39M, K122E, D123G, I135T, K166R, D177E, G196E, T200A, Q207E, R211K | D | Susceptible | | None | | None | | None | | SAME |
| 94 | A | Susceptible | | None | | None | | None | | V35T, V60I, K122E, D123S, I135T, K173S, Q174K, D177E, V179I, T200E, Q207A, R211S, F214L | A | Susceptible | | None | | None | | None | | SAME |
| 95 | A | Susceptible | | None | | None | | None | | V35T, T39K, V60I, K122E, D123N, K173A, Q174K, D177E, V179I, T200TA, I202V, Q207A, R211S | A | Susceptible | | None | | None | | None | | SAME |
| 96 | A | **NRTI** | | None | | None | | **M184MV** | | V35T, T39K, K43A, V60I, D121H, K122E, D123S, K173A, Q174K, P176PS, D177E, I178M, V179VI, T200E, E204Q, Q207A, R211K | A | **NRTI** | | None | | None | | **M184MV** | | SAME |
| 97 | A | **NNRTI** | | **K103N** | | None | | None | | V35T, V60I, K64R, D121H, K122E, I135T, K173S, Q174K, D177E, V179S, T200A, Q207A, R211S, V245K | A | **NNRTI** | | **K103N** | | None | | None | | SAME |
| 98 | D | Susceptible | | None | | None | | None | | V35T, E36D, K49R, V60I, D121C, K122E, D177E, I178M, Q207E | D | Susceptible | | None | | None | | None | | SAME |
| 99 | D | Susceptible | | None | | None | | None | | I31IKN, A33S, L34T, V35T, E40D, K49R, V60I, K122E, D123N, Q174QK, D177E, I178M, R206RK, Q207E, H208HP, R211K | D | Susceptible | | None | | None | | None | | SAME |
| 100 | C | **NNRTI** | | **K103N**, **V106I** | | None | | None | | V35T, T39R, K43E, S48T, K102R, K122E, I142V, K166R, K173A, Q174R, D177E, I195L, G196K, T200A, Q207E, R211K | C | **NNRTI** | | **K103N**, **V106I** | | None | | None | | SAME |
